# The genome-scale metabolic model *iIN800 *of *Saccharomyces cerevisiae *and its validation: a scaffold to query lipid metabolism

**DOI:** 10.1186/1752-0509-2-71

**Published:** 2008-08-07

**Authors:** Intawat Nookaew, Michael C Jewett, Asawin Meechai, Chinae Thammarongtham, Kobkul Laoteng, Supapon Cheevadhanarak, Jens Nielsen, Sakarindr Bhumiratana

**Affiliations:** 1 Department of Chemical Engineering, Faculty of Engineering, King Mongkut's University of Technology Thonburi, Bangkok 10140, Thailand; 2 Biochemical Engineering and Pilot Plant Research and Development Unit, National Center for Genetic Engineering and Biotechnology (BIOTEC) at King Mongkut's University of Technology Thonburi, Bangkhuntien, Bangkok 10150, Thailand; 3 School of Bioresources and Technology, King Mongkut's University of Technology Thonburi, Bangkok 10140, Thailand; 4 National Science and Technology Development Agency, Ministry of Science and Technology, Thailand Science Park, Klong Luang, Pathumthani 12120, Thailand; 5 Center for Microbial Biotechnology, Biocentrum, Technical University of Denmark, DK-2800 Kgs. Lyngby, Denmark; 6 Department of Genetics, Harvard Medical School, Boston, MA 02115, USA; 7 Department of Chemical and Biological Engineering, Chalmers University of Technology, SE-412 96 Gothenburg, Sweden

## Abstract

**Background:**

Up to now, there have been three published versions of a yeast genome-scale metabolic model: *iFF708*, *iND750 *and *iLL672*. All three models, however, lack a detailed description of lipid metabolism and thus are unable to be used as integrated scaffolds for gaining insights into lipid metabolism from multilevel omic measurement technologies (e.g. genome-wide mRNA levels). To overcome this limitation, we reconstructed a new version of the *Saccharomyces cerevisiae *genome-scale model, *iIN800 *that includes a more rigorous and detailed description of lipid metabolism.

**Results:**

The reconstructed metabolic model comprises 1446 reactions and 1013 metabolites. Beyond incorporating new reactions involved in lipid metabolism, we also present new biomass equations that improve the predictive power of flux balance analysis simulations. Predictions of both growth capability and large scale *in silico *single gene deletions by *iIN800 *were consistent with experimental data. In addition, ^13^C-labeling experiments validated the new biomass equations and calculated intracellular fluxes. To demonstrate the applicability of *iIN800*, we show that the model can be used as a scaffold to reveal the regulatory importance of lipid metabolism precursors and intermediates that would have been missed in previous models from transcriptome datasets.

**Conclusion:**

Performing integrated analyses using *iIN800 *as a network scaffold is shown to be a valuable tool for elucidating the behavior of complex metabolic networks, particularly for identifying regulatory targets in lipid metabolism that can be used for industrial applications or for understanding lipid disease states.

## Background

The yeast *Saccharomyces cerevisiae *is widely used for production of many different commercial compounds such as food, feed, beverages and pharmaceuticals [1]. It also serves as a model eukaryotic organism and has been the subject of more than 40,000 research publications [2,3]. After the complete genome sequence for yeast was released in 1996 [4], about 4,600 ORFs were characterized [3] and yeast contains many genes with human homologs [2]. This has allowed for comparative functional genomics and comparative systems biology between yeast and human. Yeast, for example, has been used to understand the function of complex metabolic pathways that are related to the development of human diseases [5-7].

Several human diseases (e.g. cancer, atherosclerosis, Alzheimer's disease, and Parkinson's disease) are associated with disorders in lipid metabolism [8-10]. The emergence of lipidomics has allowed analysis of lipid metabolism at the systems level [8,11]. Lipidomics promises to make a significant impact in our understanding of lipid related disease development [12]. As with other high-throughput techniques, however, we hypothesize that one of the main challenges for utilization of lipidome data will be our ability to develop appropriate frameworks to integrate and map data for studying relations between lipid metabolism and other cellular networks.

Previous work has shown that genome-scale metabolic models provide an excellent scaffold for integrating data into single, coherent models [13]. The calculation of Reporter Metabolites using genome-scale metabolic models is an example of how metabolic models can be used to upgrade the information content of omics data [14]. This approach allows mapping of key metabolites and reactions in large metabolic networks when combined with transcriptome [14] or metabolome data [15]. However, pathways, reactions, and genes that are not included in the metabolic network cannot be queried. Therefore, the Reporter Metabolite algorithm requires a reliable and global genome scale-model to achieve precise and accurate data interpretation.

So far, three yeast genome-scale metabolic models, *iFF708*, *iND750 *and *iLL672*, have been published. All three models, however, lack a detailed description of the lipid metabolism. The first model, *iFF708 *[16], consists of 1175 reactions linked to 708 ORFs. *iFF708 *shows good predictions of many different cellular functions [17] and gene essentiality predictions [18]. However, almost all intermediate reactions in lipid metabolism were either lumped or neglected. The second model published was *iND750 *[19]. *iND750 *is fully compartmentalized, consisting of 1498 reactions linked to 750 ORFs. The model was validated by a large-scale gene deletion study and metabolic phenotypes [20] and was expanded to include regulation for predicting gene expression and phenotypes of different transcription factor mutants [21]. *iND750 *contains more reactions and metabolites in lipid metabolism than *iFF708*, but still lacks a comprehensive description of lipid metabolism. The third published model is *iLL672*, which is derived from *iFF708 *and comprises 1038 reactions. Several dead-end reactions of *iFF708 *were eliminated leading to an improved accuracy of the single gene deletion prediction [22]. However, only minor improvements were made to reactions involved in lipid metabolism. The model was validated using ^13^C-labeling experiments to study the robustness of different yeast mutants [23].

Here our objective was to expand the genome-scale metabolic model of yeast to include a detailed description of lipid metabolism for use as a scaffold to integrate omics data. We used *iFF708 *as a template for building a model based on recent literature that contains new reactions in lipid metabolism and transport relative to all previous models. The new model named *iIN800 *includes 92 additional ORFs and provides a more detailed structure of lipid metabolism, tRNA synthesis and transport processes than previous models. The biomass composition, which is very important for flux balance analysis and predicting lethality, was also recalculated and improved. *iIN800 *was validated with large-scale gene deletion data and growth simulation predictions. Simulated intracellular fluxes were also supported by ^13^C-labeling flux experimental data. Finally, we show that the transcriptome data of yeast cultivated under various growth conditions can be integrated with *iIN800 *to identify lipid related Reporter Metabolites. We anticipate that *iIN800 *will be useful as a scaffold for integrating multilevel omic data and that this new model will have a significant impact in the emerging field of lipidomics.

## Results and discussion

### Model reconstruction and characteristics of *iIN800*

Due to the complexity of compartmentalization used in *iND750 *and the smaller scope of *iLL672*, the metabolic model *iFF708 *was selected as a template for the development of the model *iIN800*. Pathway and reaction databases (e.g. KEGG), online resources (e.g. SGD), and literature were used to expand *iFF708*, with particular focus on lipid metabolism. *iIN800 *contains 340 total reactions in lipid metabolism, more than at least 143 reactions greater than previous models (Table 1).

**Table 1:** Comparison of the number of lipid metabolism reactions among yeast genome-scale metabolic models

**Model**	***iFF708***	***iLL672***	***iND750***	***iIN800***
	
**Mitochondrial fatty acid synthesis**	14	*0*	13	45
**Cytosolic fatty acid synthesis**	17	*18*	27	48
**Fatty acid elongation**	0	*4*	2	33
**Fatty acid activation and beta-oxidation**	9	*19*	53	65
**Sphingolipid synthesis**	18	*23*	37	27
**Phospholipid and TAG synthesis**	37	*37*	35	68
**Ergosterol biosynthesis**	31	*28*	30	29
**Ergosterol esterification**	0	*0*	0	2
**Lipid degradation**	0	*0*	0	23
	
**Total**	**126**	***129***	**197**	**340**

To compare metabolic characteristics of the different *in silico *models, lipid metabolism was classified into unique sub-categories (e.g. mitochondrial fatty acid synthesis, ergosterol biosynthesis) (Table 1). Fatty acid synthesis and elongation accounted for three of these sub-categories. In contrast to previous models, *iIN800 *incorporates fatty acid biosynthesis in both mitochondria and the cytosol. Fatty acid synthesis, which involves iterative malonyl-CoA condensations that result in a growing chain of fatty acids, is catalyzed by four major enzymes: β-ketoacyl-ACP synthase (a condensing enzyme), β-ketoacyl-ACP reductase, β-dehydroxyacyl-ACP dehydratase and enoyl-ACP reductase. In the cytosol, these enzymes are encoded by the multifunctional *FAS1 *and *FAS2*. In the mitochondria, however, fatty acid synthesis is carried out by the products encoded by *CEM1, OAR1, HTD2 *and *ETR1*. These ORFs were missing from previous models, which prevented simulation of mitochondrial fatty acid synthesis. Fatty acid elongation, which leads to the production of long-chain fatty acids, was not included in *iFF708*, but was also updated in *iIN800*. Including fatty acid elongation resulted in the addition of four major biochemical reaction steps: condensing enzyme, 3-ketoacyl-CoA reductase, enoyl-CoA dehydratase and enoyl-CoA reductase [24]. These reactions are carried out by the enzymes encoded by *ELO1, ELO2, ELO3, IFA38 *and *TSC13*. While the gene encoding enoyl-CoA dehydratase has not been identified in *S. cerevisiae*, the reaction was inferred due to the identification of long chain fatty acids in yeast.

β-oxidation is the process where fatty acids, after becoming activated in the form of acyl-CoAs, are broken down to make acetyl-CoA, and ultimately energy. *FAT1*, encoding an enzyme for long-chain fatty acid activation was missing in *iFF708 *and *iLL672*. The genes *SPS19, ECI1 *and *DCI1 *are also now included in *iIN800*. As a result, *iIN800 *can simulate the oxidation of unsaturated fatty acids.

Sphingolipid synthesis reactions were added to *iIN800 *according to a recently reported model [25], resulting in more sphingolipid reactions than the template *iFF708*. Sphingolipid synthesis is the only sub-category in *iIN800 *with a significantly lower reaction tally than *iND750*. This is because *iND750 *incorporated both C24:0 and C26:0 as very long-chain fatty acids (the back bone of sphingolipids) to produce ceramides. Because the amount of very long chain fatty acids in *S. cerevisiae *is so low relative to other fatty acid species (<2% of total fatty acid pool) [24,26], *iIN800 *treats very long chain fatty acids as a single metabolite. As a result, fewer reactions are present in sphingolipid synthesis.

Relative to other models, only minor changes in the biosynthesis of phospholipids and triacylglycerides as well as ergosterol were introduced in *iIN800*. However, esterification of sterols and degradation of lipids, which were not included in all other previous models, are present in *iIN800 *(Table 1). Finally, 26 ORFs encoding for tRNA synthesis and one related enzyme, lipoamide dehydrogenase as well as 14 ORFs encoding transporters were also included in *iIN800*. The additionally included ORFs and their related references as well as detailed comparisons of reactions in lipid metabolism of all reported models are given in Additional files 1 and 2, respectively.

**Figure 1:**
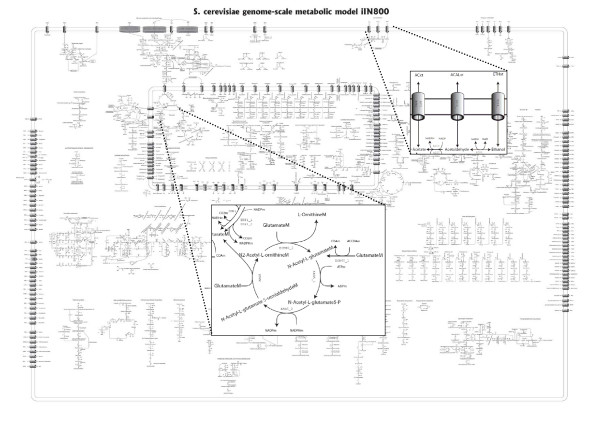
The reconstructed *S. cerevisiae *genome-scale metabolic model *iIN800*.

In summary, *iIN800 *was reconstructed from 17.2% of the characterized ORFs in yeast and contains 1446 metabolic reactions and 1013 metabolites in total. This model is relatively more comprehensive as compared with previously described models (Table 2). The network characteristics of *iIN800 *and the starting model *iFF708 *are shown in Table 3. Within lipid metabolism, we have incorporated many new reactions in mitochondrial fatty acid synthesis, cytosolic fatty acid synthesis, fatty acid elongation, fatty acid activation and β-oxidation, sphingolipid synthesis, ergosterol esterification, and lipid degradation (Table 1). 96 new reactions are derived from biochemical and physical considerations. These reactions mostly describe transportation of fatty acids and lipids across the mitochondria and the plasma membrane. To visualize the model *iIN800*, we constructed a comprehensive metabolic map using ReMapper software (Figure 1). This visualized map provides a method for globally plotting transcript and flux data onto *iIN800*. The source file is available for download (see Methods).

**Table 2:** Structure comparison of *S. cerevisiae *genome-scale metabolic models

**Model**	**Genes**	**Reactions**	**Metabolites**
***iFF708***	708(15.2%)*	1175	825
***iLL672***	672(14.1%)*	1038	636
***iND750***	750(16.1%)*	1489	972
***iIN800***	800(17.2%)*	1446	1013

* percentage of associated ORFs in the model relative to characterized ORFs in the yeast genome

**Table 3:** Network characteristics of the reconstructed metabolic network of *S. cerevisiae *strain *iFF708 *and *iIN800*

**Model**	***iFF708***	***iIN800***
**Metabolites**	**825**	**1013**
Cytosolic metabolites	518	631
Mitochondrial metabolites	170	228
Extracellular metabolites	137	154

**Reactions**	**1175**	**1446**
Mitochondrial reactions	104	161
Cytosolic reactions	723	906

**Exchange fluxes**	**348**	**379**
Cytosolic exchange fluxes	286	304
Mitochondrial exchange fluxes	62	75

**Reactions with ORF assignments**	1075	1209
**Biochemical and Physical consideration**	140	237

### Improved biomass equation

The biomass equation is crucial for using genome-scale models to simulate growth using flux balance analysis (FBA). Therefore, an important consideration in the development of *iIN800 *was to address the concern that the biomass composition of *S. cerevisiae *changes under different growth conditions. For example, during growth on excess glucose the carbohydrate content increases and during growth on excess ammonium the protein content increases.

**Figure 2:**
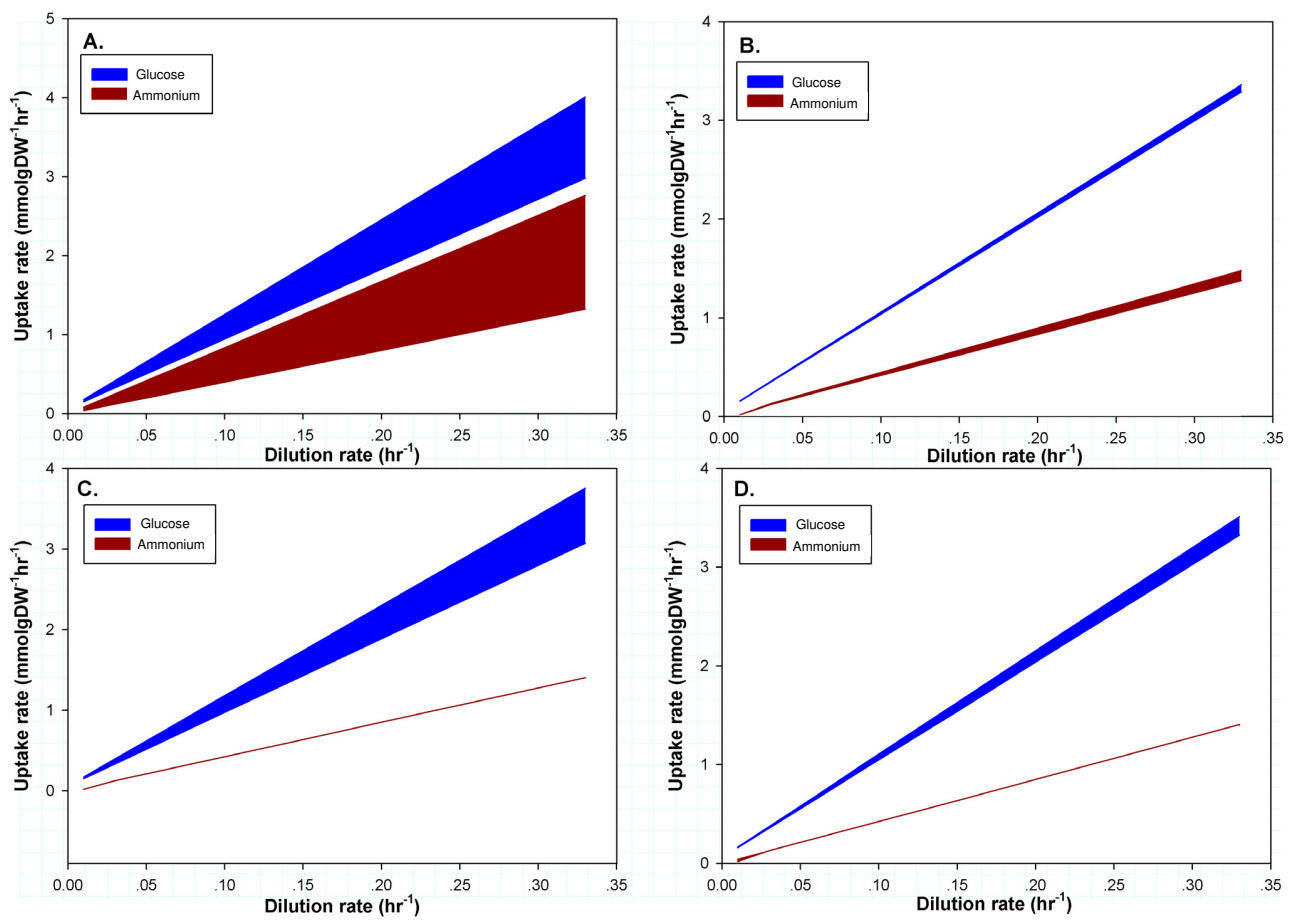
**Sensitivity analysis shows the influence of macromolecular composition on the simulated growth rate using *iIN800*.** The simulations were performed for aerobic glucose- and ammonium-limited cultivations by varying (A) the protein content (35–65%), (B) the RNA content (3.5–12%), (C) the carbohydrate content (15–50%) and (D) the lipid content (2 – 15%).

To assess the sensitivity of flux simulations using *iIN800 *towards changes in the macro-molecular composition, we performed constraint-based simulations by varying the protein, RNA, carbohydrate and lipid content of the biomass in physiological relevant ranges based on previous experimental reports [27-29], from 35–65%, 3.5–12%, 15–50% and 2–15%, respectively. Specifically, glucose and ammonium uptake rates were minimized for both glucose- and ammonium-limited growth conditions, respectively, using different macromolecular compositions at fixed growth rates, (note: this is the same mathematical problem as fixing uptake rates and maximizing growth rate). In this way, we could compare the differences between glucose- and ammonium-limited growth conditions. The results are illustrated in Figure 2. An interesting finding was that the protein content strongly affects the uptake rates at both glucose- and ammonium-limited conditions, albeit to a greater extent in ammonium-limited conditions (Fig. 2A). The carbohydrate content on the other hand does not have an impact on the ammonium uptake rate, it strongly impacts the glucose uptake rate (Fig. 2C). The RNA content and the lipid content have only a minor impact on growth (Figures 2B and 2D).

In summary, the sensitivity analysis shows that the biomass composition can significantly impact predictions made with genome-scale metabolic models to varying degrees based on different growth conditions. We therefore present new biomass equations to be used under C-limited and N-limited growth conditions, respectively. These compositions result from previous studies and our own measurements of lipids and fatty acids across multiple N-limited and C-limited growth conditions (data not shown). Using a separate biomass composition for N-limited cultures has not been proposed previously. The N-limited biomass equation is therefore new. Relative to previous C-limited biomass compositions, the most dramatic changes in our here proposed biomass equation is with respect to the lipids and fatty acids (Table 4). While our sensitivity analysis suggests that these components will most likely only lead to a small improvement in the accuracy of C-limited flux simulations, they may play an important role in lethality prediction by the model, as the addition of extra components in the biomass equation will give a higher resolution.

**Table 4:** Biomass composition

**Metabolites**	**Amount (mmol/gDW)**	
**Amino acids**	**Carbon-limited**	**Nitrogen-limited**
L-Alanine	0.357	0.252
L-Arginine	0.136	0.098
L-Asparagine	0.172	0.153
L-Aspartate	0.172	0.153
L-Cysteine	0.043	0.044
L-Glutamate	0.268	0.231
L-Glutamine	0.268	0.231
Glycine	0.325	0.278
L-Histidine	0.075	0.071
L-Isoleucine	0.172	0.142
L-Leucine	0.250	0.207
L-Lysine	0.239	0.204
L-Methionine	0.050	0.044
L-Phenylalanine	0.114	0.092
L-Proline	0.129	0.118
L-Serine	0.254	0.225
L-Threonine	0.197	0.160
L-Tryptophan	0.027	0.028
L-Tyrosine	0.096	0.068
		
**Carbohydrates**	**Carbon-limited**	**Nitrogen-limited**
Glycogen	0.519	0.667
alpha,alpha-Trehalose	0.023	0.085
Mannan	0.821	0.994
1,3-beta-D-Glucan	1.136	0.963
		
**RNA**	**Carbon-limited**	**Nitrogen-limited**
AMP	0.051	0.040
GMP	0.051	0.040
CMP	0.050	0.039
UMP	0.067	0.052
		
**DNA**	**Carbon-limited**	**Nitrogen-limited**
dAMP	0.004	0.004
dCMP	0.002	0.003
dTMP	0.004	0.004
dGMP	0.002	0.003
		
**Lipids**	**Carbon-limited**	**Nitrogen-limited**
Phosphatidylcholine	0.002884	0.001660
1-Phosphatidyl-D-myo-inositol	0.001531	0.001656
Phosphatidylserine	0.000373	0.000302
Phosphatidylethanolamine	0.000697	0.000083
Acyl_acids	0.000206	0.000723
Triacylglycerol	0.000781	0.003618
Ergosterol-ester	0.000812	0.004632
Ergosta-5,7,22,24(28)-tetraenol	0.000125	0.000167
Ergosterol	0.005603	0.005155
Zymosterol	0.000015	0.000051
Episterol	0.000096	0.000062
Fecosterol	0.000114	0.000068
Lanosterol	0.000032	0.000074
4,4-Dimethylzymosterol	0.000056	0.000046
Ceramide-I	0.000351	0.000075
Ceramide-II	0.000066	0.000009

### Growth simulation capability

*In silico *genome-scale models are most generally used to predict various phenotypes. These include growth rates and extracellular secretion rates of metabolite products, as well as uptake rates of nutrients. In addition, models can be employed to explore active route(s) in metabolic pathways under certain growth conditions as illustrated for a genome-scale metabolic model of *E. coli *[30-32] as well as for one of the *S. cerevisiae *genome-scale metabolic models [17].

**Figure 3:**
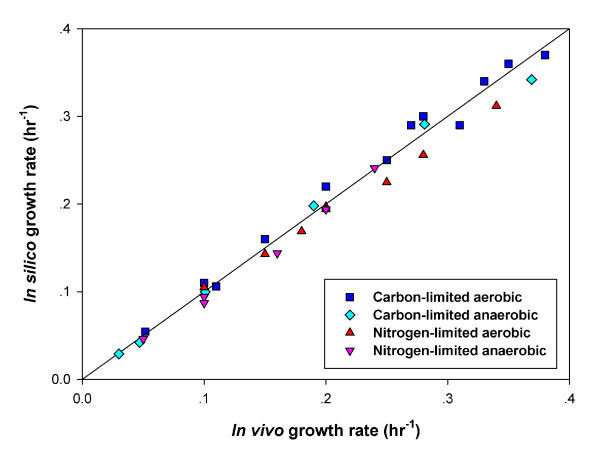
**Comparison demonstrating *in silico *and *in vivo *growth rates at various cultivation conditions.***In silico *predictions were performed using FBA with *iIN800*. Experimental measurements were taken from the literature (see text for references).

To validate *iIN800*, we first investigated the model's ability to simulate aerobic and anaerobic growth in glucose- or ammonium-limited conditions. Several published chemostat datasets were used as experimental references. As shown in Figure 3, the results from the computational growth prediction agreed with experimental measurements. Less than 10% relative error was observed (Figure 3). The details of the simulations and the corresponding reference data are given in Additional file 3. Intracellular fluxes can be easily visualized using the ReMapper software and our model (Additional files 4 and 5).

**Figure 4:**
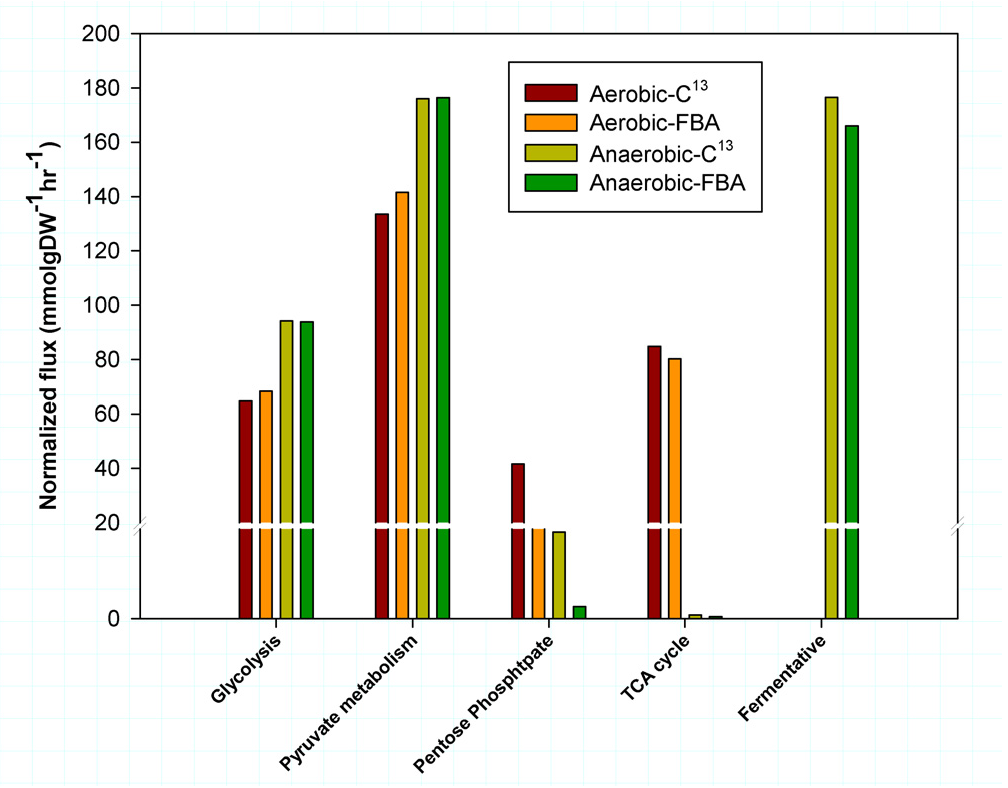
Comparisons of the major intracellular fluxes in the central metabolism calculated using FBA with *iIN800 *and ^13^C-labeling metabolic flux analysis at a dilution rate of 0.05 h^-1 ^of either aerobic or anaerobic glucose-limited conditions.

Since the new biomass equations would be expected to impact the overall flux distributions, we used ^13^C-flux analysis data to further confirm the computed intracellular fluxes. Specifically, fluxes in the central carbon metabolism at two different growth conditions were both measured by ^13^C-labeling experiments and calculated by FBA using *iIN800*. The model validation is shown in Figure 4. There is a high degree of agreement between the predicted and experimental fluxes in the central metabolism, with the exception of fluxes through the pentose phosphate pathway (PPP). Using FBA, the flux through the PPP is largely determined by the requirement for NADPH, and it has earlier been shown difficult to balance NADPH production and consumption [33]. This may explain why the FBA simulations under-predict the flux through this pathway.

### Evaluation of large-scale gene deletion

To verify further *iIN800*, we investigated the ability of the model to predict for growth viability due to a single gene deletion. *In silico *deletion phenotype predictions were examined for the new model with cells grown in both minimal media with a sole carbon source (glucose, galactose, glycerol and ethanol) and with rich media (YPD). *iIN800 *was assessed for its ability to make correct predictions based on experimental data [22,34]. A summary of the *in silico *single gene deletion predictions are given in Table 5. The overall prediction rate of *iIN800*, derived from 3392 total predictions, was 89.36%, with 95.50% sensitivity and 38.69% selectivity. The evaluation of the mean of a confusion matrix as the geometric mean of *iIN800 *equals 60.79%. The performance of the *iIN800 *model has improved by ~2% and ~7% in terms of overall prediction rate compared with the models *iFF708 *and *iND750*, respectively. We believe that the improvement is mainly due to upgrades in the biomass equation, which is consistent with results from Kuepfer *et al*. demonstrating that more accurate biomass compositions lead to improved lethality predictions [22]. The false predictions might be due to missing information in gene regulation, biomass compositions, dead-end reactions and medium composition, especially in the rich medium [18,19]

**Table 5:** Summary of large-scale single gene deletion evaluation of *S. cerevisiae iIN800*.

	Minimal media on					Rich media	
**Evaluation**	Glucose*	Glucose**	**Galactose**	**Glycerol**	**Ethanol**	**YPD**	**Total**
**TP**	468	469	461	461	463	567	**2889**
**TN**	23	23	20	17	21	38	**142**
**FP**	37	37	42	45	43	21	**225**
**FN**	14	13	19	19	15	56	**136**
**Number of deletions**	542	542	542	542	542	682	**3392**
**Positive prediction rate**	92.67	92.69	91.65	91.11	91.50	96.43	**92.77**
**Negative prediction rate**	62.16	63.89	51.28	47.22	58.33	40.43	**51.08**
**Accuracy**	90.59	90.77	88.75	88.19	89.30	88.71	**89.36**
**Sensitivity**	97.10	97.30	96.04	96.04	96.86	91.01	**95.50**
**Selectivity**	38.33	38.33	32.26	27.42	32.81	64.41	**38.69**
**Geometric mean**	61.01	61.07	55.66	51.32	56.38	76.56	**60.79**

* = Aerobic growth, ** = Anaerobic growth

TP = true positive, TN = true negative, FP = false positive, FN = false negative.

### Integration of transcriptome data with genome-scale metabolic models

Genome-scale metabolic models have shown promise for identifying Reporter Metabolites, defined as metabolites whose neighboring genes in a bipartite metabolic graph are most significantly affected and respond as a group to genetic or environmental perturbations [14]. Such an approach has previously been used to reveal important regulatory hot-spots in metabolism from genome-wide expression data and has demonstrated promise for integrating omic data using network topology. To highlight the importance and utility of having a more complete metabolic model in this integrated analysis, the genome-scale models *iIN800 *and *iFF708 *were used to calculate Reporter Metabolites. Multiple sets of transcriptome data were used for analysis. Lists of the top thirty most significant Reporter Metabolites for several perturbations are compared between *iIN800 *and *iFF708 *in Table 6, and Reporter Metabolites unique to *iIN800 *are marked in bold.

**Table 6:** Top thirty Reporter Metabolites calculated from various perturbations. The Reporter Metabolite algorithm was performed with *iIN800 *and *iFF708*.

**Oxidative phase**^1^		**Reductive building phase**^1^		**Reductive charging Phase**^1^	
***iIN800***	***iFF708***	***iIN800***	***iFF708***	***iIN800***	***iFF708***

AMP	IMP	AMPM	AMPM	Dodecanoyl-CoA*	Acyl-CoA
IMP	Xanthosine 5'-phosphate	PyrophosphateM	tRNAM	Decanoyl-CoA*	alpha,alpha-Trehalose
Pyrophosphate	L-Methionine	ATPM	PyrophosphateM	Trans-3-C16-CoA*	Glycogen
L-Methionine	5-Phospho-alpha-D-ribose 1-PP	tRNAM	Porphobilinogen	Trans-3-C18-CoA*	alpha,alpha'-Trehalose 6-phosphate
Xanthosine 5'-phosphate	L-Aspartate	H+M	L-TryptophanM	Trans-3-C14-CoA*	alpha-D-Glucose
ATP	Sulfate	NADHM	L-Tryptophanyl-tRNA(Trp)M	alpha,alpha-Trehalose	Oxalosuccinate
5-Phospho-alpha-D-ribose 1-PP	Homocysteine	Porphobilinogen	Dolichyl beta-D-mannosyl-P	Glycogen	3-Oxoacyl-CoA
L-Serine	AMP	Dolichyl beta-D-mannosyl-P	Mannan	alpha,alpha'-Trehalose 6-phosphate	a Long-chain carboxylic acid
L-Aspartate	H+EXT	L-Tryptophanyl-tRNA(Trp)M	Xanthine	Oxalosuccinate	Carnitine
H+EXT	3-Phosphonooxypyruvate	Mannan	L-Asparaginyl-tRNA(Asn)M	Trans-2-C14-CoA*	alpha-D-Glucose 6-phosphate
Homocysteine	N6-(L-1,3-Dicarboxypropyl)-L-lysine	tRNA(Ile)M*	H+M	Trans-2-C16-CoA*	UDPglucose
Sulfate	5,10-Methylenetetrahydrofolate	L-Isoleucyl-tRNA(Ile)M*	Dolichyl phosphate	Trans-2-C18-CoA*	Isocitrate
L-Glutamine	Aminoimidazole ribotide	tRNA(Thr)M*	all-trans-Nonaprenyl-PP	3-keto-Dodecanoyl-CoA*	D-Glucose 1-phosphate
L-Cysteine	L-Cystathionine	L-Threonyl-tRNA(Thr)M*	NADHM	3-keto-Decanoyl-CoA*	CoAM
L-Asparagine	L-Serine	Xanthine	ATPM	3-keto-Octanoyl-CoA*	Acetyl-CoAM
S-Adenosyl-L-methionine	Uracil	Dolichyl phosphate	D-Mannose 6-phosphate	3-keto-Hexanoyl-CoA*	CoA
Uracil	Sulfite	all-trans-Nonaprenyl-PP	UbiquinolM	3-keto-Butanoyl-CoA*	O-Acetylcarnitine
5,10-Methylenetetrahydrofolate	5-amino-4-imidazolecarboxylate	L-Asparaginyl-tRNA(Asn)M	Ubiquinone-9M	Dodecanoic_acid*	Succinate
3-Phosphonooxypyruvate	2-Hydroxybutane-1,2,4-tricarboxylate	tRNA(Phe)M*	CO2M	Carnitine	(S)-3-Hydroxy-3-methylglutaryl-CoA
N6-(L-1,3-Dicarboxypropyl)-L-lysine	S-Adenosyl-L-methionine	L-Phenylalanyl-tRNA(Phe)M*	Guanosine	alpha-D-Glucose	NAD+
L-Cystathionine	L-Asparagine	Intermediate_Methylzymosterol_II	IsocitrateM	Trans-2-4-diene-CoA*	H2O2
NH3	5-Phosphoribosylamine	Intermediate_Zymosterol_II	GTPM	Isocitrate	Malate
tRNA(Phe)*	GlycineM	UbiquinolM	GDPM	alpha-D-Glucose 6-phosphate	Maltose
L-Phenylalanyl-tRNA(Phe)*	Guanine	D-Mannose 6-phosphate	ITPM	UDPglucose	(3S)-3-Hydroxyacyl-CoA
Tetrahydrofolate	L-Histidine	Ubiquinone-9M	IDPM	D-Glucose 1-phosphate	GLCxt
Guanine	N1-(5'-phosphoribosyl)acetamidine	tRNA(Asp)M*	ITP	O-Acetylcarnitine	Glycerone phosphate
Sulfite	Tetrahydrofolate	L-Aspartyl-tRNA(Asp)M*	IDP	Tetradecanoyl-CoA*	O-AcetylcarnitineM
L-Histidine	alpha-D-Glutamyl phosphate	tRNA(Pro)M*	Phosphatidate	Decanoic_acid*	CarnitineM
5-amino-4-imidazolecarboxylate	HomoisocitrateM	L-Prolinyl-tRNA(Pro)M*	C100ACPm	(S)-3-Hydroxy-3-methylglutaryl-CoA	D-Galactose
GlycineM	GMP	Pyrophosphate	Dodecanoyl-ACPM	H2O2	SuccinateM

* Metabolite is contained in *iIN800 *only 1 = data from Tu, B. P., A. Kudlicki, et al. (2005)					

**Carbon- and Nitrogen-limited**^2^		**Aerobic and Anaerobic**^2^		**Temperature(30°C and 15°C)**^3^	

***iIN800***	***iFF708***	***iIN800***	***iFF708***	***iIN800***	***iFF708***

Glyoxylate	Glyoxylate	Oxygen	Ferricytochrome cM	IMP	IMP
GLUxt	L-Phenylalanine	Ferricytochrome cM	Ferrocytochrome cM	Tetrahydrofolate	Tetrahydrofolate
Isocitrate	GLUxt	Ferrocytochrome cM	Ubiquinone-9M	alpha,alpha-Trehalose	alpha,alpha-Trehalose
ALAxt	Isocitrate	Ubiquinone-9M	Oxygen	Hexadecanoyl-9-ene-CoA*	D-Erythrose 4-phosphate
Malate	ALAxt	UbiquinolM	UbiquinolM	Octadecanoyl-9-ene-CoA*	L-OrnithineM
Allantoate	Allantoate	ADPM	ADPM	Tetradecanoyl-9-ene-CoA*	Xanthosine 5'-phosphate
SERxt	Malate	H+M	H+M	D-Erythrose 4-phosphate	N6-(L-1,3-Dicarboxypropyl)-L-lysine
L-Alanine	L-Alanine	Dodecanoyl-CoA*	FADH2M	L-OrnithineM	NADH
Decanoyl-CoA*	SERxt	FumarateM	FumarateM	Xanthosine 5'-phosphate	URIxt
ASNxt	ASNxt	OrthophosphateM	OrthophosphateM	N6-(L-1,3-Dicarboxypropyl)-L-lysine	1-Phosphatidyl-1D-myo-inositol 4-P
GLNxt	GLNxt	FADH2M	Sphinganine 1-phosphate	URIxt	Homocysteine
ILExt	ILExt	Hexadecanoyl-9-ene-CoA*	ATPM	Homocysteine	1-Phosphatidyl-D-myo-inositol 4,5-PP
VALxt	VALxt	Octadecanoyl-9-ene-CoA*	Fumarate	Octadecanoyl-CoA	N-Acetyl-L-glutamateM
Trans-2-C161-CoA*	Ferricytochrome cM	Tetradecanoyl-9-ene-CoA*	Glyoxylate	N-Acetyl-L-glutamateM	Dihydrofolate
Trans-2-C181-CoA*	Ferrocytochrome cM	Sphinganine 1-phosphate	Isocitrate	Dihydrofolate	N2-Acetyl-L-ornithineM
Trans-2-C141-CoA*	PHExt	Phytosphingosine 1-phosphate	ERGOSTxt	N2-Acetyl-L-ornithineM	Anthranilate
PHExt	L-Asparagine	Tetradecanoyl-Co	ZYMSTxt	Anthranilate	S-Adenosyl-L-homocysteine
Ferricytochrome cM	Allantoin	Fumarate	NAD+	Hexadecanoyl-9-ene_acid*	UREAxt
Ferrocytochrome cM	LEUxt	Trans-2-C161-CoA*	FADM	Octadecanoyl-9-ene_acid*	L-Aspartate
FRUxt	FRUxt	Trans-2-C181-CoA*	6-Phospho-D-gluconate	S-Adenosyl-L-homocysteine	N(pai)-Methyl-L-histidine
Allantoin	Succinate	Trans-2-C141-CoA*	1,3-Diaminopropane	NADH	Adenosine 3',5'-bisphosphate
LEUxt	HISxt	Glyoxylate	sn-Glycerol 3-phosphate	UREAxt	4-imidazolecarboxylate
Succinate	TYRxt	sn-Glycerol 3-phosphate	O-Acetylcarnitine	L-Aspartate	3-Methyl-2-oxobutanoateM
HISxt	METxt	Isocitrate	Ethanol	1-Phosphatidyl-D-myo-inositol-3-P	Tetrahydrofolyl-[Glu](n)
Dodecanoyl-CoA*	GLYxt	ERGOSTxt	DIPEPxt	N(pai)-Methyl-L-histidine	2-Phenylacetamide
PROxt	ASPxt	ZYMSTxt	Dipeptide	Adenosine 3',5'-bisphosphate	Phenylacetic acid
alpha-D-Mannose	GLCxt	1,3-Diaminopropane	OPEPxt	L-Asparagine	Indole-3-acetamide
Trans-3-C16-CoA*	L-Tyrosine	6-Phospho-D-gluconate	Oligopeptide	C24-CoA*	Indole-3-acetate
Trans-3-C18-CoA*	PROxt	H2O2	PEPTxt	1-(5-Phospho-D-ribosyl)-5-amino	Urea-1-carboxylate
Trans-3-C14-CoA*	alpha-D-Mannose	Trans-3-C16-CoA*	Sphinganine	3-Methyl-2-oxobutanoateM	PhosphatidylserineM

* Metabolite is contained in *iIN800 *only

2 = Tai, S. L., V. M. Boer, et al. (2005)

3 = Pizarro, F., M.C. Jewett, et al. (2008)

First, transcriptome data from the yeast metabolic cycle [35] were analyzed. Notably, the reporter algorithm identified unique Reporter Metabolites with *iIN800 *that would have been missed if *iFF708 *was used as the scaffold (Table 6). The most dramatic difference was observed for the reductive charging phase of the metabolic cell cycle. While both models revealed the importance of regulation controlling the cellular response at glycogen, trehalose, UDP-glucose, glucose-6-P and glucose nodes, only *iIN800 *was able to identify key intermediates in β-oxidation. For example, *iIN800 *identified *trans*-3-acyl-CoAs, *trans*-2-acyl-CoAs, 3-keto-acyl-CoAs and some fatty acids as Reporter Metabolites (Table 6). This result demonstrates the advantage of expanding the metabolic model to include a much more detailed description of lipid metabolism. Namely, we can now use the genome-scale metabolic model to identify the regulatory importance of lipid precursors and intermediates at different physiological conditions or at different phases of cellular growth. Searching for highly co-regulated subnetworks that implicate lipid genes is also now possible.

Further demonstrations of the applicability of *iIN800 *as a scaffold to integrate omic data were performed by analyzing transcriptome data derived from nutrient-limited [36], oxygen-limited [36] and temperature stress conditions [37] Previously, mRNA and protein levels of genes and enzymes in fatty acid catabolism have been shown to be significantly different between carbon-limited and nitrogen limited growth [38]. When comparing these conditions, only *iIN800 *was able to identify fatty acids as Reporter Metabolites (Table 6). In anaerobic yeast cultivation, oleic acid has to be added to the medium because unsaturated fatty acids synthesis is not possible; therefore, the expression of genes in this pathway is induced by the function of the ORE element [39]. Consistent with this observed cellular response, only *iIN800*, with identified Reporter Metabolites involved in β-oxidation (Table 6). Similarly, *iIN800 *was able to highlight the importance of unsaturated fatty acids when comparing high and low temperature cultivations (Table 6), which is known to be important for maintaining proper membrane structure and fluidity [40].

**Figure 5:**
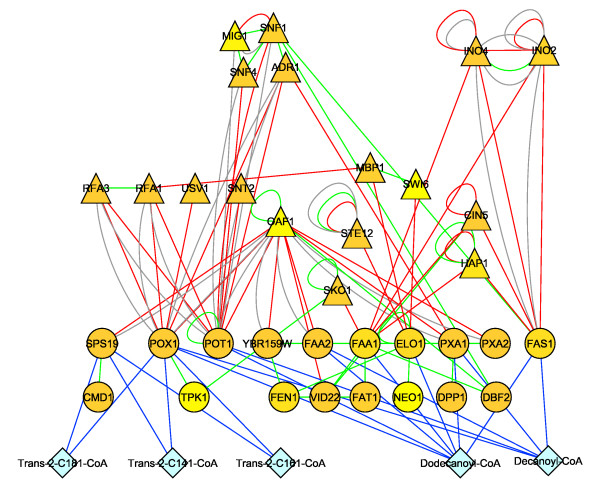
**Regulatory module implicated in the control of lipid metabolism genes associated to *iIN800 *Reporter Metabolites, which were determined by comparing N-limited and C-limited growth.** Without the expanded model *iIN800*, the importance of cellular regulation stemming from lipid metabolism would be missed. High scoring Reporter Metabolites (diamonds), metabolic genes associated to Reporter Metabolites (circles), and genes encoding regulators (triangles). The blue, red, gray and green edges represent metabolite-gene interactions from the genome-scale metabolic model, protein-DNA interactions from ChIP-CHIP data, protein-DNA interactions from YPD and protein-protein interactions from BioGRID, respectively.

Without the expanded model, the importance of cellular regulation stemming from lipid metabolism would be missed in analyses where metabolic topology is used for integrating data. As an illustration, we integrated results from our Reporter Metabolite analysis with known protein-protein and protein-DNA interaction networks to infer regulatory structure. First, genes associated to Reporter Metabolites in lipid metabolism unique to *iIN800 *and determined when comparing carbon- and nitrogen-limited growth (decanoyl-CoA, dodecanoyl-CoA, *trans*-2-C141-CoA, *trans*-2-C161-CoA, *trans*-2-C181-CoA) were identified. These genes were then used to search for highly regulated subnetworks within a protein-protein and protein-DNA interaction network. By applying a p-value threshold of 0.01 to filter for genes with significant gene expression, we inferred a regulatory network controlling the expression of lipid metabolism genes associated to the Reporter Metabolites (Figure 5). Strikingly, regulators at the top of this hierarchy are consistent with those previously known to be significantly changed between carbon- and nitrogen-limited growth. These include: *SNF1, SNF4, MIG1 *and *ADR1 *(glucose repression), *OAF1 *(β-oxidation), and *INO1 *and *INO4 *(phospholipid synthesis), among others. Previously reported genome-scale models are not capable of being used as scaffolds for implicating the conditional response of these lipid metabolism regulators because they lack a detailed description of lipid metabolism.

## Conclusion

Genome-scale metabolic models have emerged as a valuable tool in the post-genomic era for illustrating whole-cell functions based on the complete network of biochemical reactions. An iterative reconstruction process is required to achieve a comprehensive *S. cerevisiae *genome-scale metabolic model. In this work, we focused on improving the formulation of lipid metabolism relative to previously published *S. cerevisiae *genome-scale metabolic models. Validating the model and new biomass equations, the constraint-based simulation of *iIN800 *showed accurate predictions of cellular growth and is also consistent with ^13^C-labeling experiments. Furthermore, *in silico *gene essentialness predictions were found to be in high agreement with *in vivo *results. Finally, we show that *iIN800*, being more complete, is a better network scaffold for integration of multilevel omics data.

In conclusion, by incorporating a more complete description of lipid metabolism, *iIN800 *is positioned to have a broader impact than previously described yeast models. Its capability of predictions were consistent with a number of experimental data both quantitatively (growth rate) and qualitatively (gene essentialness). Moreover, the new model is positioned to be used for studying the regulation and role of lipid metabolism during different growth conditions. With the high degree of homology in lipid metabolism between yeast and humans and emergence of lipidomics, this is expected to allow for new insights into the connection between lipid metabolism and overall cellular function for industrial and medical applications.

## Methods

### Model reconstruction and visualization

Reconstruction of the *S. cerevisiae *genome-scale metabolic model was done by expanding *iFF708 *[16]. The additional ORFs included in the expansion procedure were involved in lipid metabolism, tRNA synthesis and lipoamide dehydrogenase. These ORFs were added based on publications listed in Additional file 1. Online resources related to *S. cerevisiae*, such as SGD [41], MIPS [42] and YPD [43], were also used to confirm the existence of the ORFs and their function. Pathway and reaction databases including KEGG [44], ExPASy [45], and Reactome [46], were used together with research papers to identify relevant information of the additional reactions and metabolites, e.g. stoichiometry and co-factor usage. The expanded *iFF708*, called *iIN800*, was visualized by Adobe Illustrator software (Adobe Systems), and then converted to EPS format (Adobe Systems) format which is downloadable as Additional file 6. In this visualization file, it is possible to overlay information about transcription, fluxes etc. A detailed list of metabolic reactions in *iIN800 *is provided as Additional file 7.

### Metabolic modeling and simulations

The reaction set in *iIN800 *was used for construction of a stoichiometric matrix ***S ***(m × n). In the stoichiometric matrix, m = 1013, which is the number of metabolites, and n = 1446, which is the number of metabolic reactions. With an assumption of steady state for all metabolite pools, a linear equation constraining the fluxes in the metabolic network is obtained [30,47]:

(1) ***S·v ***= **0**

Here ***v ***is a vector that contains all the fluxes in the model. Equation (1) has a large number of degrees of freedom, i.e. it is an underdetermined problem, and linear programming was employed to solve the equation system by maximizing an objective function Z (equal to the growth rate), an approach generally referred to as flux balance analysis (FBA) [30,47]. The problem formulation is described below.

Maximize:

***Z ***= ***ω·v***

Subject to:

***S·v ***= **0**

***α ***≤ ***v ***≤ ***β***

where ***α ***and ***β ***are lower and upper bounds of fluxes respectively, ***ω ***is a weight vector indicating an amount of desired metabolites for biomass synthesis. For irreversible fluxes semi-positive infinite boundary was applied as ***0 ***≤ ***v ***≤ ∞, and fully infinite boundaries was applied as -∞ ≤ ***v ***≤ ∞ for reversible fluxes. The problem was solved by using the commercial linear programming software package LINDO (Lindo systems Inc., Chicago, IL, USA). The calculated intracellular fluxes were overlaid on the visualized genome-scale map as described previously by the ReMapper software (The software has been developed for visualization of multilevel omics data onto a metabolic map.).

### Calculation of biomass composition and sensitivity analysis

The biomass composition was re-calculated in order to improve the prediction of the model during growth at different nutrition-limitations, i.e. carbon- and nitrogen-limited growth condition. The contents of macro-molecules were extracted from the thesis of Schulze [27] who measured the biomass composition at a dilution rate of 0.1 h^-1^. The calculations were performed as described previously [16]. The calculation of protein precursors, i.e. amino acids, and carbohydrate precursors, i.e. trehalose, glycogen, manna and glucan, were adopted from Schulze's work [27]. Deoxyribonucleotide and ribonucleotide compositions were calculated from the study of Vaughan-Martini and co-workers [48]. Lipid compositions were calculated from our own measurements of structural lipidomics, which contains phospholipids, triacylglycerol, sterols, sterol-esters, sphingolipids, free fatty acids and fatty acids composition of all measured lipid classes (unpublished data). The impact of the macromolecular composition on biomass yield was explored in aerobically glucose- and ammonium-limited conditions by fixing the specific growth rate and then minimizing the glucose and ammonium uptake rates at both glucose- and ammonium-limited growth conditions. Four parameters were evaluated, namely the protein, RNA, carbohydrate and lipid content of the biomass.

### Growth simulations

The metabolic capabilities of *iIN800 *were evaluated by using FBA and linear programming to simulate the biomass flux representing the *in silico *growth rate, which were derived by maximizing the biomass production. Data from various carbon-limited and nitrogen-limited chemostat experiments performed at either aerobic or anaerobic growth condition were taken from the literature for comparisons (see references in Additional file 3). These data were used to validate the metabolic capabilities of the model by comparing *in silico *biomass yields with *in vivo *biomass yields. The *in silico *biomass yields were calculated by fixing measurable uptake rates of extracellular metabolites, such as glucose, ammonium and oxygen, as well as secretions rates of acetate, glycerol, ethanol, succinate, pyruvate and carbon dioxide. The biomass equation (or flux), which was the objective function, was changed depending on the growth conditions evaluated according to the data provide in Table 4.

### Large-scale gene essentiality simulations

The impact of individual gene deletions on cell growth of *iIN800 *was evaluated by eliminating the reaction(s) corresponding to each gene in the model from the stoichiometric matrix ***S ***and then simulating growth of the mutant by FBA. The *in silico *gene essentialities were simulated for growth on rich- and minimal-medium. For minimal media, different carbon sources (glucose, galactose, glycerol and ethanol), ammonium, sulphate and phosphate were evaluated. For rich media, the uptake fluxes of amino acids, purines and pyrimidines were added as additional constraints as previously described [18]. The *in silico *simulations were compared to experimental data available in the MIPS and SGD databases and from competitive growth assays [34] as well as yeast mutant array experiments [22]. The power of *iIN800 *to predict gene essentiality was evaluated based on the criteria defined as follows:

Accuracy = (TP + TN)/(TP + TN + FP +FN)

Sensitivity = TP/(TP + FN)

Specificity = TN/(TN + FP)

Positive predictive value = TN/(TP + FP)

Negative predictive value = TN/(TN+FN)

Geometric mean = (Sensitivity·Specificity)^1/2^

where TP = true positive, TN = true negative, FP = false positive, FN = false negative. Positive and negative values referred to viable and lethal phenotype, respectively.

### Reporter Metabolite determination

Published microarray data were retrieved from Gene Expression Omnibus (GEO) [49]. The CEL files were normalized by the dChip software [50] in order to minimize overall intensity variation among a set of chips. The statistical test of significance was done by ANOVA or student t-test for p-value calculation.

Briefly, we describe the Reporter Metabolite calculations. The genome-scale model was converted to a bipartite undirected graph. In this graph each metabolite node has as neighbors the enzymes catalyzing the formation and consumption of the metabolite. The transcriptome data were mapped on the enzyme nodes using the significant values of gene expression. The normal commutative distribution was used to convert the p-values to a Z-score for further calculations. To identify an importance of metabolites in the metabolic network of the particular experimental conditions, the reporter algorithm was applied as described earlier [14].

### Inferring regulatory modules from Reporter Metabolites

The interactome network was initially constructed with data obtained from YPD [43], ChIP-chip databases [51] (protein-DNA interaction) and BioGRID [52] (protein-protein interaction). The candidate genes of high scoring Reporter Metabolites were retrieved from the bipartite metabolite-gene encoding enzyme interaction graph. They were then used to identify subnetworks from the interactome network [53]. Significantly changing p-values from microarray data were mapped on the subnetwork and then also genes having a p-value < 0.01 directly connected with the Reporter Metabolites. The module was visualized by Cytoscape software [54].

## Authors' contributions

IN designed the study, performed the metabolic reconstruction and validation, and contributed to manuscript writing. MCJ carried out the C^13^-labeling flux experiments, helped curate the model and contributed to manuscript writing. AM, CT, KL and SC contributed to the manuscript preparations, JN and SB participated in the concept and design of the study. All authors read and approved the final manuscript.

## Supplementary Material

Additional file 1 **Additional ORFs**. List if additional ORFs and their references containing in *iIN800*. Click here for file

Additional file 2 **Lipid metabolism reactions and comparison**. Comparison of lipid metabolism reactions of all *S. cerevisiae *genome-scale models. Click here for file

Additional file 3 **Growth simulation results**. Growth simulations and comparison with experimental measurements. Click here for file

Additional file 4 **Aerobic Flux distribution**. Visualization of flux distribution of aerobic growth mapping on Figure 1. Click here for file

Additional file 5 **Anaerobic Flux distribution**. Visualization of flux distribution of anaerobic growth mapping on Figure 1. Click here for file

Additional file 6 **High resolution file of Figure **1. High resolution of *S. cerevisiae *metabolic map is provided as EPS format. Click here for file

Additional file 7 ***iIN800 *model**. List of all participated reactions in *iIN800 *model. Click here for file
