# Supplementary material for: The genome-scale metabolic model iIN800 of Saccharomyces cerevisiae and its validation: a scaffold to query lipid metabolism
Source: BMC Syst Biol. 2008 Aug 7;2:71. doi: 10.1186/1752-0509-2-71 (PMC2542360; doi:10.1186/1752-0509-2-71)
Supplement: Additional file 1 — Additional ORFs. List if additional ORFs and their references containing in iIN800. [file 1752-0509-2-71-S1.pdf]

Table SI Additional ORFs and their references in iIN800

ORF	Name	References
Lipid metabolism		
YHR067W	HTD2	Kastaniotis AJ, et al. (2004) Mol Microbiol 53(5):1407-21
YBR026C	ETR1	Torkko JM, et al. (2001) Mol Cell Biol 21(18):6243-53
YJL196C	ELO1	Toke DA and Martin CE (1996) J Biol Chem 271(31):18413-22
YCR034W	ELO2	Oh CS, et al. (1997) J Biol Chem 272(28):17376-84
YLR372W	ELO3	Oh CS, et al. (1997) J Biol Chem 272(28):17376-84
YDL015C	TSC13	Kohlwein SD, et al. (2001) Mol Cell Biol 21(1):109-25
YBR159W	IFA38	Han G, et al. (2002) J Biol Chem 277(38):35440-9
YBR041W	FAT1	Zou Z, et al. (2002) J Biol Chem 277(34):31062-71
YNL202W	SPS19	Gurvitz A, et al. (1997) J Biol Chem 272(35):22140-7
YLR284C	ECI1	Gurvitz A, et al. (1998) J Biol Chem 273(47):31366-74
YOR180C	DCI1	Gurvitz A, et al. (1999) J Biol Chem 274(35):24514-21
YKR067W	GPT2	Zheng Z and Zou J (2001) J Biol Chem 276(45):41710-6
YBL011W	SCT1	Matsushita M and Nikawa J (1995) J Biochem (Tokyo) 117(2):447-51
YIL124W	AYR1	Athenstaedt K and Daum G (2000) J Biol Chem 275(1):235-40
YDR287W	YDR287W	Lopez F, et al. (1999) Mol Microbiol 31(4):1255-64
YFR019W	LSB6	Han GS, et al. (2002) J Biol Chem 277(49):47709-18
YHL003C	LAG1	D'mello NP, et al. (1994) J Biol Chem 269(22):15451-9
YKL008C	LAC1	Barz WP and Walter P (1999) Mol Biol Cell 10(4):1043-59
YCR048W	ARE1	Zweytick D, et al. (2000) Eur J Biochem 267(4):1075-82
YNR019W	ARE2	Zweytick D, et al. (2000) Eur J Biochem 267(4):1075-82
YML059C	NTE1	Murray JP and McMaster CR (2005) J Biol Chem 280(9):8544-52
YIL073C	SPO22	Tsubouchi T, et al. (2006) Dev Cell 10(6):809-19
YPL110C	GDE1	Fisher E, et al. (2005) J Biol Chem 280(43):36110-7
YKR031C	SPO14	Xie Z, et al. (1998) Proc Natl Acad Sci U S A 95(21):12346-51
YDL052C	ISC1	Sawai H, et al. (2000) J Biol Chem 275(50):39793-8
YMR008C	PLB1	Lee KS, et al. (1994) J Biol Chem 269(31):19725-30
YMR006C	PLB2	Fyrst H, et al. (1999) Biochemistry 38(18):5864-71
YOL011W	PLB3	Merkel O, et al. (1999) J Biol Chem 274(40):28121-7
YLL012W	YEH1	Koffel R, et al. (2005) Mol Cell Biol 25(5):1655-68
YLR020C	YEH2	Mullner H, et al. (2005) J Biol Chem 280(14):13321-8
YKL140W	TGL1	Koffel R, et al. (2005) Mol Cell Biol 25(5):1655-68

YOR245C	DGA1	Sorger D and Daum G (2002) J Bacteriol 184(2):519-24
YNR008W	LRO1	Oelkers P, et al. (2000) J Biol Chem 275(21):15609-12
YDR058C	TGL2	Van Heusden GP, et al. (1998) Yeast 14(3):225-32
YMR313C	TGL3	Athenstaedt K and Daum G (2003) J Biol Chem 278(26):23317-23
YKR089C	TGL4	Athenstaedt K and Daum G (2005) J Biol Chem 280(45):37301-9
YKR089C	TGL5	Athenstaedt K and Daum G (2005) J Biol Chem 280(45):37301-9
YOR109W	INP53	Guo S, et al. (1999) J Biol Chem 274(19):12990-5
YOL065C	INP54	Wiradjaja F, et al. (2001) J Biol Chem 276(10):7643-53
YNL106C	INP52	Guo S, et al. (1999) J Biol Chem 274(19):12990-5
YPL087W	YDC1	Mao C, et al. (2000) J Biol Chem 275(40):31369-78
YBR183W	YPC1	Mao C, et al. (2000) J Biol Chem 275(40):31369-78
YBR161W	CSH1	Uemura S, et al. (2003) J Biol Chem 278(46):45049-55
YCR098C	GIT1	Patton-Vogt JL and Henry SA (1998) Genetics 149(4):1707-15
YDR315C	IPK1	Ives EB, et al. (2000) J Biol Chem 275(47):36575-83
YJR019C	TES1	Jones JM, et al. (1999) J Biol Chem 274(14):9216-23
YER024W	YAT2	Swiegers JH, et al. (2001) Yeast 18(7):585-95
YJR110W	YMR1	Taylor GS, et al. (2000) Proc Natl Acad Sci U S A 97(16):8910-5
YKL212W	SAC1	Hughes WE, et al. (2000) J Biol Chem 275(2):801-8
YIL002C	INP51	Stolz LE, et al. (1998) J Biol Chem 273(19):11852-61
YNL325C	FIG4	Rudge SA, et al. (2004) Mol Biol Cell 15(1):24-36
tRNA synthesis		
YOR335C	ALA1	Wrobel C, et al. (1999) J Bacteriol 181(24):7618-20
YER087W		Sentandreu M, et al. (1997) Yeast 13(14):1375-81
YHR020W		Tatusov RL, et al. (2000) Nucleic Acids Res 28(1):33-6
YNL247W		Fleischer TC, et al. (2006) Genes Dev 20(10):1294-307
YOR168W	GLN4	Ludmerer SW, et al. (1993) J Biol Chem 268(8):5519-23
YDR023W	SES1	Weygand-Durasevic I, et al. (1987) Nucleic Acids Res 15(5):1887-904
YHR011W	DIA4	Yokogawa T, et al. (2000) J Biol Chem 275(26):19913-20
YBR121C	GRS1	Turner RJ, et al. (2000) J Biol Chem 275(36):27681-8
YPR081C	GRS2	Turner RJ, et al. (2000) J Biol Chem 275(36):27681-8
YGR171C	MSM1	Tzagoloff A, et al. (1989) Eur J Biochem 179(2):365-71
YGR264C	MES1	Chatton B, et al. (1987) J Biol Chem 262(31):15094-7
YKL194C	MST1	Pape LK, et al. (1985) J Biol Chem 260(28):15362-70
YIL078W	THS1	Pape LK and Tzagoloff A (1985) Nucleic Acids Res 13(17):6171-83

YPL097W	MSY1	Hill, J.E. and A.A. Tzagoloff (1995)
YGR185C	TYS1	Chow CM and RajBhandary UL (1993) J Biol Chem 268(17):12855-63
YOL097C	WRS1	John TR, et al. (1997) Yeast 13(1):37-41
YPR047W	MSF1	Koerner TJ, et al. (1987) J Biol Chem 262(8):3690-6
YFL022C	FRS2	Aphasizhev R, et al. (1996) Biochemistry 35(1):117-23
YLR060W	FRS1	Aphasizhev R, et al. (1996) Biochemistry 35(1):117-23
YGR094W	VAS1	Chatton B, et al. (1988) J Biol Chem 263(1):52-7
YLR382C	NAM2	Zagorski W, et al. (1991) J Biol Chem 266(4):2537-41
YPL160W	CDC60	Hohmann S and Thevelein JM (1992) Gene 120(1):43-9
YPL040C	ISM1	Tzagoloff A and Shtanko A (1995) Eur J Biochem 230(2):582-6
YBL076C	ILS1	Englisch U, et al. (1987) Biol Chem Hoppe Seyler 368(8):971-9
YPL104W	MSD1	Gampel A and Tzagoloff A (1989) Proc Natl Acad Sci U S A 86(16):6023-7
YLL018C	DPS1	Putz J, et al. (1991) Science 252(5013):1696-9
Lipoamide dehydrogenase		
YFL018C	LPD1	Ross J, et al. (1988) J Gen Microbiol 134(5):1131-9
Transporters		
YDL247W	MPH2	Day RE, et al. (2002) Yeast 19(12):1015-27
YJR160C	MPH3	Day RE, et al. (2002) Yeast 19(12):1015-27
YIL006W	YIA6	Todisco S, et al. (2006) J Biol Chem 281(3):1524-31
YEL006W	YEA6	Todisco S, et al. (2006) J Biol Chem 281(3):1524-31
YNR013C	PHO91	Wykoff DD and O'Shea EK (2001) Genetics 159(4):1491-9
YGL186C	TPN1	Stolz J and Vielreicher M (2003) Biol Chem 278(21):18990-6
YGR096W	TPC1	Marobbio CM, et al. (2002) EMBO J 21(21):5653-61
YCR010C	ADY2	Paiva S, et al. (2004) Yeast 21(3):201-10
YGR289C	MAL11	Cheng Q and Michels CA (1991) J Bacteriol 173(5):1817-20
YJL198W	PHO90	Auesukaree C, et al. (2003) Biochem Biophys Res Commun 306(4):843-50
YPR021C	AGC1	Cavero S, et al. (2003) Mol Microbiol 50(4):1257-69
YPL147W	PXA1	Hettema EH, et al. (1996) EMBO J 15(15):3813-22
YKL188C	PXA2	Hettema EH, et al. (1996) EMBO J 15(15):3813-22
YIL013C	PDR11	Wilcox LJ, et al. (2002) J Biol Chem 277(36):32466-72
