# Supplementary material for: The genome-scale metabolic model iIN800 of Saccharomyces cerevisiae and its validation: a scaffold to query lipid metabolism
Source: BMC Syst Biol. 2008 Aug 7;2:71. doi: 10.1186/1752-0509-2-71 (PMC2542360; doi:10.1186/1752-0509-2-71)
Supplement: Additional file 3 — Growth simulation results. Growth simulations and comparison with experimental measurements. [file 1752-0509-2-71-S3.pdf]

# Growth simulations and comparison with experimental measurements

## Experiment : C-limited Aerobic simulation

| $Y_{X/S}$ | $q_{glucose}$ | $q_{glycerol}$ | $q_{ethanol}$ | $q_{CO2}$ | $q_{O2}$ | $q_{succinate}$ | $q_{acetate}$ | $D_{in\ vivo}$ | $D_{in\ silico}$ | %error | Reference |
|---|---|---|---|---|---|---|---|---|---|---|---|
| 0.46 | 0.6 | 0 | 0 | 1.14 | *nr* | 0 | 0 | 0.0516 | 0.0545 | 5.62% | * |
| 0.49 | 1.15 | 0 | 0 | 2.80 | 2.70 | 0 | 0 | 0.1 | 0.11 | 10.00% | 1 |
| 0.48 | 1.17 | 0 | 0 | 2.50 | 2.50 | 0 | 0 | 0.1 | 0.11 | 10.00% | 2 |
| 0.52 | 1.18 | 0 | 0 | *nr* | *nr* | 0 | 0 | 0.11 | 0.106 | 3.64% | 3 |
| 0.5 | 1.69 | 0 | 0 | 4.10 | 4.00 | 0 | 0 | 0.15 | 0.16 | 6.67% | 2 |
| 0.5 | 2.26 | 0 | 0 | 5.20 | 5.00 | 0 | 0 | 0.2 | 0.22 | 10.00% | 2 |
| 0.49 | 2.88 | 0 | 0 | 6.60 | 6.50 | 0 | 0 | 0.25 | 0.25 | 0.00% | 2 |
| 0.46 | 3.27 | 0 | 0 | 8.37 | 7.46 | 0 | 0 | 0.27 | 0.29 | 7.41% | 5 |
| 0.48 | 3.29 | 0 | 0 | 8.10 | 7.80 | 0 | 0 | 0.28 | 0.3 | 7.14% | 2 |
| 0.45 | 3.88 | 0 | 1.00 | 9.50 | 8.00 | 0 | 0 | 0.31 | 0.29 | 6.45% | 2 |
| 0.3 | 6.20 | 0 | 5.00 | 12.00 | 7.00 | 0 | 0 | 0.33 | 0.34 | 3.03% | 2 |
| 0.25 | 7.89 | 0 | 6.00 | 15.00 | 6.50 | 0 | 0 | 0.35 | 0.36 | 2.86% | 2 |
| 0.16 | 13.39 | 0.65 | 18.00 | 22.00 | 3.00 | 0 | 0 | 0.38 | 0.37 | 2.63% | 2 |

## Experiment : C-limited Anaerobic simulation

| $Y_{X/S}$ | $q_{glucose}$ | $q_{glycerol}$ | $q_{ethanol}$ | $q_{CO2}$ | $q_{O2}$ | $q_{succinate}$ | $q_{acetate}$ | $D_{in\ vivo}$ | $D_{in\ silico}$ | %error | Reference |
|---|---|---|---|---|---|---|---|---|---|---|---|
| 0.07 | 2.3 | 0 | 3 | 3.8 | 0 | 0 | 0 | 0.03 | 0.029 | 3.33% | 6 |
| 0.11 | 2.86 | 0.26 | 3.74 | 4.78 | 0 | 0.02 | 0 | 0.047 | 0.042 | 10.64% | 4 |
| 0.1 | 5.88 | 1.01 | 8.77 | 9.60 | 0 | 0.03 | 0.04 | 0.101 | 0.1 | 0.99% | 4 |
| 0.097 | 11.31 | 2.06 | 16.84 | 18.53 | 0 | 0.05 | 0.10 | 0.19 | 0.198 | 4.21% | 4 |
| 0.096 | 16.75 | 3.18 | 24.82 | 26.83 | 0 | 0.05 | 0.30 | 0.281 | 0.291 | 3.56% | 4 |
| 0.094 | 22.18 | 4.83 | 33.07 | 34.73 | 0 | 0.07 | 0.67 | 0.369 | 0.342 | 7.32% | 4 |

*nr = not reported*

$Y_{X/S}$ (g/g) , $q_x$ (mmolgDW$^{-1}$hr$^{-1}$), $D_x$ (hr$^{-1}$)

## Experiment : N-limited Aerobic simulation

| $Y_{X/S}$ | $q_{glucose}$ | $q_{glycerol}$ | $q_{ethanol}$ | $q_{CO2}$ | $q_{O2}$ | $q_{succinate}$ | $q_{acetate}$ | $q_{NH3}$ | $D_{in\ vivo}$ | $D_{in\ silico}$ | %error | Reference |
|---|---|---|---|---|---|---|---|---|---|---|---|---|
| 0.09 | 5.8 | 0 | 8 | 12.1 | 2.7 | 0 | 0 | 0.40 | 0.1 | 0.105 | 5.00% | 6 |
| 0.11 | 4.83 | 0.04 | 6.56 | 12.11 | 4.42 | 0 | 0 | 0.42 | 0.1 | 0.105 | 5.00% | 5 |
| *nr* | 3.5 | 0 | 3.4 | *nr* | 7.8 | 0 | 0 | 0.61 | 0.15 | 0.143 | 4.67% | 8 |
| *nr* | 4.61 | 0 | 4.8 | *nr* | 9.2 | 0 | 0 | 0.74 | 0.18 | 0.169 | 6.11% | 8 |
| 0.22 | 5.3 | 0 | 5.14 | 10 | *nr* | 0.02 | 0 | 0.85 | 0.2 | 0.197 | 1.50% | 7 |
| *nr* | 5.67 | 0 | 5 | *nr* | 8.7 | 0 | 0 | 0.83 | 0.2 | 0.194 | 3.00% | 8 |
| *nr* | 8 | 0 | 12.1 | *nr* | 8.8 | 0 | 0 | 0.96 | 0.25 | 0.225 | 10.00% | 8 |
| *nr* | 9.45 | 0 | 14.9 | *nr* | 9.3 | 0 | 0 | 1.09 | 0.28 | 0.256 | 8.57% | 8 |
| *nr* | 12.68 | 0 | 21 | *nr* | 8.2 | 0 | 0 | 1.33 | 0.34 | 0.312 | 8.24% | 8 |

## Experiment : N-limited Anaerobic simulation

| $Y_{X/S}$ | $q_{glucose}$ | $q_{glycerol}$ | $q_{ethanol}$ | $q_{CO2}$ | $q_{O2}$ | $q_{succinate}$ | $q_{acetate}$ | $q_{NH3}$ | $D_{in\ vivo}$ | $D_{in\ silico}$ | %error | Reference |
|---|---|---|---|---|---|---|---|---|---|---|---|---|
| 0.027 | 4.15 | 0.025 | 6.23 | *nr* | 0 | 0.02 | 0.02 | 0.23 | 0.05 | 0.046 | 8.00% | 10 |
| 0.07 | 8.4 | 0 | 13.5 | 14.8 | 0 | 0 | 0 | 0.48 | 0.1 | 0.094 | 6.00% | 9 |
| 0.018 | 8.7 | 0.096 | 13.5 | *nr* | 0 | 0.044 | 0.08 | 0.55 | 0.1 | 0.087 | 13.00% | 10 |
| 0.02 | 12.4 | 0.15 | 18.9 | *nr* | 0 | 0.045 | 0.11 | 0.92 | 0.16 | 0.144 | 10.00% | 10 |
| 0.021 | 15.3 | 0.18 | 22.7 | *nr* | 0 | 0.06 | 0.17 | 1.33 | 0.2 | 0.194 | 3.00% | 10 |
| 0.022 | 17.4 | 0.22 | 26.05 | *nr* | 0 | 0.08 | 0.18 | 1.62 | 0.24 | 0.241 | 0.42% | 10 |

*nr = not reported*

$Y_{X/S}$ (g/g) , $q_x$ (mmolgDW$^{-1}$hr$^{-1}$), $D_x$ (hr$^{-1}$)


* Jewett et al., in preparation

1 Bakker, B. M., C. Bro, et al. (2000) J Bacteriol 182(17): 4730-7

2 Overkamp, K. M., B. M. Bakker, et al. (2000) J Bacteriol 182(10): 2823-30

3 Gombert, A. K., M. Moreira dos Santos, et al. (2001) J Bacteriol 183(4): 1441-51

4 Nissen, T. L., U. Schulze, et al. (1997) Microbiology 143 ( Pt 1): 203-18

5 Vemuri, G. N., M. A. Eiteman, et al. (2007) Proc Natl Acad Sci U S A 104(7): 2402-7

6 Tai, S. L., P. Daran-Lapujade, et al. (2007) J Biol Chem 282(14): 10243-51

7 Usaite, R., K. R. Patil, et al. (2006) Appl Environ Microbiol 72(9): 6194-203

8 Aon, J. C. and S. Cortassa (2001) Metab Eng 3(3): 250-64

9 Tai, S. L., V. M. Boer, et al. (2005) J Biol Chem 280(1): 437-47
10 Liden, G., A. Persson, et al. (1995) Appl Microbiol Biotechnol 43(6): 1034-8
