# Supplementary material for: The genome-scale metabolic model iIN800 of Saccharomyces cerevisiae and its validation: a scaffold to query lipid metabolism
Source: BMC Syst Biol. 2008 Aug 7;2:71. doi: 10.1186/1752-0509-2-71 (PMC2542360; doi:10.1186/1752-0509-2-71)
Supplement: Additional file 7 — iIN800 model. List of all participated reactions in iIN800 model. [file 1752-0509-2-71-S7.pdf]

iIN800 metabolic model

Reaction

AAC1	ADP + ATPM + Orthophosphate => H+M + ADPM + ATP + OrthophosphateM
AAC3	ADP + ATPM + Orthophosphate => H+M + ADPM + ATP + OrthophosphateM
AAH1_1	Adenosine => Inosine + NH3
AAH1_2	Deoxyadenosine => Deoxyinosine + NH3
AAH1_3	Adenine => NH3 + HYXN
AAT1_1	OxaloacetateM + GlutamateM <=> L=AspartateM + 2=OxoglutarateM
AAT1_2	3=(4=Hydroxyphenyl)pyruvate + L=Glutamate <=> 2=Oxoglutarate + L=Tyrosine
AAT2_1	Oxaloacetate + L=Glutamate <=> L=Aspartate + 2=Oxoglutarate
AAT2_2	3=(4=Hydroxyphenyl)pyruvate + L=Glutamate <=> 2=Oxoglutarate + L=Tyrosine
ABZ1	Chorismate + L=Glutamine => 4=amino=4=deoxychorismate + L=Glutamate
ACC1	Acetyl=CoA + ATP + CO2 <=> Malonyl=CoA + ADP + Orthophosphate
ACH1	Acetyl=CoA => CoA + Acetate
ACO1	CitrateM <=> IsocitrateM
ACP1	NADHM + Ubiquinone=9M => NAD+M + UbiquinolM
ACS1	ATP + Acetate + CoA => AMP + Pyrophosphate + Acetyl=CoA
ACS2	ATP + Acetate + CoA => AMP + Pyrophosphate + Acetyl=CoA
ADE1	1=(5=Phospho=D=ribosyl)=5=amino=4=imidazolecarboxylate + ATP + L=Aspartate <=> ADP + Orthophosphate + 1=(5'=Phosphoribosyl)=5=amino=4=(N=succinocarboxamide)=imidazole
ADE12	IMP + GTP + L=Aspartate => GDP + Orthophosphate + N6=(1,2=Dicarboxyethyl)=AMP
ADE13_1	1=(5'=Phosphoribosyl)=5=amino=4=(N=succinocarboxamide)=imidazole <=> Fumarate + 1=(5'=Phosphoribosyl)=5=amino=4=imidazolecarboxamide
ADE13_2	N6=(1,2=Dicarboxyethyl)=AMP <=> Fumarate + AMP
ADE16_1	1=(5'=Phosphoribosyl)=5=amino=4=imidazolecarboxamide + 10=Formyltetrahydrofolate <=> Tetrahydrofolate + 1=(5'=Phosphoribosyl)=5=formamido=4=imidazolecarboxamide
ADE16_2	1=(5'=Phosphoribosyl)=5=formamido=4=imidazolecarboxamide <=> IMP
ADE17_1	1=(5'=Phosphoribosyl)=5=amino=4=imidazolecarboxamide + 10=Formyltetrahydrofolate <=> Tetrahydrofolate + 1=(5'=Phosphoribosyl)=5=formamido=4=imidazolecarboxamide
ADE17_2	1=(5'=Phosphoribosyl)=5=formamido=4=imidazolecarboxamide <=> IMP
ADE2	1=(5=Phospho=D=ribosyl)=5=amino=4=imidazolecarboxylate <=> Aminoimidazole ribotide + CO2
ADE3_1	5,10=Methylenetetrahydrofolate + NADP+ <=> 5,10=Methenyltetrahydrofolate + NADPH
ADE3_2	Tetrahydrofolate + Formate + ATP => ADP + Orthophosphate + 10=Formyltetrahydrofolate
ADE3_3	5,10=Methenyltetrahydrofolate <=> 10=Formyltetrahydrofolate
ADE4	5=Phospho=alpha=D=ribose 1=diphosphate + L=Glutamine => Pyrophosphate + L=Glutamate + 5=Phosphoribosylamine

ADE5,7_1	5=Phosphoribosylamine + ATP + Glycine <=> ADP + Orthophosphate + 5'=Phosphoribosylglycinamide
ADE5,7_2	2=(Formamido)=N1=(5'=phosphoribosyl)acetamidine + ATP => ADP + Orthophosphate + Aminoimidazole ribotide
ADE6	5'=Phosphoribosyl=N=formylglycinamide + ATP + L=Glutamine => L=Glutamate + ADP + Orthophosphate + 2=(Formamido)=N1=(5'=phosphoribosyl)acetamidine
ADE8	5'=Phosphoribosylglycinamide + 10=Formyltetrahydrofolate => Tetrahydrofolate + 5'=Phosphoribosyl=N=formylglycinamide
ADH1	Ethanol + NAD+ <=> Acetaldehyde + NADH
ADH2	Ethanol + NAD+ <=> Acetaldehyde + NADH
ADH3	EthanolM + NAD+M <=> AcetaldehydeM + NADHM
ADH4	Ethanol + NAD+ <=> Acetaldehyde + NADH
ADH5	Ethanol + NAD+ <=> Acetaldehyde + NADH
ADK1_1	ATP + AMP <=> 2.000000 ADP
ADK1_2	GTP + AMP <=> ADP + GDP
ADK1_3	ITP + AMP <=> ADP + IDP
ADK2_1	ATPM + AMPM <=> 2.000000 ADPM
ADK2_2	GTPM + AMPM <=> ADPM + GDPM
ADK2_3	ITPM + AMPM <=> ADPM + IDPM
ADY2	ACxt + H+EXT <=> Acetate
AGC1_1	L=Glutamate <=> GlutamateM + H+M
AGC1_2	L=Aspartate <=> L=AspartateM + H+M
AGP1_1	GLUxt <=> L=Glutamate
AGP1_10	TYRxt + H+EXT <=> L=Tyrosine
AGP1_11	VALxt + H+EXT <=> L=Valine
AGP1_12	SERxt + H+EXT <=> L=Serine
AGP1_13	THRxt + H+EXT <=> L=Threonine
AGP1_2	ALAxt + H+EXT <=> L=Alanine
AGP1_3	ASNxt + H+EXT <=> L=Asparagine
AGP1_4	GLNxt + H+EXT <=> L=Glutamine
AGP1_5	HISxt + H+EXT <=> L=Histidine
AGP1_6	ILExt + H+EXT <=> L=Isoleucine
AGP1_7	LEUxt + H+EXT <=> L=Leucine
AGP1_8	METxt + H+EXT <=> L=Methionine
AGP1_9	PHExt + H+EXT <=> L=Phenylalanine
AGP2	CARxt <=> Carnitine
AGP3_1	GLUxt <=> L=Glutamate
AGP3_2	ASPxt + H+EXT <=> L=Aspartate

AGP3_3	SERxt + H+EXT <=> L=Serine
ALA1	ATP + L=Alanine + tRNA(Ala) => AMP + Pyrophosphate + L=Alaninyl=tRNA(Ala)
ALD2	Acetaldehyde + NAD+ => NADH + Acetate
ALD3	Acetaldehyde + NAD+ => NADH + Acetate
ALD4_1	AcetaldehydeM + NAD+M => NADHM + AcetateM
ALD4_2	AcetaldehydeM + NADP+M => NADPHM + AcetateM
ALD5_1	AcetaldehydeM + NADP+M => NADPHM + AcetateM
ALD5_2	(S)=LactaldehydeM + NAD+M <=> (S)=LactateM + NADHM
ALD6	Acetaldehyde + NADP+ => NADPH + Acetate
ALP1	ARGxt + H+EXT <=> L=Arginine
AMD1_1	AMP => IMP + NH3
AMD1_2	AMP => Adenine + D=Ribose 5=phosphate
AMD2_1	4=Guanidino=butanamide => 4=Guanidino=butanoate + NH3
AMD2_2	2=Phenylacetamide => Phenylacetic acid + NH3
AMD2_3	Indole=3=acetamide => Indole=3=acetate + NH3
APA1_1	ADP + GTP => Orthophosphate + P1,P4=Bis(5'=adenosyl) tetraphosphate
APA1_2	GDP + GTP => Orthophosphate + P1,P4=Bis(5'=guanosyl) tetraphosphate
APA1_3	ADP + Sulfate <=> Orthophosphate + Adenylylsulfate
APA2	ADP + ATP => Orthophosphate + P1,P4=Bis(5'=adenosyl) tetraphosphate
APT1	Adenine + 5=Phospho=alpha=D=ribose 1=diphosphate => Pyrophosphate + AMP
APT2	Adenine + 5=Phospho=alpha=D=ribose 1=diphosphate => Pyrophosphate + AMP
ARA1_1	D=Arabinose + NAD+ => D=Arabinono=1,4=lactone + NADH
ARA1_2	D=Arabinose + NADP+ => D=Arabinono=1,4=lactone + NADPH
ARE1	Ergosterol + AcylCoAs => Ergosterol=ester + CoA
ARE2	Ergosterol + AcylCoAs => Ergosterol=ester + CoA
ARG1	L=Citrulline + L=Aspartate + ATP <=> AMP + Pyrophosphate + N=(L=Arginino)succinate
ARG3	L=Ornithine + Carbamoyl phosphate => L=Citrulline + Orthophosphate
ARG4	N=(L=Arginino)succinate <=> Fumarate + L=Arginine
ARG5_1	N=Acetyl=L=glutamateM + ATPM => ADPM + N=Acetyl=L=glutamate 5=phosphateM
ARG5_2	N=Acetyl=L=glutamate 5=phosphateM + NADPHM => NADP+M + OrthophosphateM + N=Acetyl=L=glutamate 5=semialdehydeM
ARG8	N=Acetyl=L=glutamate 5=semialdehydeM + GlutamateM => 2=OxoglutarateM + N2=Acetyl=L=ornithineM
ARO1_1	2=Dehydro=3=deoxy=D=arabino=heptonate 7=phosphate => 3=Dehydroquinate + Orthophosphate
ARO1_2	3=Dehydroquinate => 3=Dehydroshikimate
ARO1_3	3=Dehydroshikimate + NADPH => Shikimate + NADP+

ARO1_4	Shikimate + ATP => ADP + Shikimate 3=phosphate
ARO1_5	Shikimate 3=phosphate + Phosphoenolpyruvate => 5=O=(1=Carboxyvinyl)=3=phosphoshikimate + Orthophosphate
ARO2	5=O=(1=Carboxyvinyl)=3=phosphoshikimate => Orthophosphate + Chorismate
ARO3	D=Erythrose 4=phosphate + Phosphoenolpyruvate => Orthophosphate + 2=Dehydro=3=deoxy=D=arabino=heptonate 7=phosphate
ARO4	D=Erythrose 4=phosphate + Phosphoenolpyruvate => Orthophosphate + 2=Dehydro=3=deoxy=D=arabino=heptonate 7=phosphate
ARO7	Chorismate => Prephenate
ARO8	3=(4=Hydroxyphenyl)pyruvate + L=Glutamate => 2=Oxoglutarate + L=Tyrosine
ARO9_1	Phenylpyruvate + L=Glutamate <=> 2=Oxoglutarate + L=Phenylalanine
ARO9_2	3=(4=Hydroxyphenyl)pyruvate + L=Glutamate => 2=Oxoglutarate + L=Tyrosine
ASN1	L=Aspartate + ATP + L=Glutamine => L=Glutamate + L=Asparagine + AMP + Pyrophosphate
ASN2	L=Aspartate + ATP + L=Glutamine => L=Glutamate + L=Asparagine + AMP + Pyrophosphate
ASP1	L=Asparagine => L=Aspartate + NH3
ASP3-1	L=Asparagine => L=Aspartate + NH3
ASP3-2	L=Asparagine => L=Aspartate + NH3
ASP3-3	L=Asparagine => L=Aspartate + NH3
ASP3-4	L=Asparagine => L=Aspartate + NH3
ATH1	alpha,alpha=Trehalose => 2.000000 alpha=D=Glucose
ATP1	ADPM + OrthophosphateM => ATPM + 3.000000 H+M
AUR1_1	D=Ceramide + 1=Phosphatidyl=D=myo=inositol => Inositol=phosphoryl=D=ceramide
AUR1_2	P=Ceramide + 1=Phosphatidyl=D=myo=inositol => Inositol=phosphoryl=P=ceramide
AUS1_1	ERGOSTxt <=> Ergosterol
AUS1_2	ZYMSTxt <=> Zymosterol
AUS1_3	LANOSTxt <=> Lanosterol
AUS1_4	44DIMZYMSTxt <=> 4,4=Dimethylzymosterol
AUS1_5	FCOSTxt <=> Fecosterol
AUS1_6	EPISTxt <=> Episterol
AUS1_7	ERG722xt <=> Ergosta=7,22=diene=ol
AUS1_8	ERG5,7,22,24xt <=> Ergosta=5,7,22,24(28)=tetraenol
AYR1	Acyldihydroxyacetone phosphate + NADPH => Acyl=sn=glycerol 3=phosphate + NADPH
BAP2_1	CYSxt + H+EXT <=> L=Cysteine
BAP2_2	ILExt + H+EXT <=> L=Isoleucine
BAP2_3	LEUxt + H+EXT <=> L=Leucine
BAP2_4	METxt + H+EXT <=> L=Methionine
BAP2_5	PHExt + H+EXT <=> L=Phenylalanine

BAP2_6	TRPxt + H+EXT <=> L=Tryptophan
BAP2_7	TYRxt + H+EXT <=> L=Tyrosine
BAP2_8	VALxt + H+EXT <=> L=Valine
BAP3_1	CYSxt + H+EXT <=> L=Cysteine
BAP3_2	ILExt + H+EXT <=> L=Isoleucine
BAP3_3	LEUxt + H+EXT <=> L=Leucine
BAP3_4	METxt + H+EXT <=> L=Methionine
BAP3_5	PHExt + H+EXT <=> L=Phenylalanine
BAP3_6	TRPxt + H+EXT <=> L=Tryptophan
BAP3_7	TYRxt + H+EXT <=> L=Tyrosine
BAP3_8	VALxt + H+EXT <=> L=Valine
BAT1_1	3=Carboxy=4=methyl=2=oxopentanoateM + GlutamateM <=> 2=OxoglutarateM + L=LeucineM
BAT1_2	3=Methyl=2=oxobutanoateM + GlutamateM <=> 2=OxoglutarateM + L=IsoleucineM
BAT2_1	3=Methyl=2=oxobutanoate + L=Glutamate <=> 2=Oxoglutarate + L=Isoleucine
BAT2_2	(R)=2=Oxoisovalerate + L=Glutamate <=> 2=Oxoglutarate + L=Valine
BAT2_3	3=Carboxy=4=methyl=2=oxopentanoate + L=Glutamate <=> 2=Oxoglutarate + L=Leucine
BET2	4=Hydroxybenzoate + all=trans=Nonaprenyl diphosphate => 3=Nonaprenyl=4=hydroxybenzoate + Pyrophosphate
BGL2	1,3=beta=D=Glucan => alpha=D=Glucose
BIO2	Dethiobiotin + L=Cysteine <=> Biotin
BIO3	S=Adenosyl=L=methionine + 8=Amino=7=oxononanoate <=> S=Adenosyl=4=methylthio=2=oxobutanoate + 7,8=Diaminononanoate
BIO4	CO2 + 7,8=Diaminononanoate + ATP <=> Dethiobiotin + Orthophosphate + ADP
BIO5	BIOxt + H+EXT => Biotin
BNA1	3=Hydroxyanthranilate + Oxygen => 2=Amino=3=carboxymuconate semialdehyde
BPH1	ACxt + H+EXT <=> Acetate
CAN1_1	ARGxt + H+EXT <=> L=Arginine
CAN1_2	ORNxt + H+EXT <=> L=Ornithine
CAR1	L=Arginine => L=Ornithine + Urea
CAR2	L=Ornithine + 2=Oxoglutarate => L=Glutamate 5=semialdehyde + L=Glutamate
CAT2	Acetyl=CoA + Carnitine => CoA + O=Acetylcarnitine
CDA1	Chitin => Chitosan + Acetate
CDA2	Chitin => Chitosan + Acetate
CDC19	Phosphoenolpyruvate + ADP => Pyruvate + ATP
CDC21	dUMP + 5,10=Methylenetetrahydrofolate => Dihydrofolate + dTMP
CDC60	ATP + L=Leucine + tRNA(Leu) => AMP + Pyrophosphate + L=Leucyl=tRNA(Leu)

CDC8	dTMP + ATP <=> ADP + dTDP
CDD1_1	Cytidine => Uridine + NH3
CDD1_2	Deoxycytidine => NH3 + Deoxyuridine
CDS1_1	PhosphatidateM + CTPM <=> CDPdiacylglycerolM + PyrophosphateM
CDS1_2	Phosphatidate + CTP <=> CDPdiacylglycerol + Pyrophosphate
CEM1_1	Acetyl=[acyl=carrier protein]M + Malonyl=[acyl=carrier protein]M => Acetoacetyl=[acyl=carrier protein]M + Acyl=carrier proteinM + CO2M
CEM1_2	Butyryl=ACPM + Malonyl=[acyl=carrier protein]M => 3=Oxo=Hexanoyl=ACPM + Acyl=carrier proteinM + CO2M
CEM1_3	Hexanoyl=ACPM + Malonyl=[acyl=carrier protein]M => 3=Oxo=Octanoyl=ACPM + Acyl=carrier proteinM + CO2M
CEM1_4	Octanoyl=ACPM + Malonyl=[acyl=carrier protein]M => 3=Oxo=Decanoyl=ACPM + Acyl=carrier proteinM + CO2M
CEM1_5	Decanoyl=ACPM + Malonyl=[acyl=carrier protein]M => 3=Oxo=Dodecanoyl=ACPM + Acyl=carrier proteinM + CO2M
CEM1_6	Dodecanoyl=ACPM + Malonyl=[acyl=carrier protein]M => 3=Oxo=Tetradecanoyl=ACPM + Acyl=carrier proteinM + CO2M
CEM1_7	Tetradecanoyl=ACPM + Malonyl=[acyl=carrier protein]M => 3=Oxo=Hexadecanoyl=ACPM + Acyl=carrier proteinM + CO2M
CEM1_8	Hexadecanoyl=ACPM + Malonyl=[acyl=carrier protein]M => 3=Oxo=Octadecanoyl=ACPM + Acyl=carrier proteinM + CO2M
CHA1_1	L=Threonine => NH3 + 2=Oxobutanoate
CHA1_2	L=Serine => Pyruvate + NH3
CHO1_1	CDPdiacylglycerol + L=Serine <=> CMP + Phosphatidylserine
CHO1_2	CDPdiacylglycerolM + L=SerineM <=> CMPM + PhosphatidylserineM
CHO2	S=Adenosyl=L=methionine + Phosphatidylethanolamine => S=Adenosyl=L=homocysteine + Phosphatidyl=N=methylethanolamine
CHS1	UDP=N=acetyl=D=galactosamine => Chitin + UDP
CHS2	UDP=N=acetyl=D=galactosamine => Chitin + UDP
CHS3	UDP=N=acetyl=D=galactosamine => Chitin + UDP
CIT1	Acetyl=CoAM + OxaloacetateM => CoAM + CitrateM
CIT2	Acetyl=CoA + Oxaloacetate => CoA + Citrate
CIT3	Acetyl=CoAM + OxaloacetateM => CoAM + CitrateM
CKI1	ATP + Choline => ADP + Choline phosphate
COQ1	4=Hydroxybenzoate + all=trans=Nonaprenyl diphosphate => 3=Nonaprenyl=4=hydroxybenzoate + Pyrophosphate
COQ2	4=Hydroxybenzoate + all=trans=Nonaprenyl diphosphate => 3=Nonaprenyl=4=hydroxybenzoate + Pyrophosphate
COQ3	3=Demethylubiquinone=9M + S=Adenosyl=L=methionineM => UbiquinolM + S=Adenosyl=L=homocysteineM
COQ5	2=Nonaprenyl=6=methoxy=1,4=benzoquinoneM + S=Adenosyl=L=methionineM => 2=Nonaprenyl=3=methyl=6=methoxy=1,4=benzoquinoneM + S=Adenosyl=L=homocysteineM
COQ6	2=Nonaprenyl=3=methyl=6=methoxy=1,4=benzoquinoneM + OxygenM => 3=Demethylubiquinone=9M
COX1	4.000000 Ferrocytochrome cM + OxygenM + 6.000000 H+M => 4.000000 Ferricytochrome cM
COX10	4=Hydroxybenzoate + all=trans=Nonaprenyl diphosphate => 3=Nonaprenyl=4=hydroxybenzoate + Pyrophosphate
CPA2	L=Glutamine + 2.000000 ATP + CO2 => L=Glutamate + Carbamoyl phosphate + 2.000000 ADP + Orthophosphate
CPT1	CDPcholine + Diacylglycerol => Phosphatidylcholine + CMP

CRC1	CarnitineM + O=Acetylcarnitine => Carnitine + O=AcetylcarnitineM
CRD1	CDPdiacylglycerolM + PhosphatidylglycerolM => CMPM + CardiolipinM
CSG2_1	Inositol=phosphoryl=D=ceramide + GDPmannose => Mannose=inositol=P=D=ceramide
CSG2_2	Inositol=phosphoryl=P=ceramide + GDPmannose => Mannose=inositol=P=P=ceramide
CTA1	2.000000 H2O2 => Oxygen
CTP1_1	Citrate + MalateM <=> CitrateM + Malate
CTP1_2	Citrate + PhosphoenolpyruvateM <=> CitrateM + Phosphoenolpyruvate
CTP1_3	Citrate + IsocitrateM <=> CitrateM + Isocitrate
CTT1	2.000000 H2O2 => Oxygen
CYB2	2.000000 Ferricytochrome cM + (S)=LactateM => PyruvateM + 2.000000 Ferrocytochrome cM
CYR1	ATP => 3',5'=Cyclic AMP + Pyrophosphate
CYS3	L=Cystathionine => L=Cysteine + NH3 + 2=Oxobutanoate
CYS4	L=Serine + Homocysteine => L=Cystathionine
DAK1	Glycerone + ATP => Glycerone phosphate + ADP
DAK2	Glycerone + ATP => Glycerone phosphate + ADP
DAL1	Allantoin <=> Allantoate
DAL2	Allantoate <=> (=)=Ureidoglycolate + Urea
DAL3	(=)=Ureidoglycolate <=> Glyoxylate + 2.000000 NH3 + CO2
DAL4	ATNxt => Allantoin
DAL5	ATTxt => Allantoate
DAL7	Acetyl=CoA + Glyoxylate => CoA + Malate
DCD1_1	dCMP <=> dUMP + NH3
DCD1_2	dCTP => dUTP + NH3
DCI1_1	Trans=3=C16=CoA => Trans=2=C16=CoA
DCI1_2	Trans=3=C18=CoA => Trans=2=C18=CoA
DCI1_3	Trans=3=C14=CoA => Trans=2=C14=CoA
DCI1_4	Trans=3=5=diene=CoA => Trans=2=4=diene=CoA
DED81	ATP + L=Asparagine + tRNA => AMP + Pyrophosphate + L=Asparaginyl=tRNA(Asn)
DEG1	Uracil + D=Ribose 5=phosphate <=> Pseudouridine 5'=phosphate
DFR1_1	DihydrofolateM + NADPHM => NADP+M + TetrahydrofolateM
DFR1_2	Dihydrofolate + NADPH => NADP+ + Tetrahydrofolate
DGA1	AcylCoAs + Diacylglycerol => Triacylglycerol + CoA
DIA1	ATP + L=Serine + tRNA(Ser) => AMP + Pyrophosphate + L=Serinyl=tRNA(Ser)
DIC1_1	Malate + SuccinateM <=> MalateM + Succinate

DIC1_2	Malate + OrthophosphateM <=> MalateM + Orthophosphate
DIC1_3	Succinate + OrthophosphateM => SuccinateM + Orthophosphate
DIM1	2=Nonaprenyl=6=hydroxyphenol + S=Adenosyl=L=methionine => 2=Nonaprenyl=6=methoxyphenol + S=Adenosyl=L=homocysteine
DIP5_1	GLUxt <=> L=Glutamate
DIP5_2	ALAxt + H+EXT <=> L=Alanine
DIP5_3	ASNxt + H+EXT <=> L=Asparagine
DIP5_4	ASPxt + H+EXT <=> L=Aspartate
DIP5_5	GLYxt + H+EXT <=> Glycine
DIP5_6	GLNxt + H+EXT <=> L=Glutamine
DIP5_7	SERxt + H+EXT <=> L=Serine
DLD1	2.000000 Ferricytochrome cM + (R)=LactateM => PyruvateM + 2.000000 Ferrocytochrome cM
DPH5	S=Adenosyl=L=methionine + 2=(3=Carboxy=3=aminopropyl)=L=histidine => S=Adenosyl=L=homocysteine + 2=[3=Carboxy=3=(methylammonio)propyl]=L=histidine
DPL1_1	Sphinganine 1=phosphate => Ethanolamine phosphate + C16_aldehydes
DPL1_2	Phytosphingosine 1=phosphate => Ethanolamine phosphate + C16_aldehydes
DPM1	GDPmannose + Dolichyl phosphate => GDP + Dolichyl beta=D=mannosyl phosphate
DPP1	Phosphatidate => Diacylglycerol + Pyrophosphate
DPS1	ATP + L=Aspartate + tRNA(Asp) => AMP + Pyrophosphate + L=Aspartyl=tRNA(Asp)
DUR1_1	ATP + Urea + CO2 <=> ADP + Orthophosphate + Urea=1=carboxylate
DUR1_2	Urea=1=carboxylate => 2.000000 NH3 + 2.000000 CO2
DUR3	UREAxt + 2.000000 H+EXT <=> Urea
DUT1	dUTP => Pyrophosphate + dUMP
DYS1	Spermidine + Ubiquinone=9M => 1,3=Diaminopropane + UbiquinolM
ECI1_1	Trans=3=C16=CoA => Trans=2=C16=CoA
ECI1_2	Trans=3=C18=CoA => Trans=2=C18=CoA
ECI1_3	Trans=3=C14=CoA => Trans=2=C14=CoA
ECM17	Sulfite + 3.000000 NADPH <=> Hydrogen sulfide + 3.000000 NADP+
ECM31	(R)=2=Oxoisovalerate + 5,10=Methylenetetrahydrofolate => 2=Dehydropantoate + Tetrahydrofolate
ECM38	Glutathione + L=Alanine => Cys=Gly + R=S=Alanylglycine
ECM40_1	GlutamateM + Acetyl=CoAM => CoAM + N=Acetyl=L=glutamateM
ECM40_2	N2=Acetyl=L=ornithineM + GlutamateM => L=OrnithineM + N=Acetyl=L=glutamateM
EKI1	ATP + Ethanolamine => ADP + Ethanolamine phosphate
ELO1_1	Dodecanoyl=CoA + Malonyl=CoA => 3=Keto=C14=CoA + CoA
ELO1_2	Tetradecanoyl=CoA + Malonyl=CoA => 3=Keto=C16=CoA + CoA
ELO2_3	Hexadecanoyl=CoA + Malonyl=CoA => 3=Keto=C18=CoA + CoA

ELO2_4	Octadecanoyl=CoA + Malonyl=CoA => 3=Keto=C20=CoA + CoA
ELO2_5	C20=CoA + Malonyl=CoA => 3=Keto=C22=CoA + CoA
ELO2_6	C22=CoA + Malonyl=CoA => 3=Keto=C24=CoA + CoA
ELO2_7	C24=CoA + Malonyl=CoA => 3=Keto=C26=CoA + CoA
ELO3_3	Hexadecanoyl=CoA + Malonyl=CoA => 3=Keto=C18=CoA + CoA
ELO3_4	Octadecanoyl=CoA + Malonyl=CoA => 3=Keto=C20=CoA + CoA
ELO3_5	C20=CoA + Malonyl=CoA => 3=Keto=C22=CoA + CoA
ELO3_6	C22=CoA + Malonyl=CoA => 3=Keto=C24=CoA + CoA
ELO3_7	C24=CoA + Malonyl=CoA => 3=Keto=C26=CoA + CoA
ENO1	2=Phospho=D=glycerate <=> Phosphoenolpyruvate
ENO2	2=Phospho=D=glycerate <=> Phosphoenolpyruvate
EPT1	CDPethanolamine + Diacylglycerol <=> CMP + Phosphatidylethanolamine
ERG1	Squalene + Oxygen + NADP+ => (S)=2,3=Epoxysqualene + NADPH
ERG10	2.000000 Acetyl=CoA <=> CoA + Acetoacetyl=CoA
ERG11	Lanosterol + Reduced flavoprotein + Oxygen => 4,4=Dimethylcholesta=8,14,24=trienol + Oxidized flavoprotein
ERG12_1	ATP + (R)=Mevalonate => ADP + (R)=5=Phosphomevalonate
ERG12_2	CTP + (R)=Mevalonate => CDP + (R)=5=Phosphomevalonate
ERG12_3	GTP + (R)=Mevalonate => GDP + (R)=5=Phosphomevalonate
ERG12_4	UTP + (R)=Mevalonate => UDP + (R)=5=Phosphomevalonate
ERG2	Fecosterol => Episterol
ERG20_1	Dimethylallyl diphosphate + Isopentenyl diphosphate => Geranyl diphosphate + Pyrophosphate
ERG20_2	Geranyl diphosphate + Isopentenyl diphosphate => trans,trans=Farnesyl diphosphate + Pyrophosphate
ERG24	4,4=Dimethylcholesta=8,14,24=trienol + NADPH => 4,4=Dimethylzymosterol + NADP+
ERG25_1	3.000000 Oxygen + 4,4=Dimethylzymosterol => Intermediate_Methylzymosterol_I
ERG25_2	3.000000 Oxygen + 4=Methylzymsterol => Intermediate_Zymosterol_I
ERG26_1	Intermediate_Methylzymosterol_I => Intermediate_Methylzymosterol_II + CO2
ERG26_2	Intermediate_Zymosterol_I => Intermediate_Zymosterol_II + CO2
ERG27_1	Intermediate_Methylzymosterol_II + NADPH => 4=Methylzymsterol + NADP+
ERG27_2	Intermediate_Zymosterol_II + NADPH => Zymosterol + NADP+
ERG3	Episterol + Oxygen + NADPH => NADP+ + Ergosta=5,7,24(28)=trienol
ERG4	Ergosta=5,7,22,24(28)=tetraenol + NADPH => Ergosterol + NADP+
ERG5	Ergosta=5,7,24(28)=trienol + Oxygen + NADPH => NADP+ + Ergosta=5,7,22,24(28)=tetraenol
ERG6	Zymosterol + S=Adenosyl=L=methionine => Fecosterol + S=Adenosyl=L=homocysteine
ERG7	(S)=2,3=Epoxysqualene => Lanosterol

ERG8	ATP + (R)=5=Phosphomevalonate => ADP + (R)=5=Diphosphomevalonate
ERG9_1	2.000000 trans,trans=Farnesyl diphosphate => Pre=Squalene=PP
ERG9_2	Pre=Squalene=PP + NADPH => NADP+ + Squalene
ERR1_1	2=Phospho=D=glycerate <=> Phosphoenolpyruvate
ERR1_2	2=Phospho=D=glycerate <=> Phosphoenolpyruvate
ERR2	2=Phospho=D=glycerate <=> Phosphoenolpyruvate
ETR1_1	trans=But=2=enoyl=ACPM + NADPHM <=> Butyryl=ACP + NADP+M
ETR1_2	trans=Hex=2=enoyl=ACPM + NADPHM <=> Hexanoyl=ACPM + NADP+M
ETR1_3	trans=Oct=2=enoyl=ACPM + NADPHM <=> Octanoyl=ACPM + NADP+M
ETR1_4	trans=Dec=2=enoyl=ACPM + NADPHM <=> Decanoyl=ACPM + NADP+M
ETR1_5	trans=Dodec=2=enoyl=ACPM + NADPHM <=> Dodecanoyl=ACPM + NADP+M
ETR1_6	trans=Tetradec=2=enoyl=ACPM + NADPHM <=> Tetradecanoyl=ACPM + NADP+M
ETR1_7	trans=Hexadec=2=enoyl=ACPM + NADPHM <=> Hexadecanoyl=ACPM + NADP+M
ETR1_8	trans=Octadec=2=enoyl=ACPM + NADPHM <=> Octadecanoyl=ACPM + NADP+M
EXG1	1,3=beta=D=Glucan => alpha=D=Glucose
EXG2	1,3=beta=D=Glucan => alpha=D=Glucose
FAA1_1	ATP + Dodecanoic_acid + CoA => AMP + Pyrophosphate + Dodecanoyl=CoA
FAA1_2	ATP + Tetradecanoic_acid + CoA => AMP + Pyrophosphate + Tetradecanoyl=CoA
FAA1_3	ATP + Hexadecanoic_acid + CoA => AMP + Pyrophosphate + Hexadecanoyl=CoA
FAA2_1	ATP + Decanoic_acid + CoA => AMP + Pyrophosphate + Decanoyl=CoA
FAA2_2	ATP + Dodecanoic_acid + CoA => AMP + Pyrophosphate + Dodecanoyl=CoA
FAA3_1	ATP + Hexadecanoic_acid + CoA => AMP + Pyrophosphate + Hexadecanoyl=CoA
FAA3_2	ATP + Octadecanoic_acid + CoA => AMP + Pyrophosphate + Octadecanoyl=CoA
FAA4_1	ATP + Hexadecanoic_acid + CoA => AMP + Pyrophosphate + Hexadecanoyl=CoA
FAA4_2	ATP + Octadecanoic_acid + CoA => AMP + Pyrophosphate + Octadecanoyl=CoA
FAA4_3	ATP + Hexadecanoyl=9=ene_acid + CoA => AMP + Pyrophosphate + Hexadecanoyl=9=ene=CoA
FAA4_4	ATP + Octadecanoyl=9=ene_acid + CoA => AMP + Pyrophosphate + Octadecanoyl=9=ene=CoA
FAA4_5	ATP + Tetradecanoyl=9=ene_acid + CoA => AMP + Pyrophosphate + Tetradecanoyl=9=ene=CoA
FAB1	1=Phosphatidyl=D=myo=inositol=3=phosphate + ATP => 1=Phosphatidyl=D=myo=inositol=3,5=bisphosphate + ADP
FAD1	FMN + ATP => FAD + Pyrophosphate
FAS1_1	Malonyl=CoA + Acyl=carrier protein <=> Malonyl=[acyl=carrier protein] + CoA
FAS1_1_1	R=3=Hydroxylbutanoyl=ACP => trans=But=2=enoyl=ACP
FAS1_1_2	trans=But=2=enoyl=ACP + NADPH <=> Butyryl=ACP + NADP+
FAS1_2_1	R=3=Hydroxylhexanoyl=ACP => trans=Hex=2=enoyl=ACP

FAS1_2_2	trans=Hex=2=enoyl=ACP + NADPH <=> Hexanoyl=ACP + NADP+
FAS1_3_1	R=3=Hydroxyloctanoyl=ACP => trans=Oct=2=enoyl=ACP
FAS1_3_2	trans=Oct=2=enoyl=ACP + NADPH <=> Octanoyl=ACP + NADP+
FAS1_4_1	R=3=Hydroxyldecanoyl=ACP => trans=Dec=2=enoyl=ACP
FAS1_4_2	trans=Dec=2=enoyl=ACP + NADPH <=> Decanoyl=ACP + NADP+
FAS1_4_c	Decanoyl=ACP + CoA <=> Decanoyl=CoA + Acyl=carrier protein
FAS1_4_f	Decanoyl=ACP => Decanoic_acid + Acyl=carrier protein
FAS1_5_1	R=3=Hydroxyldodecanoyl=ACP => trans=Dodec=2=enoyl=ACP
FAS1_5_2	trans=Dodec=2=enoyl=ACP + NADPH <=> Dodecanoyl=ACP + NADP+
FAS1_5_c	Dodecanoyl=ACP + CoA <=> Dodecanoyl=CoA + Acyl=carrier protein
FAS1_5_f	Dodecanoyl=ACP => Dodecanoic_acid + Acyl=carrier protein
FAS1_6_1	R=3=Hydroxyltetradecanoyl=ACP => trans=Tetradec=2=enoyl=ACP
FAS1_6_2	trans=Tetradec=2=enoyl=ACP + NADPH <=> Tetradecanoyl=ACP + NADP+
FAS1_6_c	Tetradecanoyl=ACP + CoA <=> Tetradecanoyl=CoA + Acyl=carrier protein
FAS1_6_f	Tetradecanoyl=ACP => Tetradecanoic_acid + Acyl=carrier protein
FAS1_7_1	R=3=Hydroxylhe 3=Keto=C22=CoA + NADPH => 3=Hydroxy=C22=CoA + NADP+
FAS1_7_2	trans=Hexadec=2=enoyl=ACP + NADPH <=> Hexadecanoyl=ACP + NADP+
FAS1_7_c	Hexadecanoyl=ACP + CoA <=> Hexadecanoyl=CoA + Acyl=carrier protein
FAS1_7_f	Hexadecanoyl=ACP => Hexadecanoic_acid + Acyl=carrier protein
FAS1_8_1	R=3=Hydroxyloctadecanoyl=ACP => trans=Octadec=2=enoyl=ACP
FAS1_8_2	trans=Octadec=2=enoyl=ACP + NADPH <=> Octadecanoyl=ACP + NADP+
FAS1_8_c	Octadecanoyl=ACP + CoA <=> Octadecanoyl=CoA + Acyl=carrier protein
FAS1_8_f	Octadecanoyl=ACP => Octadecanoic_acid + Acyl=carrier protein
FAS1_s	Acetyl=CoA + Acyl=carrier protein <=> Acetyl=[acyl=carrier protein] + CoA
FAS2_1_1	Acetyl=[acyl=carrier protein] + Malonyl=[acyl=carrier protein] => Acetoacetyl=[acyl=carrier protein] + Acyl=carrier protein + CO2
FAS2_1_2	Acetoacetyl=[acyl=carrier protein] + NADPH <=> R=3=Hydroxylbutanoyl=ACP + NADP+
FAS2_2_1	Butyryl=ACP + Malonyl=[acyl=carrier protein] => 3=Oxo=Hexanoyl=ACP + Acyl=carrier protein + CO2
FAS2_2_2	3=Oxo=Hexanoyl=ACP + NADPH <=> R=3=Hydroxylhexanoyl=ACP + NADP+
FAS2_3_1	Hexanoyl=ACP + Malonyl=[acyl=carrier protein] => 3=Oxo=Octanoyl=ACP + Acyl=carrier protein + CO2
FAS2_3_2	3=Oxo=Octanoyl=ACP + NADPH <=> R=3=Hydroxyloctanoyl=ACP + NADP+
FAS2_4_1	Octanoyl=ACP + Malonyl=[acyl=carrier protein] => 3=Oxo=Decanoyl=ACP + Acyl=carrier protein + CO2
FAS2_4_2	3=Oxo=Decanoyl=ACP + NADPH <=> R=3=Hydroxyldecanoyl=ACP + NADP+
FAS2_5_1	Decanoyl=ACP + Malonyl=[acyl=carrier protein] => 3=Oxo=Dodecanoyl=ACP + Acyl=carrier protein + CO2
FAS2_5_2	3=Oxo=Dodecanoyl=ACP + NADPH <=> R=3=Hydroxyldodecanoyl=ACP + NADP+

FAS2_6_1	Dodecanoyl=ACP + Malonyl=[acyl=carrier protein] => 3=Oxo=Tetradecanoyl=ACP + Acyl=carrier protein + CO2
FAS2_6_2	3=Oxo=Tetradecanoyl=ACP + NADPH <=> R=3=Hydroxyltetradecanoyl=ACP + NADP+
FAS2_7_1	Tetradecanoyl=ACP + Malonyl=[acyl=carrier protein] => 3=Oxo=Hexadecanoyl=ACP + Acyl=carrier protein + CO2
FAS2_7_2	3=Oxo=Hexadecanoyl=ACP + NADPH <=> R=3=Hydroxylhexadecanoyl=ACP + NADP+
FAS2_8_1	Hexadecanoyl=ACP + Malonyl=[acyl=carrier protein] => 3=Oxo=Octadecanoyl=ACP + Acyl=carrier protein + CO2
FAS2_8_2	3=Oxo=Octadecanoyl=ACP + NADPH <=> R=3=Hydroxyloctadecanoyl=ACP + NADP+
FAT1_1	ATP + Hexadecanoic_acid + CoA => AMP + Pyrophosphate + Hexadecanoyl=CoA
FAT1_2	ATP + Octadecanoic_acid + CoA => AMP + Pyrophosphate + Octadecanoyl=CoA
FAT1_3	ATP + Hexadecanoyl=9=ene_acid + CoA => AMP + Pyrophosphate + Hexadecanoyl=9=ene=CoA
FAT1_4	ATP + Octadecanoyl=9=ene_acid + CoA => AMP + Pyrophosphate + Octadecanoyl=9=ene=CoA
FAT1_5	ATP + Tetradecanoyl=9=ene_acid + CoA => AMP + Pyrophosphate + Tetradecanoyl=9=ene=CoA
FAT1_6	ATP + C24_acid + CoA => AMP + Pyrophosphate + C24=CoA
FAT1_7	ATP + C26_acid + CoA => AMP + Pyrophosphate + C26=CoA
FATP	ADP + Orthophosphate => ATP
FBA1	beta=D=Fructose 1,6=bisphosphate <=> Glycerone phosphate + D=Glyceraldehyde 3=phosphate
FBP1	beta=D=Fructose 1,6=bisphosphate => beta=D=Fructose 6=phosphate + Orthophosphate
FBP26	D=Fructose 2,6=bisphosphate => beta=D=Fructose 6=phosphate + Orthophosphate
FCY1	Cytosine => Uracil + NH3
FCY2_1	CYTSxt + H+EXT => Cytosine
FCY2_2	ADxt + H+EXT => Adenine
FCY2_3	GNxt + H+EXT <=> Guanine
FCY21_1	CYTSxt + H+EXT => Cytosine
FCY21_2	ADxt + H+EXT => Adenine
FCY21_3	GNxt + H+EXT <=> Guanine
FCY22_1	CYTSxt + H+EXT => Cytosine
FCY22_2	ADxt + H+EXT => Adenine
FCY22_3	GNxt + H+EXT <=> Guanine
FDH1	Formate + NAD+ => CO2 + NADH
FEN2	PNTOxt + H+EXT <=> (R)=Pantothenate
FIG4	1=Phosphatidyl=D=myo=inositol=3,5=bisphosphate => 1=Phosphatidyl=D=myo=inositol=3=phosphate + Pyrophosphate
FKS1	UDPglucose => 1,3=beta=D=Glucan + UDP
FKS3	UDPglucose => 1,3=beta=D=Glucan + UDP
FLX1	FAD + FMNM => FADM + FMN
FMN1_1	Riboflavin + ATP => FMN + ADP

FMN1_2	RiboflavinM + ATPM => FMNM + ADPM
FMT1	10=FormyltetrahydrofolateM + L=Methionyl=tRNAM => TetrahydrofolateM + N=Formylmethionyl=tRNAM
FNADH	NAD+ => NADH
FNADPH	NADP+ => NADPH
FOL1_1	2=Amino=4=hydroxy=6=(D=erythro=1,2,3=trihydroxypropyl)=7,8=dihydropteridine => 2=Amino=4=hydroxy=6=hydroxymethyl=7,8=dihydropteridine + Glycolaldehyde
FOL1_2	2=Amino=4=hydroxy=6=hydroxymethyl=7,8=dihydropteridine + ATP => AMP + 2=Amino=7,8=dihydro=4=hydroxy=6=(diphosphooxymethyl)pteridine
FOL1_3	4=Aminobenzoate + 2=Amino=7,8=dihydro=4=hydroxy=6=(diphosphooxymethyl)pteridine => Pyrophosphate + Dihydropteroate
FOL1_4	4=Aminobenzoate + 2=Amino=4=hydroxy=6=hydroxymethyl=7,8=dihydropteridine => Dihydropteroate
FOL2	GTP => Formate + 2=Amino=4=hydroxy=6=(erythro=1,2,3=trihydroxypropyl)=dihydropteridine triphosphate
FOL3	Tetrahydrofolate + ATP + L=Glutamate <=> ADP + Orthophosphate + Tetrahydrofolyl=[Glu](n)
FOX1_1	Trans=2=C18=CoA => 3=Hydroxy=C18=CoA
FOX1_2	Trans=2=C16=CoA => 3=Hydroxy=C16=CoA
FOX1_3	Trans=2=C14=CoA => 3=Hydroxy=C14=CoA
FOX1_4	trans=2=Dodecaenoyl=CoA => 3=hydroxy=Dodecanoyl=CoA
FOX1_5	trans=2=Decaenoyl=CoA => 3=hydroxy=Decanoyl=CoA
FOX1_6	trans=delta2=Octaenoyl=CoA => 3=hydroxy=Octanoyl=CoA
FOX1_7	trans=delta2=Hexaenoyl=CoA + H2O => 3=hydroxy=Hexanoyl=CoA
FOX1_8	trans=delta2=Butaenoyl=CoA + H2O => 3=hydroxy=Butanoyl=CoA
FOX2_1	3=Hydroxy=C18=CoA + NAD+ => 3=Keto=C18=CoA + NADPH
FOX2_2	3=Hydroxy=C16=CoA + NAD+ => 3=Keto=C16=CoA + NADPH
FOX2_3	3=Hydroxy=C14=CoA + NAD+ => 3=Keto=C14=CoA + NADPH
FOX2_4	3=hydroxy=Dodecanoyl=CoA + NAD+ => 3=keto=Dodecanoyl=CoA + NADH
FOX2_5	3=hydroxy=Decanoyl=CoA + NAD+ => 3=keto=Decanoyl=CoA + NADH
FOX2_6	3=hydroxy=Octanoyl=CoA + NAD+ => 3=keto=Octanoyl=CoA + NADH
FOX2_7	3=hydroxy=Hexanoyl=CoA + NAD+ => 3=keto=Hexanoyl=CoA + NADH
FOX2_8	3=hydroxy=Butanoyl=CoA + NAD+ => 3=keto=Butanoyl=CoA + NADH
FPS1	GLxt <=> Glycerol
FRS1	ATPM + L=Phenylalanine + tRNA(Phe) => AMP + Pyrophosphate + L=Phenylalanyl=tRNA(Phe)
FRS2	ATPM + L=Phenylalanine + tRNA(Phe) => AMP + Pyrophosphate + L=Phenylalanyl=tRNA(Phe)
FSP2_1	Maltose => 2.000000 alpha=D=Glucose
FSP2_2	D=Galalpha1=6D=Glucose => D=Galactose + alpha=D=Glucose
FUI1_1	URIxt + H+EXT => Uridine
FUI1_2	URIxt + H+EXT => Uridine
FUI1_3	URIxt + H+EXT => Uridine

FUM1_1	FumarateM <=> MalateM
FUM1_2	Fumarate <=> Malate
FUN63	IMP + NAD+ => NADH + Xanthosine 5'=phosphate
FUR1	Uracil + 5=Phospho=alpha=D=ribose 1=diphosphate => UMP + Pyrophosphate
FUR4	URAxt + H+EXT => Uracil
GAD1	L=Glutamate => 4=Aminobutanoate + CO2
GAL1	D=Galactose + ATP => D=Galactose 1=phosphate + ADP
GAL10	UDP=D=galactose <=> UDPglucose
GAL2_1	GLCxt => alpha=D=Glucose
GAL2_2	GLACxt => D=Galactose
GAL7_1	UTP + D=Galactose 1=phosphate <=> Pyrophosphate + UDP=D=galactose
GAL7_2	UDPglucose + D=Galactose 1=phosphate <=> D=Glucose 1=phosphate + UDP=D=galactose
GAP1_1	GLUxt <=> L=Glutamate
GAP1_10	ILExt + H+EXT <=> L=Isoleucine
GAP1_11	LEUxt + H+EXT <=> L=Leucine
GAP1_12	METxt + H+EXT <=> L=Methionine
GAP1_13	PHExt + H+EXT <=> L=Phenylalanine
GAP1_14	PROxt + H+EXT <=> L=Proline
GAP1_15	TRPxt + H+EXT <=> L=Tryptophan
GAP1_16	TYRxt + H+EXT <=> L=Tyrosine
GAP1_17	VALxt + H+EXT <=> L=Valine
GAP1_18	SERxt + H+EXT <=> L=Serine
GAP1_19	THRxt + H+EXT <=> L=Threonine
GAP1_2	ALAxt + H+EXT <=> L=Alanine
GAP1_20	LYSxt + H+EXT <=> L=Lysine
GAP1_21	ORNxt + H+EXT <=> L=Ornithine
GAP1_3	ARGxt + H+EXT <=> L=Arginine
GAP1_4	ASNxt + H+EXT <=> L=Asparagine
GAP1_5	ASPxt + H+EXT <=> L=Aspartate
GAP1_6	CYSxt + H+EXT <=> L=Cysteine
GAP1_7	GLYxt + H+EXT <=> Glycine
GAP1_8	GLNxt + H+EXT <=> L=Glutamine
GAP1_9	HISxt + H+EXT <=> L=Histidine
GCV1_1	GlycineM + TetrahydrofolateM + NAD+M => 5,10=MethylenetetrahydrofolateM + NADHM + CO2 + NH3

GCV1_2	Glycine + Tetrahydrofolate + NAD+ => 5,10=Methylenetetrahydrofolate + NADH + CO2 + NH3
GCV2	GlycineM + LipoamideM <=> S=AminomethyldihydrolipoylproteinM + CO2M
GDE1	Glycerophosphatidylcholine => Choline + sn=Glycerol 3=phosphate
GDH1	2=Oxoglutarate + NH3 + NADPH => L=Glutamate + NADP+
GDH2	L=Glutamate + NAD+ => 2=Oxoglutarate + NH3 + NADH
GDH3	2=Oxoglutarate + NH3 + NADPH => L=Glutamate + NADP+
GFA1	beta=D=Fructose 6=phosphate + L=Glutamine => L=Glutamate + D=Glucosamine 6=phosphate
GIT1_1	GROPCxt <=> Glycerophosphatidylcholine
GIT1_2	GROPIxt <=> Glycerophosphatidyl=D=myo=inositol
GLC3	Glycogen + Orthophosphate => D=Glucose 1=phosphate
GLK1_1	alpha=D=Glucose + ATP => alpha=D=Glucose 6=phosphate + ADP
GLK1_2	alpha=D=Mannose + ATP => D=Mannose 6=phosphate + ADP
GLK1_3	beta=D=Glucose + ATP => beta=D=Glucose 6=phosphate + ADP
GLN1	L=Glutamate + NH3 + ATP => L=Glutamine + ADP + Orthophosphate
GLN4	L=Glutamine + ATP => L=Glutaminyl=tRNA(Gln) + AMP + Pyrophosphate
GLO1	Glutathione + Methylglyoxal <=> (R)=S=Lactoylglutathione
GLO2	(R)=S=Lactoylglutathione => Glutathione + (R)=Lactate
GLO4	(R)=S=LactoylglutathioneM => GlutathioneM + (R)=LactateM
GLR1	NADPH + Oxidized glutathione => NADP+ + Glutathione
GLT1	2=Oxoglutarate + L=Glutamine + NADH => NAD+ + 2.000000 L=Glutamate
GLY1	Glycine + Acetaldehyde => L=Threonine
GNA1	Acetyl=CoA + D=Glucosamine 6=phosphate <=> CoA + N=Acetyl=D=glucosamine 6=phosphate
GND1	6=Phospho=D=gluconate + NADP+ => NADPH + CO2 + D=Ribulose 5=phosphate
GND2	6=Phospho=D=gluconate + NADP+ => NADPH + CO2 + D=Ribulose 5=phosphate
GNP1_1	ASNxt + H+EXT <=> L=Asparagine
GNP1_2	CYSxt + H+EXT <=> L=Cysteine
GNP1_3	GLNxt + H+EXT <=> L=Glutamine
GNP1_4	LEUxt + H+EXT <=> L=Leucine
GNP1_5	METxt + H+EXT <=> L=Methionine
GNP1_6	SERxt + H+EXT <=> L=Serine
GNP1_7	THRxt + H+EXT <=> L=Threonine
GPD1	Glycerone phosphate + NADH => sn=Glycerol 3=phosphate + NAD+
GPD2	Glycerone phosphate + NADH => sn=Glycerol 3=phosphate + NAD+
GPH1	Glycogen + Orthophosphate => D=Glucose 1=phosphate

GPM1_1	3=Phospho=D=glyceroyl phosphate <=> 2,3=Bisphospho=D=glycerate
GPM1_2	3=Phospho=D=glycerate <=> 2=Phospho=D=glycerate
GPM2	3=Phospho=D=glycerate <=> 2=Phospho=D=glycerate
GPM3	3=Phospho=D=glycerate <=> 2=Phospho=D=glycerate
GPT2_1	sn=Glycerol 3=phosphate + AcylCoAs => Acyl=sn=glycerol 3=phosphate + CoA
GPT2_2	Glycerone phosphate + AcylCoAs => Acyldihydroxyacetone phosphate + CoA
GPX1	2.000000 Glutathione + H2O2 <=> Oxidized glutathione
GPX2	2.000000 Glutathione + H2O2 <=> Oxidized glutathione
GRS1	ATP + Glycine + tRNA(Gly) => AMP + Pyrophosphate + L=Glyciyl=tRNA(Gly)
GRS2	ATP + Glycine + tRNA(Gly) => AMP + Pyrophosphate + L=Glyciyl=tRNA(Gly)
GSC2	UDPglucose => 1,3=beta=D=Glucan + UDP
GSH1	L=Cysteine + L=Glutamate + ATP => gamma=L=Glutamyl=L=cysteine + Orthophosphate + ADP
GSH2	Glycine + gamma=L=Glutamyl=L=cysteine + ATP => Glutathione + Orthophosphate + ADP
GSY1	UDPglucose => UDP + Glycogen
GSY2	UDPglucose => UDP + Glycogen
GUA1	Xanthosine 5'=phosphate + ATP + L=Glutamine => L=Glutamate + AMP + Pyrophosphate + GMP
GUK1_1	GMP + ATP <=> GDP + ADP
GUK1_2	dGMP + ATP <=> dGDP + ADP
GUK1_3	GMP + dATP <=> GDP + dADP
GUT1	Glycerol + ATP => sn=Glycerol 3=phosphate + ADP
GUT2	sn=Glycerol 3=phosphate + FADM => Glycerone phosphate + FADH2M
HEM1	Succinyl=CoAM + GlycineM => 5=AminolevulinateM + CoAM + CO2M
HEM12	Uroporphyrinogen III => 4.000000 CO2 + Coproporphyrinogen
HEM13	Oxygen + Coproporphyrinogen => 2.000000 CO2 + Protoporphyrinogen IX
HEM14	Oxygen + Protoporphyrinogen IXM => ProtoporphyrinM
HEM15	ProtoporphyrinM => HemeM
HEM2	2.000000 5=Aminolevulinate => Porphobilinogen
HEM3	4.000000 Porphobilinogen => Hydroxymethylbilane + 4.000000 NH3
HEM4	Hydroxymethylbilane => Uroporphyrinogen III
HFA1	Acetyl=CoAM + ATPM + CO2 <=> Malonyl=CoAM + ADPM + OrthophosphateM
HIP1	HISxt + H+EXT <=> L=Histidine
HIS1	5=Phospho=alpha=D=ribose 1=diphosphate + ATP => Pyrophosphate + N1=(5=Phospho=D=ribosyl)=ATP
HIS2	L=Histidinol phosphate => Orthophosphate + L=Histidinol
HIS3	D=erythro=1=(Imidazol=4=yl)glycerol 3=phosphate => 3=(Imidazol=4=yl)=2=oxopropyl phosphate

HIS4_1	N1=(5=Phospho=D=ribosyl)=ATP => Pyrophosphate + N1=(5=Phospho=D=ribosyl)=AMP
HIS4_2	N1=(5=Phospho=D=ribosyl)=AMP => 5=(5=Phospho=D=ribosylaminoformimino)=1=(5=phosphoribosyl)=imidazole=4=carboxamide
HIS4_3	L=Histidinol + 2.000000 NAD+ => L=Histidine + 2.000000 NADH
HIS5	3=(Imidazol=4=yl)=2=oxopropyl phosphate + L=Glutamate => 2=Oxoglutarate + L=Histidinol phosphate
HIS6	5=(5=Phospho=D=ribosylaminoformimino)=1=(5=phosphoribosyl)=imidazole=4=carboxamide => "N=(5'=Phospho=D=1'=ribulosylformimino)=5=amino=1=(5''''=phospho=D=ribosyl)=4=imidazolecarboxamide"
HIS7	"N=(5'=Phospho=D=1'=ribulosylformimino)=5=amino=1=(5''''=phospho=D=ribosyl)=4=imidazolecarboxamide" + L=Glutamine => L=Glutamate + 1=(5'=Phosphoribosyl)=5=amino=4=imidazolecarboxamide + D=erythro=1=(Imidazo
HMG1	(R)=Mevalonate + CoA + 2.000000 NADP+ <=> (S)=3=Hydroxy=3=methylglutaryl=CoA + 2.000000 NADPH
HMG2	(R)=Mevalonate + CoA + 2.000000 NADP+ <=> (S)=3=Hydroxy=3=methylglutaryl=CoA + 2.000000 NADPH
HMGS	(S)=3=Hydroxy=3=methylglutaryl=CoA + CoA <=> Acetyl=CoA + Acetoacetyl=CoA
HMT1	S=Adenosyl=L=methionine + L=Histidine => S=Adenosyl=L=homocysteine + N(pai)=Methyl=L=histidine
HNM1	CHOxt + H+EXT => Choline
HOM2	4=Phospho=L=aspartate + NADPH => NADP+ + Orthophosphate + L=Aspartate 4=semialdehyde
HOM3	L=Aspartate + ATP => ADP + 4=Phospho=L=aspartate
HOM6_1	L=Aspartate 4=semialdehyde + NADH => NAD+ + L=Homoserine
HOM6_2	L=Aspartate 4=semialdehyde + NADPH => NADP+ + L=Homoserine
HOR2	sn=Glycerol 3=phosphate => Glycerol + Orthophosphate
HPT1_1	HYXN + 5=Phospho=alpha=D=ribose 1=diphosphate => Pyrophosphate + IMP
HPT1_2	Guanine + 5=Phospho=alpha=D=ribose 1=diphosphate => Pyrophosphate + GMP
HTD2_1	R=3=Hydroxylbutanoyl=ACPM => trans=But=2=enoyl=ACPM
HTD2_2	R=3=Hydroxylhexanoyl=ACPM => trans=Hex=2=enoyl=ACPM
HTD2_3	R=3=Hydroxyloctanoyl=ACPM => trans=Oct=2=enoyl=ACPM
HTD2_4	R=3=Hydroxyldecanoyl=ACPM => trans=Dec=2=enoyl=ACPM
HTD2_5	R=3=Hydroxyldodecanoyl=ACPM => trans=Dodec=2=enoyl=ACPM
HTD2_6	R=3=Hydroxyltetradecanoyl=ACPM => trans=Tetradec=2=enoyl=ACPM
HTD2_7	R=3=Hydroxylhexadecanoyl=ACPM => trans=Hexadec=2=enoyl=ACPM
HTD2_8	R=3=Hydroxyloctadecanoyl=ACPM => trans=Octadec=2=enoyl=ACPM
HTS1	ATP + L=Histidine + tRNA(His) => AMP + Pyrophosphate + L=Histidyl=tRNA(His)
HXK1_1	beta=D=Glucose + ATP => alpha=D=Glucose 6=phosphate + ADP
HXK1_2	alpha=D=Glucose + ATP => alpha=D=Glucose 6=phosphate + ADP
HXK1_3	alpha=D=Mannose + ATP => D=Mannose 6=phosphate + ADP
HXK1_4	ATP + D=Fructose => ADP + beta=D=Fructose 6=phosphate
HXK2_1	beta=D=Glucose + ATP => alpha=D=Glucose 6=phosphate + ADP
HXK2_2	alpha=D=Glucose + ATP => alpha=D=Glucose 6=phosphate + ADP
HXK2_3	alpha=D=Mannose + ATP => D=Mannose 6=phosphate + ADP

HXK2_4	ATP + D=Fructose => ADP + beta=D=Fructose 6=phosphate
HXT1_1	GLCxt => alpha=D=Glucose
HXT1_2	FRUxt => D=Fructose
HXT1_3	MANxt => alpha=D=Mannose
HXT10_1	GLCxt => alpha=D=Glucose
HXT10_2	GLACxt => D=Galactose
HXT10_3	FRUxt => D=Fructose
HXT10_4	MANxt => alpha=D=Mannose
HXT11_1	GLCxt => alpha=D=Glucose
HXT11_2	GLCxt => alpha=D=Glucose
HXT11_3	GLACxt => D=Galactose
HXT11_4	FRUxt => D=Fructose
HXT11_5	MANxt => alpha=D=Mannose
HXT13_1	GLCxt => alpha=D=Glucose
HXT13_2	FRUxt => D=Fructose
HXT13_3	MANxt => alpha=D=Mannose
HXT14	GLACxt => D=Galactose
HXT15_1	GLCxt => alpha=D=Glucose
HXT15_2	FRUxt => D=Fructose
HXT15_3	MANxt => alpha=D=Mannose
HXT16_1	GLCxt => alpha=D=Glucose
HXT16_2	FRUxt => D=Fructose
HXT16_3	MANxt => alpha=D=Mannose
HXT17_1	GLCxt => alpha=D=Glucose
HXT17_2	FRUxt => D=Fructose
HXT17_3	MANxt => alpha=D=Mannose
HXT2_1	GLCxt => alpha=D=Glucose
HXT2_2	FRUxt => D=Fructose
HXT2_3	MANxt => alpha=D=Mannose
HXT3_1	GLCxt => alpha=D=Glucose
HXT3_2	FRUxt => D=Fructose
HXT3_3	MANxt => alpha=D=Mannose
HXT4_1	GLCxt => alpha=D=Glucose
HXT4_2	GLCxt => alpha=D=Glucose

HXT4_3	FRUxt => D=Fructose
HXT4_4	MANxt => alpha=D=Mannose
HXT5_1	GLCxt => alpha=D=Glucose
HXT5_2	FRUxt => D=Fructose
HXT5_3	MANxt => alpha=D=Mannose
HXT6_1	GLCxt => alpha=D=Glucose
HXT6_2	FRUxt => D=Fructose
HXT6_3	MANxt => alpha=D=Mannose
HXT7_1	GLCxt => alpha=D=Glucose
HXT7_2	FRUxt => D=Fructose
HXT7_3	MANxt => alpha=D=Mannose
HXT8_1	GLCxt => alpha=D=Glucose
HXT8_2	FRUxt => D=Fructose
HXT8_3	MANxt => alpha=D=Mannose
HXT9_1	GLCxt => alpha=D=Glucose
HXT9_2	GLACxt => D=Galactose
HXT9_3	FRUxt => D=Fructose
HXT9_4	MANxt => alpha=D=Mannose
HYR1	2.000000 Glutathione + H2O2 <=> Oxidized glutathione
ICL1	Isocitrate => Glyoxylate + Succinate
ICL2	Isocitrate => Glyoxylate + Succinate
IDH1	IsocitrateM + NAD+M => CO2M + NADHM + 2=OxoglutarateM
IDI1	Isopentenyl diphosphate <=> Dimethylallyl diphosphate
IDP1_1	IsocitrateM + NADP+M => NADPHM + OxalosuccinateM
IDP1_2	OxalosuccinateM => CO2M + 2=OxoglutarateM
IDP2_1	Isocitrate + NADP+ => NADPH + Oxalosuccinate
IDP2_2	Oxalosuccinate => CO2 + 2=Oxoglutarate
IDP3_1	Isocitrate + NADP+ => NADPH + Oxalosuccinate
IDP3_2	Oxalosuccinate => CO2 + 2=Oxoglutarate
IFA38_1	3=Keto=C14=CoA + NADPH => 3=Hydroxy=C14=CoA + NADP+
IFA38_2	3=Keto=C16=CoA + NADPH => 3=Hydroxy=C16=CoA + NADP+
IFA38_3	3=Keto=C18=CoA + NADPH => 3=Hydroxy=C18=CoA + NADP+
IFA38_4	3=Keto=C20=CoA + NADPH => 3=Hydroxy=C20=CoA + NADP+
IFA38_5	

IFA38_6	3=Keto=C24=CoA + NADPH => 3=Hydroxy=C24=CoA + NADP+
IFA38_7	3=Keto=C26=CoA + NADPH => 3=Hydroxy=C26=CoA + NADP+
ILS1	ATP + L=Isoleucine + tRNA(Ile) => AMP + Pyrophosphate + L=Isoleucyl=tRNA(Ile)
ILV1	L=ThreonineM => NH3M + 2=OxobutanoateM
ILV2_1	2=OxobutanoateM + PyruvateM => 2=Aceto=2=hydroxy butyrateM + CO2M
ILV2_2	2.000000 PyruvateM => CO2M + 2=AcetolactateM
ILV3_1	(R)=3=Hydroxy=3=methyl=2=oxobutanoateM => (R)=2=OxoisovalerateM
ILV3_2	(R)=2,3=dihydroxy=3=methylbutanoateM => 3=Methyl=2=oxobutanoateM
ILV5_1	2=AcetolactateM + NADPHM => NADP+M + (R)=3=Hydroxy=3=methyl=2=oxobutanoateM
ILV5_2	2=Aceto=2=hydroxy butyrateM + NADPHM => NADP+M + (R)=2,3=dihydroxy=3=methylbutanoateM
ILV5_3	2=DehydropantoateM + NADPHM => NADP+M + (R)=PantoateM
IMD3	IMP + NAD+ => NADH + Xanthosine 5'=phosphate
IMD4	IMP + NAD+ => NADH + Xanthosine 5'=phosphate
INM1	1L=myo=Inositol 1=phosphate => myo=Inositol + Orthophosphate
INO1	alpha=D=Glucose 6=phosphate => 1L=myo=Inositol 1=phosphate
INP51	1=Phosphatidyl=D=myo=inositol=4,5=bisphosphate => 1=Phosphatidyl=D=myo=inositol=4=phosphate + Pyrophosphate
INP52_1	1=Phosphatidyl=D=myo=inositol=3=phosphate => 1=Phosphatidyl=D=myo=inositol + Pyrophosphate
INP52_2	1=Phosphatidyl=D=myo=inositol=4=phosphate => 1=Phosphatidyl=D=myo=inositol + Pyrophosphate
INP52_3	1=Phosphatidyl=D=myo=inositol=3,5=bisphosphate => 1=Phosphatidyl=D=myo=inositol=3=phosphate + Pyrophosphate
INP52_4	1=Phosphatidyl=D=myo=inositol=4,5=bisphosphate => 1=Phosphatidyl=D=myo=inositol=4=phosphate + Pyrophosphate
INP53_1	1=Phosphatidyl=D=myo=inositol=3=phosphate => 1=Phosphatidyl=D=myo=inositol + Pyrophosphate
INP53_2	1=Phosphatidyl=D=myo=inositol=4=phosphate => 1=Phosphatidyl=D=myo=inositol + Pyrophosphate
INP53_3	1=Phosphatidyl=D=myo=inositol=3,5=bisphosphate => 1=Phosphatidyl=D=myo=inositol=3=phosphate + Pyrophosphate
INP53_4	1=Phosphatidyl=D=myo=inositol=4,5=bisphosphate => 1=Phosphatidyl=D=myo=inositol=4=phosphate + Pyrophosphate
INP54	1=Phosphatidyl=D=myo=inositol=4,5=bisphosphate => 1=Phosphatidyl=D=myo=inositol=4=phosphate + Pyrophosphate
IPK1	myo=Inositol + 6 ATP => IP6
IPP1	Pyrophosphate => 2.000000 Orthophosphate
IPT1_1	Mannose=inositol=P=D=ceramide + 1=Phosphatidyl=D=myo=inositol => Inositol=mannose=P=inositol=P=D=ceramide
IPT1_2	Mannose=inositol=P=P=ceramide + 1=Phosphatidyl=D=myo=inositol => Inositol=mannose=P=inositol=P=P=ceramide
ISC1_1	2.000000 Phosphatidylcholine => Choline phosphate + Diacylglycerol
ISC1_2_1	Inositol=phosphoryl=D=ceramide => D=Ceramide + 1=Phosphatidyl=D=myo=inositol
ISC1_2_2	Inositol=phosphoryl=P=ceramide => P=Ceramide + 1=Phosphatidyl=P=myo=inositol
ISM1	ATPM + L=Isoleucine + tRNA(Ile)M => AMPM + PyrophosphateM + L=Isoleucyl=tRNA(Ile)M
ITR1	MIxt + H+EXT => myo=Inositol

ITR2	MIxt + H+EXT => myo=Inositol
JEN1_1	LACxt + H+EXT <=> (R)=Lactate
JEN1_2	PYRxt + H+EXT <=> Pyruvate
KGD1	2=OxoglutarateM + NAD+M + CoAM => CO2M + NADHM + Succinyl=CoAM
KRE2	beta=D=Mannosyldiacetylchitobiosyldiphosphodolichol + 2.000000 GDPmannose => 2.000000 GDP + "(""alpha""=D=mannosyl)(,2)=""beta""=D=mannosyl=diacetylchitobiosyldiphosphod olichol"
KRS1	ATP + L=Lysine + tRNA(Lys) => AMP + Pyrophosphate + L=Lysyl=tRNA(Lys)
KTR1	beta=D=Mannosyldiacetylchitobiosyldiphosphodolichol + 2.000000 GDPmannose => 2.000000 GDP + "(""alpha""=D=mannosyl)(,2)=""beta""=D=mannosyl=diacetylchitobiosyldiphosphod olichol"
KTR2	beta=D=Mannosyldiacetylchitobiosyldiphosphodolichol + 2.000000 GDPmannose => 2.000000 GDP + "(""alpha""=D=mannosyl)(,2)=""beta""=D=mannosyl=diacetylchitobiosyldiphosphod olichol"
KTR3	beta=D=Mannosyldiacetylchitobiosyldiphosphodolichol + 2.000000 GDPmannose => 2.000000 GDP + "(""alpha""=D=mannosyl)(,2)=""beta""=D=mannosyl=diacetylchitobiosyldiphosphod olichol"
KTR4	beta=D=Mannosyldiacetylchitobiosyldiphosphodolichol + 2.000000 GDPmannose => 2.000000 GDP + "(""alpha""=D=mannosyl)(,2)=""beta""=D=mannosyl=diacetylchitobiosyldiphosphod olichol"
KTR6	beta=D=Mannosyldiacetylchitobiosyldiphosphodolichol + 2.000000 GDPmannose => 2.000000 GDP + "(""alpha""=D=mannosyl)(,2)=""beta""=D=mannosyl=diacetylchitobiosyldiphosphod olichol"
LAC1_1	Sphinganine + LongAcylCoA => D=Ceramide + CoA
LAC1_2	Phytosphingosine + LongAcylCoA => P=Ceramide + CoA
LAG1_1	Sphinganine + LongAcylCoA => D=Ceramide + CoA
LAG1_2	Phytosphingosine + LongAcylCoA => P=Ceramide + CoA
LAT1	S=acetyldihydrolipoamideM + CoAM => Acetyl=CoAM + DihydrolipoamideM
LCB1	Hexadecanoyl=CoA + L=Serine => CoA + 3=Dehydrosphinganine + CO2
LCB2	Hexadecanoyl=CoA + L=Serine => CoA + 3=Dehydrosphinganine + CO2
LCB3_1	Sphinganine 1=phosphate => Sphinganine + Orthophosphate
LCB3_2	Phytosphingosine 1=phosphate => Phytosphingosine + Orthophosphate
LCB4_1	Sphinganine + ATP => Sphinganine 1=phosphate + ADP
LCB4_2	Phytosphingosine + ATP => Phytosphingosine 1=phosphate + ADP
LCB5_1	Sphinganine + ATP => Sphinganine 1=phosphate + ADP
LCB5_2	Phytosphingosine + ATP => Phytosphingosine 1=phosphate + ADP
LEU1_1	3=Isopropylmalate <=> 2=Isopropylmalate
LEU1_2	2=Isopropylmaleate <=> 2=Isopropylmalate
LEU2	2=Isopropylmalate + NAD+ => NADH + 3=Carboxy=4=methyl=2=oxopentanoate + CO2
LEU4	Acetyl=CoAM + (R)=2=OxoisovalerateM => CoAM + 2=IsopropylmalateM
LongAcyl	C26=CoA => LongAcylCoA
LPD1	DihydrolipoamideM + NAD+M => LipoamideM + NADHM
LPP1	Diacylglycerol pyrophosphate => Phosphatidate + Orthophosphate
LRO1	Phosphatidylcholine + Diacylglycerol => Glycerophosphatidylcholine + CoA
LSB6	ATP + 1=Phosphatidyl=D=myo=inositol => ADP + 1=Phosphatidyl=D=myo=inositol=4=phosphate
LSC1	ATPM + ItaconateM + CoAM <=> ADPM + OrthophosphateM + Itaconyl=CoAM

LSC2	ATPM + SuccinateM + CoAM <=> ADPM + OrthophosphateM + Succinyl=CoAM
LYP1	LYSxt + H+EXT <=> L=Lysine
LYS1	N6=(L=1,3=Dicarboxypropyl)=L=lysine + NAD+ <=> L=Lysine + 2=Oxoglutarate + NADH
LYS12	HomoisocitrateM + NAD+M <=> OxaloglutarateM + CO2M + NADHM
LYS2_1	L=2=Aminoadipate + NADPH + ATP => L=2=Aminoadipate 6=semialdehyde + NADP+ + AMP + Pyrophosphate
LYS2_2	L=2=Aminoadipate + NADH + ATP => L=2=Aminoadipate 6=semialdehyde + NAD+ + AMP + Pyrophosphate
LYS20_1	Acetyl=CoA + 2=Oxoglutarate => 2=Hydroxybutane=1,2,4=tricarboxylate + CoA
LYS20_2	Acetyl=CoAM + 2=OxoglutarateM => 2=Hydroxybutane=1,2,4=tricarboxylateM + CoAM
LYS21	Acetyl=CoA + 2=Oxoglutarate => 2=Hydroxybutane=1,2,4=tricarboxylate + CoA
LYS4	HomoisocitrateM <=> But=1=ene=1,2,4=tricarboxylateM
LYS9	L=Glutamate + L=2=Aminoadipate 6=semialdehyde + NADPH <=> N6=(L=1,3=Dicarboxypropyl)=L=lysine + NADP+
MAE1	MalateM + NADP+M => CO2M + NADPHM + PyruvateM
MAL11	MLTxt + H+EXT => Maltose
MAL12	Maltose => 2.000000 alpha=D=Glucose
MAL31	MALxt + H+EXT <=> Malate
MAL32	Maltose => 2.000000 alpha=D=Glucose
MCT1	Malonyl=CoAM + Acyl=carrier proteinM <=> Malonyl=[acyl=carrier protein]M + CoAM
MDH1	MalateM + NAD+M <=> NADHM + OxaloacetateM
MDH2	Malate + NAD+ <=> NADH + Oxaloacetate
MDH3	Malate + NAD+ <=> NADH + Oxaloacetate
MEP1	NH3xt <=> NH3
MEP2	NH3xt <=> NH3
MEP3	NH3xt <=> NH3
MES1	ATP + L=Methionine + tRNA(Met) => AMP + Pyrophosphate + L=Methionyl=tRNA(Met)
MET1	S=Adenosyl=L=methionine + Uroporphyrinogen III => S=Adenosyl=L=homocysteine + Sirohydrochlorin
MET10	Sulfite + 3.000000 NADPH <=> Hydrogen sulfide + 3.000000 NADP+
MET12	5,10=MethylenetetrahydrofolateM + NADPHM => NADP+M + 5=MethyltetrahydrofolateM
MET13	5,10=MethylenetetrahydrofolateM + NADPHM => NADP+M + 5=MethyltetrahydrofolateM
MET14	Adenylylsulfate + ATP => ADP + 3'=Phosphoadenylylsulfate
MET16	3'=Phosphoadenylylsulfate + Reduced thioredoxin => Oxidized thioredoxin + Sulfite + Adenosine 3',5'=bisphosphate
MET17_1	O=Acetyl=L=homoserine + Methanethiol => L=Methionine + Acetate
MET17_2	O=Acetyl=L=homoserine + Hydrogen sulfide => Acetate + Homocysteine
MET17_3	O=Acetyl=L=homoserine + Hydrogen sulfide => Acetate + Homocysteine
MET2	Acetyl=CoA + L=Homoserine <=> CoA + O=Acetyl=L=homoserine

MET22	Adenosine 3',5'=bisphosphate => AMP + Orthophosphate
MET3	Sulfate + ATP => Pyrophosphate + Adenylylsulfate
MET6	Homocysteine + 5=Methyltetrahydropteroyltri=L=glutamate => Tetrahydropteroyltri=L=glutamate + L=Methionine
MET7	Tetrahydrofolate + ATP + L=Glutamate <=> ADP + Orthophosphate + Tetrahydrofolyl=[Glu](n)
MHT1	S=Adenosyl=L=methionine + Homocysteine => S=Adenosyl=L=homocysteine + L=Methionine
MIR1	Orthophosphate <=> H+M + OrthophosphateM
MIS1_1	5,10=MethylenetetrahydrofolateM + NADP+M <=> 5,10=MethenyltetrahydrofolateM + NADPHM
MIS1_2	TetrahydrofolateM + FormateM + ATPM => ADPM + OrthophosphateM + 10=FormyltetrahydrofolateM
MIS1_3	5,10=MethenyltetrahydrofolateM <=> 10=FormyltetrahydrofolateM
MLS1	Acetyl=CoA + Glyoxylate => CoA + Malate
MMP1	MMETxt + H+EXT => S=Methylmethionine
MPH2	MLTxt + H+EXT => Maltose
MPH3	MLTxt + H+EXT => Maltose
MSD1	ATPM + L=AspartateM + tRNA(Asp)M => AMPM + PyrophosphateM + L=Aspartyl=tRNA(Asp)M
MSE1	GlutamateM + ATPM => L=Glutamyl=tRNA(Glu)M + AMPM + PyrophosphateM
MSF1	ATPM + L=Phenylalanine + tRNA(Phe)M => AMPM + PyrophosphateM + L=Phenylalanyl=tRNA(Phe)M
MSK1	ATPM + L=LysineM + tRNA(Lys)M => AMPM + PyrophosphateM + L=lysyl=tRNA(Lys)M
MSM1	ATPM + L=Methionine + tRNA(Met)M => AMPM + Pyrophosphate + L=Methionyl=tRNA(Met)M
MSR1	ATP + L=Arginine + tRNA(Arg) => AMP + Pyrophosphate + L=Arginyl=tRNA(Arg)
MSS4	1=Phosphatidyl=D=myo=inositol=4=phosphate + ATP => 1=Phosphatidyl=D=myo=inositol=4,5=bisphosphate + ADP
MST1	ATPM + L=ThreonineM + tRNA(Thr)M => AMPM + Pyrophosphate + L=Threonyl=tRNA(Thr)M
MSW1	ATPM + L=TryptophanM + tRNAM => AMPM + PyrophosphateM + L=Tryptophanyl=tRNA(Trp)M
MSY1	ATPM + L=Tyrosine + tRNA(Tyr)M => AMPM + PyrophosphateM + L=Tyrosyl=tRNA(Tyr)M
MTD1	5,10=Methylenetetrahydrofolate + NAD+ => 5,10=Methenyltetrahydrofolate + NADH
MUP1	METxt + H+EXT <=> L=Methionine
MUP3	METxt + H+EXT <=> L=Methionine
MUQ1	Ethanolamine phosphate + CTP => CDPethanolamine + Pyrophosphate
MVD1	ATP + (R)=5=Diphosphomevalonate => ADP + Orthophosphate + Isopentenyl diphosphate + CO2
NAM2	ATPM + L=Leucine + tRNA(Leu)M => AMPM + Pyrophosphate + L=Leucyl=tRNA(Leu)M
NAT1	Acetyl=CoA + Peptide => CoA + Nalpha=Acetylpeptide
NAT2	Acetyl=CoA + Peptide => CoA + Nalpha=Acetylpeptide
NCP1	NADPH + 2.000000 Ferricytochrome cM => NADP+ + 2.000000 Ferrocytochrome cM
NDH1	NADH + Ubiquinone=9M => UbiquinolM + NAD+
NDH2	NADH + Ubiquinone=9M => UbiquinolM + NAD+

NDI1	NADHM + Ubiquinone=9M => UbiquinolM + NAD+M
NHA1	NAxt <=> Sodium + H+EXT
NIT2_1	3=Indoleacetonitrile => Indoleacetate + NH3
NIT2_2	alpha=Aminopropiononitrile => L=Alanine + NH3
NIT2_3	gamma=Amino=gamma=cyanobutanoate => L=Glutamate + NH3
NMT1	Tetradecanoyl=CoA + Glycylpeptide => CoA + N=Tetradecanoylglycylpeptide
NPT1_1	Nicotinate + 5=Phospho=alpha=D=ribose 1=diphosphate => Nicotinate D=ribonucleotide + Pyrophosphate
NPT1_2	NicotinateM + 5=Phospho=alpha=D=ribose 1=diphosphateM => Nicotinate D=ribonucleotideM + PyrophosphateM
NTE1	Phosphatidylcholine => Glycerophosphatidylcholine + Acyl_acids
NTH1	alpha,alpha=Trehalose => 2.000000 alpha=D=Glucose
NTH2	alpha,alpha=Trehalose => 2.000000 alpha=D=Glucose
OAC1	Oxaloacetate <=> OxaloacetateM + H+M
OAR1_1	Acetoacetyl=[acyl=carrier protein]M + NADPHM <=> R=3=Hydroxylbutanoyl=ACPM + NADP+M
OAR1_2	3=Oxo=Hexanoyl=ACPM + NADPHM <=> R=3=Hydroxylhexanoyl=ACPM + NADP+M
OAR1_3	3=Oxo=Octanoyl=ACPM + NADPHM <=> R=3=Hydroxyloctanoyl=ACPM + NADP+M
OAR1_4	3=Oxo=Decanoyl=ACPM + NADPHM <=> R=3=Hydroxyldecanoyl=ACPM + NADP+M
OAR1_5	3=Oxo=Dodecanoyl=ACPM + NADPHM <=> R=3=Hydroxyldodecanoyl=ACPM + NADP+M
OAR1_6	3=Oxo=Tetradecanoyl=ACPM + NADPHM <=> R=3=Hydroxyltetradecanoyl=ACPM + NADP+M
OAR1_7	3=Oxo=Hexadecanoyl=ACPM + NADPHM <=> R=3=Hydroxylhexadecanoyl=ACPM + NADP+M
OAR1_8	3=Oxo=Octadecanoyl=ACPM + NADPHM <=> R=3=Hydroxyloctadecanoyl=ACPM + NADP+M
ODC1	2=OxoglutarateM + Oxaloglutarate <=> 2=Oxoglutarate + OxaloglutarateM
ODC2	2=OxoglutarateM + Oxaloglutarate <=> 2=Oxoglutarate + OxaloglutarateM
OLE1_c14	Tetradecanoyl=CoA + Oxygen => Tetradecanoyl=9=ene=CoA
OLE1_c16	Hexadecanoyl=ACP + Oxygen => Hexadecanoyl=9=ene=CoA
OLE1_c18	Octadecanoyl=ACP + Oxygen => Octadecanoyl=9=ene=CoA
OPI3_1	S=Adenosyl=L=methionine + Phosphatidyl=N=methylethanolamine => S=Adenosyl=L=homocysteine + Phosphatidyl=N=dimethylethanolamine
OPI3_2	Phosphatidyl=N=dimethylethanolamine + S=Adenosyl=L=methionine => Phosphatidylcholine + S=Adenosyl=L=homocysteine
ORT1	L=Ornithine + H+M <=> L=OrnithineM
OSM1	FADH2M + FumarateM => SuccinateM + FADM
PAD1	3=Nonaprenyl=4=hydroxybenzoate => CO2 + 2=Nonaprenylphenol
PAN5	2=Dehydropantoate + NADPH => NADP+ + (R)=Pantoate
PCK1	Oxaloacetate + ATP => Phosphoenolpyruvate + CO2 + ADP
PCM1_1	N=Acetyl=D=glucosamine 1=phosphate <=> N=Acetyl=D=glucosamine 6=phosphate
PCM1_2	D=Glucosamine 6=phosphate <=> D=Glucosamine 1=phosphate

PCT1	Choline phosphate + CTP => CDPcholine + Pyrophosphate
PDA1	PyruvateM + LipoamideM => S=acetyldihydrolipoamideM + CO2M
PDC1	Pyruvate => CO2 + Acetaldehyde
PDC5	Pyruvate => CO2 + Acetaldehyde
PDC6	Pyruvate => CO2 + Acetaldehyde
PDE1	3',5'=Cyclic AMP => AMP
PDE2_1	3',5'=Cyclic AMP => AMP
PDE2_2	3',5'=Cyclic dAMP => dAMP
PDE2_3	3',5'=Cyclic IMP => IMP
PDE2_4	3',5'=Cyclic GMP => GMP
PDE2_5	3',5'=Cyclic CMP => CMP
PDX3_1	Pyridoxamine phosphate + Oxygen => Pyridoxal phosphate + H2O2 + NH3
PDX3_2	Pyridoxine phosphate + Oxygen <=> Pyridoxal phosphate + H2O2
PDX3_3	Pyridoxine + Oxygen <=> Pyridoxal + H2O2
PDX3_4	Pyridoxal + Oxygen + NH3 <=> Pyridoxamine + H2O2
PDX3_5	Pyridoxamine phosphate + Oxygen => Pyridoxal phosphate + H2O2 + NH3
PET9	ADP + ATPM + Orthophosphate => H+M + ADPM + ATP + OrthophosphateM
PFK1_1	beta=D=Fructose 6=phosphate + ATP => beta=D=Fructose 1,6=bisphosphate + ADP
PFK1_2	ATP + D=Tagatose 6=phosphate => ADP + D=Tagatose 1,6=bisphosphate
PFK1_3	ATP + Sedoheptulose 7=phosphate => ADP + Sedoheptulose 1,7=bisphosphate
PFK2	beta=D=Fructose 6=phosphate + ATP => beta=D=Fructose 1,6=bisphosphate + ADP
PFK26	ATP + beta=D=Fructose 6=phosphate => ADP + D=Fructose 2,6=bisphosphate
PFK27	ATP + beta=D=Fructose 6=phosphate => ADP + D=Fructose 2,6=bisphosphate
PGI1_1	alpha=D=Glucose 6=phosphate <=> beta=D=Fructose 6=phosphate
PGI1_2	alpha=D=Glucose 6=phosphate <=> beta=D=Glucose 6=phosphate
PGI1_3	beta=D=Glucose 6=phosphate <=> beta=D=Fructose 6=phosphate
PGK1	3=Phospho=D=glyceroyl phosphate + ADP <=> 3=Phospho=D=glycerate + ATP
PGM1_1	D=Ribose 1=phosphate <=> D=Ribose 5=phosphate
PGM1_2	D=Glucose 1=phosphate <=> alpha=D=Glucose 6=phosphate
PGM2_1	D=Ribose 1=phosphate <=> D=Ribose 5=phosphate
PGM2_2	D=Glucose 1=phosphate <=> alpha=D=Glucose 6=phosphate
PGS1	CDPdiacylglycerolM + sn=Glycerol 3=phosphateM <=> CMPM + PhosphatidylglycerophosphateM
PHA2	Prephenate => CO2 + Phenylpyruvate
PHO11	FMN => Riboflavin + Orthophosphate

PHO8	2=Amino=4=hydroxy=6=(erythro=1,2,3=trihydroxypropyl)=dihydropteridine triphosphate => 2=Amino=4=hydroxy=6=(D=erythro=1,2,3=trihydroxypropyl)=7,8=dihydropteridine + 3.000000 Orthophosphate
PHO84	Plxt + H+EXT <=> Orthophosphate
PHO90	Plxt + H+EXT <=> Orthophosphate
PHO91	Plxt + H+EXT <=> Orthophosphate
PIK1	ATP + 1=Phosphatidyl=D=myo=inositol => ADP + 1=Phosphatidyl=D=myo=inositol=4=phosphate
PIS1	CDPdiacylglycerol + myo=Inositol => CMP + 1=Phosphatidyl=D=myo=inositol
PLB1	Lysophosphatidylcholine => Glycerophosphatidylcholine + Acyl_acids
PLB2	Lysophosphatidylcholine => Glycerophosphatidylcholine + Acyl_acids
PLB3	Lysophosphatidylcholine => Glycerophosphatidylcholine + Acyl_acids
PLC1	1=Phosphatidyl=D=myo=inositol=4,5=bisphosphate => D=myo=inositol=1,4,5=trisphosphate + Diacylglycerol
PMA1	ATP => ADP + Orthophosphate + H+EXT
PMA2	ATP => ADP + Orthophosphate + H+EXT
PMI40	D=Mannose 6=phosphate <=> beta=D=Fructose 6=phosphate
PMP1	ATP => ADP + Orthophosphate + H+EXT
PMP2	ATP => ADP + Orthophosphate + H+EXT
PMT1	Dolichyl beta=D=mannosyl phosphate => Dolichyl phosphate + Mannan
PMT2	Dolichyl beta=D=mannosyl phosphate => Dolichyl phosphate + Mannan
PMT3	Dolichyl beta=D=mannosyl phosphate => Dolichyl phosphate + Mannan
PMT4	Dolichyl beta=D=mannosyl phosphate => Dolichyl phosphate + Mannan
PMT5	Dolichyl beta=D=mannosyl phosphate => Dolichyl phosphate + Mannan
PMT6	Dolichyl beta=D=mannosyl phosphate => Dolichyl phosphate + Mannan
PNC1_1	Nicotinamide <=> Nicotinate + NH3
PNC1_2	NicotinamideM <=> NicotinateM + NH3M
PNP1_1	Deoxyuridine + Orthophosphate <=> Uracil + Deoxy=ribose 1=phosphate
PNP1_10	AdenosineM + OrthophosphateM <=> AdenineM + alpha=D=Ribose 1=phosphateM
PNP1_11	GuanosineM + OrthophosphateM <=> GuanineM + alpha=D=Ribose 1=phosphateM
PNP1_2	Thymidine + Orthophosphate <=> Thymine + Deoxy=ribose 1=phosphate
PNP1_3	Deoxyinosine + Orthophosphate <=> HYXN + Deoxy=ribose 1=phosphate
PNP1_4	Deoxyadenosine + Orthophosphate <=> Adenine + Deoxy=ribose 1=phosphate
PNP1_5	Deoxyguanosine + Orthophosphate <=> Guanine + Deoxy=ribose 1=phosphate
PNP1_6	HYXN + D=Ribose 1=phosphate <=> Inosine + Orthophosphate
PNP1_7	Adenine + D=Ribose 1=phosphate <=> Orthophosphate + Adenosine
PNP1_8	Guanine + D=Ribose 1=phosphate <=> Orthophosphate + Guanosine
PNP1_9	Xanthine + D=Ribose 1=phosphate <=> Orthophosphate + Xanthosine

Pool_Acyl1	0.031552 Decanoyl=CoA + 0.029970 Dodecanoyl=CoA + 0.053571 Tetradecanoyl=CoA + 0.018042 Tetradecanoyl=9=ene=CoA + 0.188729 Hexadecanoyl=CoA + 0.476544 Hexadecanoyl=9=ene=CoA + 0.077912 Octadecanoyl
Pool_Acyl2	0.008290 Decanoyl=CoA + 0.008277 Dodecanoyl=CoA + 0.019114 Tetradecanoyl=CoA + 0.005707 Tetradecanoyl=9=ene=CoA + 0.154233 Hexadecanoyl=CoA + 0.604950 Hexadecanoyl=9=ene=CoA + 0.037531 Octadecanoyl
POS5_1	NAD+ + ATP => NADP+ + ADP
POS5_2	NAD+M + ATPM => NADP+M + ADPM
POT1_1	3=Keto=C18=CoA + CoA => Hexadecanoyl=CoA + Acetyl=CoA
POT1_2	3=Keto=C16=CoA + CoA => Tetradecanoyl=CoA + Acetyl=CoA
POT1_3	3=Keto=C14=CoA + CoA => Dodecanoyl=CoA + Acetyl=CoA
POT1_4	3=keto=Dodecanoyl=CoA + CoA => Acetyl=CoA + Decanoyl=CoA
POT1_5	3=keto=Decanoyl=CoA + CoA => Acetyl=CoA + Octanoyl=CoA
POT1_6	3=keto=Octanoyl=CoA + CoA => Acetyl=CoA + Hexanoyl=CoA
POT1_7	3=keto=Hexanoyl=CoA + CoA => Acetyl=CoA + Butanoyl=CoA
POT1_8	3=keto=Butanoyl=CoA + CoA => 2.000000 Acetyl=CoA
POX1_1	Octadecanoyl=CoA + Oxygen => H2O2 + Trans=2=C18=CoA
POX1_10	Octadecanoyl=9=ene=CoA + Oxygen => H2O2 + Trans=2=C181=CoA
POX1_11	Tetradecanoyl=9=ene=CoA + Oxygen => H2O2 + Trans=2=C141=CoA
POX1_12	FFA_diene_even=CoA + Oxygen => H2O2 + Trans=3=5=diene=CoA
POX1_2	Hexadecanoyl=CoA + Oxygen => H2O2 + Trans=2=C16=CoA
POX1_3	Tetradecanoyl=CoA + Oxygen => H2O2 + Trans=2=C14=CoA
POX1_4	Dodecanoyl=CoA + Oxygen => H2O2 + trans=delta2=Dodecaenoyl=CoA
POX1_5	Decanoyl=CoA + Oxygen => H2O2 + trans=delta2=Decaenoyl=CoA
POX1_6	Octanoyl=CoA + Oxygen => H2O2 + trans=delta2=Octaenoyl=CoA
POX1_7	Hexanoyl=CoA + Oxygen => H2O2 + trans=delta2=Hexaenoyl=CoA
POX1_8	Butanoyl=CoA + Oxygen => H2O2 + trans=delta2=Butaenoyl=CoA
POX1_9	Hexadecanoyl=9=ene=CoA + Oxygen => H2O2 + Trans=2=C161=CoA
PPA2	PyrophosphateM => 2.000000 OrthophosphateM
PPT2	CoA => Adenosine 3',5'=bisphosphate + Acyl=carrier protein
PRO1	L=Glutamate + ATP => ADP + alpha=D=Glutamyl phosphate
PRO2_1	alpha=D=Glutamyl phosphate + NADH => NAD+ + Orthophosphate + L=Glutamate 5=semialdehyde
PRO2_2	alpha=D=Glutamyl phosphate + NADPH => NADP+ + Orthophosphate + L=Glutamate 5=semialdehyde
PRO3_1	(S)=1=Pyrroline=5=carboxylate + NADPH => L=Proline + NADP+
PRO3_2	L=1=Pyrroline=3=hydroxy=5=carboxylate + NADPH => trans=4=Hydroxy=L=proline + NADP+
PRO3_3	L=1=Pyrroline=3=hydroxy=5=carboxylate + NADH => trans=4=Hydroxy=L=proline + NAD+
PRS1	D=Ribose 5=phosphate + ATP <=> 5=Phospho=alpha=D=ribose 1=diphosphate + AMP
PRS2	D=Ribose 5=phosphate + ATP <=> 5=Phospho=alpha=D=ribose 1=diphosphate + AMP

PRS3	D=Ribose 5=phosphate + ATP <=> 5=Phospho=alpha=D=ribose 1=diphosphate + AMP
PRS4	D=Ribose 5=phosphate + ATP <=> 5=Phospho=alpha=D=ribose 1=diphosphate + AMP
PRS5	D=Ribose 5=phosphate + ATP <=> 5=Phospho=alpha=D=ribose 1=diphosphate + AMP
PSA1	GTP + alpha=D=Mannose 1=phosphate => Pyrophosphate + GDPmannose
PSD1	PhosphatidylserineM => PhosphatidylethanolamineM + CO2M
PSD2	Phosphatidylserine => Phosphatidylethanolamine + CO2
PTR2_1	DIPEPxt + H+EXT => Dipeptide
PTR2_2	OPEPxt + H+EXT => Oligopeptide
PTR2_3	PEPTxt + H+EXT => Peptide
PUR5	IMP + NAD+ => NADH + Xanthosine 5'=phosphate
PUS1	Uracil + D=Ribose 5=phosphate <=> Pseudouridine 5'=phosphate
PUS2	Uracil + D=Ribose 5=phosphate <=> Pseudouridine 5'=phosphate
PUS4	Uracil + D=Ribose 5=phosphate <=> Pseudouridine 5'=phosphate
PUT1	L=ProlineM + NAD+M => (S)=1=Pyrroline=5=carboxylateM + NADHM
PUT2	L=Glutamate 5=semialdehydeM + NADP+M => NADPHM + GlutamateM
PUT4_1	ALAxt + H+EXT <=> L=Alanine
PUT4_2	GLYxt + H+EXT <=> Glycine
PUT4_3	PROxt + H+EXT <=> L=Proline
PUT4_4	GABAxt + H+EXT => 4=Aminobutanoate
PXA1/PXA2_1	Dodecanoyl=CoA <=> Dodecanoyl=CoA
PXA1/PXA2_2	Tetradecanoyl=CoA <=> Tetradecanoyl=CoA
PXA1/PXA2_3	Decanoyl=CoA <=> Decanoyl=CoA
PXA1/PXA2_4	Hexadecanoyl=CoA <=> Hexadecanoyl=CoA
PXA1/PXA2_5	Octadecanoyl=CoA <=> Octadecanoyl=CoA
PXA1/PXA2_6	Hexadecanoyl=9=ene=CoA <=> Hexadecanoyl=9=ene=CoA
PXA1/PXA2_7	Octadecanoyl=9=ene=CoA <=> Octadecanoyl=9=ene=CoA
PXA1/PXA2_8	Tetradecanoyl=9=ene=CoA <=> Tetradecanoyl=9=ene=CoA
PYC1	Pyruvate + ATP + CO2 => ADP + Oxaloacetate + Orthophosphate
PYC2	Pyruvate + ATP + CO2 => ADP + Oxaloacetate + Orthophosphate
PYK2	Phosphoenolpyruvate + ADP => Pyruvate + ATP
PYRD	(S)=Dihydroorotate + Ubiquinone=9M <=> UbiquinolM + Orotate
QNS1_1	Deamino=NAD+ + ATP + NH3 => NAD+ + AMP + Pyrophosphate
QNS1_2	Deamino=NAD+M + ATPM + NH3M => NAD+M + AMPM + PyrophosphateM
QPT1_1	Pyridine=2,3=dicarboxylate + 5=Phospho=alpha=D=ribose 1=diphosphate => Nicotinate D=ribonucleotide + CO2 + Pyrophosphate

QPT1_2	Pyridine=2,3=dicarboxylateM + 5=Phospho=alpha=D=ribose 1=diphosphateM => Nicotinate D=ribonucleotideM + CO2M + PyrophosphateM
QRI1	UTP + N=Acetyl=D=glucosamine 1=phosphate <=> UDP=N=acetyl=D=galactosamine + Pyrophosphate
RAM1	4=Hydroxybenzoate + all=trans=Nonaprenyl diphosphate => 3=Nonaprenyl=4=hydroxybenzoate + Pyrophosphate
RBK1_1	D=Ribose + ATP => D=Ribose 5=phosphate + ADP
RBK1_2	Deoxyribose + ATP => 2=Deoxy=D=ribose 5=phosphate + ADP
RER2	4=Hydroxybenzoate + all=trans=Nonaprenyl diphosphate => 3=Nonaprenyl=4=hydroxybenzoate + Pyrophosphate
RHR2	sn=Glycerol 3=phosphate => Glycerol + Orthophosphate
RIB1	GTP => 2,5=Diamino=6=hydroxy=4=(5'=phosphoribosylamino)=pyrimidine + Formate + Pyrophosphate
RIB5	L=3,4=Dihydroxy=2=butanone 4=phosphate + 5=Amino=6=ribitylamino=2,4 (1H, 3H)=pyrimidinedione => 6,7=Dimethyl=8=(1=D=ribityl)lumazine + Orthophosphate
RIB7_1	2,5=Diamino=6=hydroxy=4=(5'=phosphoribosylamino)=pyrimidine => 5=Amino=6=(5'=phosphoribosylamino)uracil + NH3
RIB7_2	5=Amino=6=(5'=phosphoribosylamino)uracil + NADPH => 5=Amino=6=(5'=phosphoribitylamino)uracil + NADP+
RIP1	UbiquinolM + 2.000000 Ferricytochrome cM + 1.500000 H+M => Ubiquinone=9M + 2.000000 Ferrocytochrome cM
RKI1	D=Ribulose 5=phosphate <=> D=Ribose 5=phosphate
RMA1	Tetrahydrofolate + ATP + L=Glutamate <=> ADP + Orthophosphate + Tetrahydrofolyl=[Glu](n)
RNR1_1	ADP + Reduced thioredoxin => dADP + Oxidized thioredoxin
RNR1_2	GDP + Reduced thioredoxin => dGDP + Oxidized thioredoxin
RNR1_3	CDP + Reduced thioredoxin => dCDP + Oxidized thioredoxin
RNR1_4	UDP + Reduced thioredoxin => Oxidized thioredoxin + dUDP
RNR3	ADP + Reduced thioredoxin => dADP + Oxidized thioredoxin
RPE1	D=Ribulose 5=phosphate <=> D=Xylulose 5=phosphate
SAC1_1	1=Phosphatidyl=D=myo=inositol=3=phosphate => 1=Phosphatidyl=D=myo=inositol + Pyrophosphate
SAC1_2	1=Phosphatidyl=D=myo=inositol=4=phosphate => 1=Phosphatidyl=D=myo=inositol + Pyrophosphate
SAC1_3	1=Phosphatidyl=D=myo=inositol=3,5=bisphosphate => 1=Phosphatidyl=D=myo=inositol=3=phosphate + Pyrophosphate
SAH1	S=Adenosyl=L=homocysteine => Homocysteine + Adenosine
SAM1	L=Methionine + ATP => Pyrophosphate + Orthophosphate + S=Adenosyl=L=methionine
SAM2	L=Methionine + ATP => Pyrophosphate + Orthophosphate + S=Adenosyl=L=methionine
SAM3	SAMxt + H+EXT => S=Adenosyl=L=methionine
SAM4	S=Adenosyl=L=methionine + Homocysteine => S=Adenosyl=L=homocysteine + L=Methionine
SCS7	P=Ceramide + NADH + Oxygen => D=Ceramide + NADP+
SCT1_1	sn=Glycerol 3=phosphate + AcylCoAs => Acyl=sn=glycerol 3=phosphate + CoA
SCT1_2	Glycerone phosphate + AcylCoAs => Acyldihydroxyacetone phosphate + CoA
SDH3_1	SuccinateM + FADM <=> FumarateM + FADH2M
SDH3_2	FADH2M + Ubiquinone=9M <=> FADM + UbiquinolM
SEC53	D=Mannose 6=phosphate <=> alpha=D=Mannose 1=phosphate

SEC59	CTP + Dolichol => CDP + Dolichyl phosphate
SER1_1	3=Phosphonooxypyruvate + L=Glutamate => 2=Oxoglutarate + 3=Phosphoserine
SER1_2	2=Oxo=3=hydroxy=4=phosphobutanoate + L=Glutamate <=> O=Phospho=4=hydroxy=L=threonine + 2=Oxoglutarate
SER2	3=Phosphoserine => Orthophosphate + L=Serine
SER3	3=Phospho=D=glycerate + NAD+ => NADH + 3=Phosphonooxypyruvate
SER33	3=Phospho=D=glycerate + NAD+ => NADH + 3=Phosphonooxypyruvate
SES1	ATP + L=Serine + tRNA(Ser) => AMP + Pyrophosphate + L=Serinyl=tRNA(Ser)
SFA1_1	Formaldehyde + Glutathione + NAD+ <=> S=Formylglutathione + NADH
SFA1_2	Ethanol + NAD+ <=> Acetaldehyde + NADH
SFC1	Succinate + FumarateM => SuccinateM + Fumarate
SHM1	TetrahydrofolateM + L=SerineM <=> GlycineM + 5,10=MethylenetetrahydrofolateM
SHM2	Tetrahydrofolate + L=Serine <=> Glycine + 5,10=Methylenetetrahydrofolate
SLC1	Acyl=sn=glycerol 3=phosphate + AcylCoAs => Phosphatidate + CoA
SOL1	D=Glucono=1,5=lactone 6=phosphate => 6=Phospho=D=gluconate
SOL2	D=Glucono=1,5=lactone 6=phosphate => 6=Phospho=D=gluconate
SOL3	D=Glucono=1,5=lactone 6=phosphate => 6=Phospho=D=gluconate
SOL4	D=Glucono=1,5=lactone 6=phosphate => 6=Phospho=D=gluconate
SOR1	D=Sorbitol + NAD+ => D=Fructose + NADH
SPE1	L=Ornithine => Putrescine + CO2
SPE2	S=Adenosyl=L=methionine <=> S=Adenosylmethioninamine + CO2
SPE3	Putrescine + S=Adenosyl=L=methionine => Spermidine + 5'=Methylthioadenosine
SPE4	S=Adenosylmethioninamine + Spermidine => 5'=Methylthioadenosine + Spermine
SPO14	Phosphatidylcholine => Phosphatidate + Choline
SPO22	2.000000 Phosphatidylcholine => Glycerophosphatidylcholine + Acyl_acids + Lysophosphatidylcholine
SPR1	1,3=beta=D=Glucan => alpha=D=Glucose
SPS19_1	Trans=2=C161=CoA => Trans=3=C16=CoA
SPS19_2	Trans=2=C181=CoA => Trans=3=C18=CoA
SPS19_3	Trans=2=C141=CoA => Trans=3=C14=CoA
SPS19_4	Trans=2=4=diene=CoA => Trans=3=C16=CoA
SPS19_5	Trans=2=4=diene=CoA => Trans=3=C18=CoA
SPS19_6	Trans=2=4=diene=CoA => Trans=3=C14=CoA
SRT1	4=Hydroxybenzoate + all=trans=Nonaprenyl diphosphate => 3=Nonaprenyl=4=hydroxybenzoate + Pyrophosphate
STL1_1	GLCxt => alpha=D=Glucose
STL1_2	GLACxt => D=Galactose

STL1_3	GLUxt <=> L=Glutamate
STL1_4	GLUxt <=> L=Glutamate
STT4	ATP + 1=Phosphatidyl=D=myo=inositol => ADP + 1=Phosphatidyl=D=myo=inositol=4=phosphate
SUC2	SUCxt => GLCxt + FRUxt
SUL1	SLFxt => Sulfate
SUL2	SLFxt => Sulfate
SUR1_1	Inositol=phosphoryl=D=ceramide + GDPmannose => Mannose=inositol=P=D=ceramide
SUR1_2	Inositol=phosphoryl=P=ceramide + GDPmannose => Mannose=inositol=P=P=ceramide
SUR2	Sphinganine + Oxygen + NADPH => Phytosphingosine + NADP+
TAL1	D=Glyceraldehyde 3=phosphate + Sedoheptulose 7=phosphate <=> D=Erythrose 4=phosphate + beta=D=Fructose 6=phosphate
TAT1_1	ILExt + H+EXT <=> L=Isoleucine
TAT1_2	LEUxt + H+EXT <=> L=Leucine
TAT1_3	TRPxt + H+EXT <=> L=Tryptophan
TAT1_4	TYRxt + H+EXT <=> L=Tyrosine
TAT1_5	THRxt + H+EXT <=> L=Threonine
TAT2_1	ALAxt + H+EXT <=> L=Alanine
TAT2_2	CYSxt + H+EXT <=> L=Cysteine
TAT2_3	GLYxt + H+EXT <=> Glycine
TAT2_4	PHExt + H+EXT <=> L=Phenylalanine
TAT2_5	TRPxt + H+EXT <=> L=Tryptophan
TAT2_6	TYRxt + H+EXT <=> L=Tyrosine
TDH1	D=Glyceraldehyde 3=phosphate + Orthophosphate + NAD+ <=> NADH + 3=Phospho=D=glyceroyl phosphate
TDH2	D=Glyceraldehyde 3=phosphate + Orthophosphate + NAD+ <=> NADH + 3=Phospho=D=glyceroyl phosphate
TDH3	D=Glyceraldehyde 3=phosphate + Orthophosphate + NAD+ <=> NADH + 3=Phospho=D=glyceroyl phosphate
TES1	AcylCoAs => Acyl_acids + CoA
TGL1	Ergosterol=ester => Ergosterol + Acyl_acids
TGL2	Triacylglycerol => Diacylglycerol + Acyl_acids
TGL3	Triacylglycerol => Diacylglycerol + Acyl_acids
TGL4	Triacylglycerol => Diacylglycerol + Acyl_acids
TGL5	Triacylglycerol => Diacylglycerol + Acyl_acids
THI20_1	4=Amino=5=hydroxymethyl=2=methylpyrimidine + ATP => 4=Amino=2=methyl=5=phosphomethylpyrimidine + ADP
THI20_2	4=Amino=2=methyl=5=phosphomethylpyrimidine + ATP => 2=Methyl=4=amino=5=hydroxymethylpyrimidine diphosphate + ADP
THI21	4=Amino=5=hydroxymethyl=2=methylpyrimidine + ATP => 4=Amino=2=methyl=5=phosphomethylpyrimidine + ADP
THI22	4=Amino=5=hydroxymethyl=2=methylpyrimidine + ATP => 4=Amino=2=methyl=5=phosphomethylpyrimidine + ADP

THI6_1	5=(2=Hydroxyethyl)=4=methylthiazole + ATP => 4=Methyl=5=(2=phosphoethyl)=thiazole + ADP
THI6_2	4=Methyl=5=(2=phosphoethyl)=thiazole + 2=Methyl=4=amino=5=hydroxymethylpyrimidine diphosphate => Thiamin monophosphate + Pyrophosphate
THI7	THMxt + H+EXT => Thiamin
THI80_1	ATP + Thiamin => AMP + Thiamine diphosphatee
THI80_2	ATP + Thiamine diphosphatee => AMP + Thiamin triphosphate
THR1	L=Homoserine + ATP => ADP + O=Phospho=L=homoserine
THR4_1	O=Phospho=L=homoserine => Orthophosphate + L=Threonine
THR4_2	O=Phospho=4=hydroxy=L=threonine => 4=Hydroxy=L=threonine + Orthophosphate
THS1	ATP + L=Threonine + tRNA(Thr) => AMP + Pyrophosphate + L=Threonyl=tRNA(Thr)
TKL1_1	D=Ribose 5=phosphate + D=Xylulose 5=phosphate <=> D=Glyceraldehyde 3=phosphate + Sedoheptulose 7=phosphate
TKL1_2	D=Xylulose 5=phosphate + D=Erythrose 4=phosphate <=> beta=D=Fructose 6=phosphate + D=Glyceraldehyde 3=phosphate
TKL2_1	D=Ribose 5=phosphate + D=Xylulose 5=phosphate <=> D=Glyceraldehyde 3=phosphate + Sedoheptulose 7=phosphate
TKL2_2	D=Xylose 5=phosphate + D=Erythrose 4=phosphate <=> beta=D=Fructose 6=phosphate + D=Glyceraldehyde 3=phosphate
TOR1	ATP + 1=Phosphatidyl=D=myo=inositol => ADP + 1=Phosphatidyl=D=myo=inositol=3=phosphate
TOR2	ATP + 1=Phosphatidyl=D=myo=inositol => ADP + 1=Phosphatidyl=D=myo=inositol=3=phosphate
TPC1	Thiamine diphosphate => Thiamine diphosphateM
TPI1	Glycerone phosphate <=> D=Glyceraldehyde 3=phosphate
TPN1	VB6xt => Pyridoxine
TPS1	UDPglucose + alpha=D=Glucose 6=phosphate => UDP + alpha,alpha'=Trehalose 6=phosphate
TPS2	alpha,alpha'=Trehalose 6=phosphate => alpha,alpha=Trehalose + Orthophosphate
TPS3	UDPglucose + alpha=D=Glucose 6=phosphate => UDP + alpha,alpha'=Trehalose 6=phosphate
TRK1	Kxt + H+EXT <=> Potassium
TRP1	N=(5=Phospho=D=ribosyl)anthranilate => 1=(2=Carboxyphenylamino)=1=deoxy=D=ribulose 5=phosphate
TRP2_1	Chorismate + L=Glutamine => L=Glutamate + Pyruvate + Anthranilate
TRP2_2	Chorismate => 4=Hydroxybenzoate + Pyruvate
TRP3_1	Chorismate + L=Glutamine => L=Glutamate + Pyruvate + Anthranilate
TRP3_2	1=(2=Carboxyphenylamino)=1=deoxy=D=ribulose 5=phosphate => CO2 + Indoleglycerol phosphate
TRP3_3	Chorismate => 4=Hydroxybenzoate + Pyruvate
TRP4	Anthranilate + 5=Phospho=alpha=D=ribose 1=diphosphate => Pyrophosphate + N=(5=Phospho=D=ribosyl)anthranilate
TRP5	Indoleglycerol phosphate + L=Serine => D=Glyceraldehyde 3=phosphate + L=Tryptophan
TRR1	Oxidized thioredoxin + NADPH => NADP+ + Reduced thioredoxin
TRR2	Oxidized thioredoxinM + NADPHM => NADP+M + Reduced thioredoxinM
TSC10	3=Dehydrosphinganine + NADPH => Sphinganine + NADP+
TSC13_1	Trans=2=C14=CoA + NADPH => Tetradecanoyl=CoA + NADP+

TSC13_2	Trans=2=C16=CoA + NADPH => Hexadecanoyl=CoA + NADP+
TSC13_3	Trans=2=C18=CoA + NADPH => Octadecanoyl=CoA + NADP+
TSC13_4	Trans=2=C20=CoA + NADPH => C20=CoA + NADP+
TSC13_5	Trans=2=C22=CoA + NADPH => C22=CoA + NADP+
TSC13_6	Trans=2=C24=CoA + NADPH => C24=CoA + NADP+
TSC13_7	Trans=2=C26=CoA + NADPH => C26=CoA + NADP+
TSL1	UDPglucose + alpha=D=Glucose 6=phosphate => UDP + alpha,alpha'=Trehalose 6=phosphate
TYR1	Prephenate + NADP+ => 3=(4=Hydroxyphenyl)pyruvate + CO2 + NADPH
TYS1	ATP + L=Tyrosine + tRNA(Tyr) => AMP + Pyrophosphate + L=Tyrosyl=tRNA(Tyr)
U1_	D=Fructose 1=phosphate + ATP => beta=D=Fructose 1,6=bisphosphate + ADP
U100_	a=Iminosuccinate + Glycerone phosphate => Orthophosphate + Pyridine=2,3=dicarboxylate
U101_	NADP+ => NAD+ + Orthophosphate
U102_	NAD+ => Nicotinamide + ADPribose
U103_	Adenosine + Orthophosphate <=> Adenine + RIP
U104_	Guanosine + Orthophosphate <=> Guanine + RIP
U105_	NADP+M => NAD+M + OrthophosphateM
U106_	NAD+M => NicotinamideM + ADPriboseM
U107_	2=Nonaprenylphenol + Oxygen => 2=Nonaprenyl=6=hydroxyphenol
U108_	2=Nonaprenyl=6=methoxyphenolM + OxygenM => 2=Nonaprenyl=6=methoxy=1,4=benzoquinoneM
U109_	Oxygen <=> OxygenM
U110_	CO2 <=> CO2M
U111_	Ethanol <=> EthanolM
U112_	NH3 <=> NH3M
U113_	Methane <=> MethaneM
U114_	TetrahydrofolateM <=> Tetrahydrofolate
U115_	5,10=MethylenetetrahydrofolateM <=> 5,10=Methylenetetrahydrofolate
U116_	L=SerineM <=> L=Serine
U117_	GlycineM <=> Glycine
U118_	3=IsopropylmalateM <=> 3=Isopropylmalate
U119_	3=Carboxy=4=methyl=2=oxopentanoateM <=> 3=Carboxy=4=methyl=2=oxopentanoate
U120_	L=ProlineM <=> L=Proline
U121_	CMPM <=> CMP
U122_	AcetateM <=> Acetate
U123_	O=Acetylcarnitine => O=AcetylcarnitineM

U124_	CarnitineM => Carnitine
U125_	2=Acetolactate <=> 2=AcetolactateM
U126_	Acetoacetate <=> AcetoacetateM
U127_	Sulfate => SulfateM + H+M
U128_	L=ThreonineM <=> L=Threonine
U129_	2=OxoadipateM => 2=Oxoadipate
U130_	Malonate + OrthophosphateM <=> MalonateM + Orthophosphate
U131_	2=Isopropylmalate <=> 2=IsopropylmalateM
U132_	(R)=Lactate <=> (R)=LactateM + H+M
U133_	Pyruvate <=> PyruvateM + H+M
U135_	L=Glutamate + HO=M => GlutamateM
U136_	(R)=2=Oxoisovalerate <=> (R)=2=OxoisovalerateM
U137_	3=Methyl=2=oxobutanoate <=> 3=Methyl=2=oxobutanoateM
U138_	Riboflavin <=> RiboflavinM
U139_	Dethiobiotin <=> DethiobiotinM
U140_	(S)=3=Hydroxy=3=methylglutaryl=CoA <=> (S)=3=Hydroxy=3=methylglutaryl=CoAM
U141_	(R)=Mevalonate <=> (R)=MevalonateM
U142_	Phosphatidate <=> PhosphatidateM
U143_	Pantetheine 4'=phosphate <=> Pantetheine 4'=phosphateM
U144_	Adenine <=> AdenineM
U145_	5=Phospho=alpha=D=ribose 1=diphosphate <=> 5=Phospho=alpha=D=ribose 1=diphosphateM
U146_	Dihydrofolate <=> DihydrofolateM
U147_	Pyridine=2,3=dicarboxylate <=> Pyridine=2,3=dicarboxylateM
U148_	OPP <=> OPPm
U149_	S=Adenosyl=L=methionine <=> S=Adenosyl=L=methionineM
U15_	Deoxyuridine + ATP => dUMP + ADP
U150_	S=Adenosyl=L=homocysteine <=> S=Adenosyl=L=homocysteineM
U151_	Glycerone phosphateM => Glycerone phosphate
U152_	sn=Glycerol 3=phosphate => sn=Glycerol 3=phosphateM
U154_	SUCxt + H+EXT => Sucrose
U155_	MALxt + 2=Oxoglutarate <=> Malate + AKGxt
U156_	AMGxt <=> Methyl=D=glucoside
U157_	SORxt <=> Sorbose
U158_	ARABxt <=> D=Arabinose

U159_	FUCxt + H+EXT <=> beta=D=Fucose
U16_	Thymidine + ATP => ADP + dTMP
U160_	GLTLxt + H+EXT => GLTL
U161_	GLTxt + H+EXT => L=Glucitol
U162_	GLAMxt + H+EXT <=> Glucosamine
U163_	MNTxt + H+EXT => D=Mannitol
U164_	MELIxt + H+EXT => Melibiose
U165_	NAGxt + H+EXT => N=Acetylglucosamine
U166_	RMNxt + H+EXT => D=Rhamnose
U167_	RIBxt + H+EXT => D=Ribose
U168_	TRExt + H+EXT => alpha,alpha=Trehalose
U17_	dCMP + ATP <=> ADP + dCDP
U170_	XYLxt <=> D=Xylose
U171_	PTRSCxt + H+EXT => Putrescine
U172_	SPRMDxt + H+EXT => Spermidine
U173_	NMNxt + H+EXT => NMN
U174_	ADNxt + H+EXT => Adenosine
U175_	GSNxt + H+EXT => Guanosine
U176_	CYTDxt + H+EXT => Cytidine
U177_	INSxt + H+EXT => Inosine
U178_	XTSINExt + H+EXT => Xanthosine
U179_	DTxt + H+EXT => Thymidine
U18_	CMP + ATP <=> ADP + CDP
U180_	DINxt + H+EXT => Deoxyinosine
U181_	DGxt + H+EXT => Deoxyguanosine
U182_	DAxt + H+EXT => Deoxyadenosine
U183_	DCxt + H+EXT => Deoxycytidine
U184_	DUxt + H+EXT => Deoxyuridine
U185_	ADNxt + H+EXT => Adenosine
U186_	CYTDxt + H+EXT => Cytidine
U187_	DTxt + H+EXT => Thymidine
U188_	DAxt + H+EXT => Deoxyadenosine
U189_	DCxt + H+EXT => Deoxycytidine
U19_	dAMP + ATP <=> dADP + ADP

U190_	DUxt + H+EXT => Deoxyuridine
U191_	ADNxt + H+EXT => Adenosine
U192_	GSNxt + H+EXT => Guanosine
U193_	CYTDxt + H+EXT => Cytidine
U194_	INSxt + H+EXT => Inosine
U195_	DTxt + H+EXT => Thymidine
U196_	DINxt + H+EXT => Deoxyinosine
U197_	DGxt + H+EXT => Deoxyguanosine
U198_	DAxt + H+EXT => Deoxyadenosine
U199_	DCxt + H+EXT => Deoxycytidine
U2_	Formate + Ubiquinone=9M => UbiquinolM + CO2 + 2 HEXT
U20_	Inosine + ATP => IMP + ADP
U200_	DUxt + H+EXT => Deoxyuridine
U201_	HYXNxt + H+EXT <=> HYXN
U202_	XANxt <=> Xanthine
U203_	FORxt <=> Formate
U204_	ETHxt <=> Ethanol
U205_	SUCCxt + H+EXT <=> Succinate
U206_	CITxt + H+EXT <=> Citrate
U207_	FUMxt + H+EXT <=> Fumarate
U208_	C140xt => Tetradecanoic_acid
U2081_	C141xt => Tetradecanoyl=9=ene_acid
U209_	C160xt => Hexadecanoic_acid
U21_	Guanosine + ATP => GMP + ADP
U210_	C161xt => Hexadecanoyl=9=ene_acid
U211_	C180xt => Octadecanoic_acid
U212_	C181xt => Octadecanoyl=9=ene_acid
U213_	AKGxt + H+EXT <=> 2=Oxoglutarate
U214_	ATP => ADP + Orthophosphate
U215_	GLALxt <=> Glycolaldehyde
U216_	ACALxt <=> Acetaldehyde
U217_	MTHNxt <=> Methane
U218_	PAPxt <=> Adenosine 3',5'=bisphosphate
U219_	DTTPxt <=> dTTP

U22_	Uridine + Orthophosphate <=> Uracil + D=Ribose 1=phosphate
U220_	THYxt <=> Thymine + H+EXT
U221_	GA6Pxt <=> D=Glucosamine 6=phosphate
U222_	AONAxt + H+EXT <=> 8=Amino=7=oxononanoate
U223_	DANNAxt + H+EXT <=> 7,8=Diaminononanoate
U224_	OGTxt => Oxidized glutathione
U225_	SPRMxt => Spermine
U226_	PIMExt => Pimelic Acid
U227_	O2xt <=> Oxygen
U228_	CO2xt <=> CO2
U229_	RFLAVxt + H+EXT => Riboflavin
U23_	CMP => Cytosine + D=Ribose 5=phosphate
U24_	dUMP => Deoxyuridine + Orthophosphate
U25_	dTMP => Thymidine + Orthophosphate
U26_	dAMP => Deoxyadenosine + Orthophosphate
U27_	dGMP => Deoxyguanosine + Orthophosphate
U28_	dCMP => Deoxycytidine + Orthophosphate
U29_	CMP => Cytidine + Orthophosphate
U30_	AMP => Orthophosphate + Adenosine
U31_	GMP => Orthophosphate + Guanosine
U32_	IMP => Orthophosphate + Inosine
U33_	Xanthosine 5'=phosphate => Orthophosphate + Xanthosine
U34_	UMP => Orthophosphate + Uridine
U35_	ATP + Reduced thioredoxin => dATP + Oxidized thioredoxin
U36_	GTP + Reduced thioredoxin => dGTP + Oxidized thioredoxin
U37_	CTP + Reduced thioredoxin => dCTP + Oxidized thioredoxin
U38_	UTP + Reduced thioredoxin => Oxidized thioredoxin + dUTP
U39_	GTP => Guanosine + 3.000000 Orthophosphate
U40_	dGTP => Deoxyguanosine + 3.000000 Orthophosphate
U41_	(S)=1=Pyrroline=5=carboxylateM + NAD+M => NADHM + GlutamateM
U42_	L=Glutamine => L=Glutamate + NH3
U43_	L=Glutamine => L=Glutamate + NH3
U44_	D=Glucosamine 6=phosphate => beta=D=Fructose 6=phosphate + NH3
U45_	D=Mannitol 1=phosphate + NAD+ <=> beta=D=Fructose 6=phosphate + NADH

U46_	L=Threonine + NAD+ => Glycine + Acetate + NADH
U47_	Homocysteine + 5=Methyltetrahydrofolate => Tetrahydrofolate + L=Methionine
U48_	L=Serine + Acetyl=CoA => CoA + O=Acetyl=L=serine
U49_	2=Hydroxybutane=1,2,4=tricarboxylateM <=> But=1=ene=1,2,4=tricarboxylateM
U50_	OxaloglutarateM <=> CO2M + 2=OxoadipateM
U51_	2=Oxoadipate + L=Glutamate <=> L=2=Aminoadipate + 2=Oxoglutarate
U52_	Prephenate + NAD+ => 3=(4=Hydroxyphenyl)pyruvate + CO2 + NADH
U53_	L=Formylkynurenine => Formate + L=Kynurenine
U54_	2=Amino=3=carboxymuconate semialdehyde => CO2 + 2=Aminomuconate 6=semialdehyde
U55_	2=Aminomuconate 6=semialdehyde + NAD+ => 2=Aminomuconate + NADH
U56_	2=Aminomuconate + NADPH => 2=Oxoadipate + NADP+ + NH3
U57_	3=(4=Hydroxyphenyl)pyruvate + Oxygen => Homogentisate + CO2
U58_	Homogentisate + Oxygen => 4=Maleylacetoacetate
U59_	4=Maleylacetoacetate => 4=Fumarylacetoacetate
U60_	4=Fumarylacetoacetate => Fumarate + Acetoacetate
U61_	Spermidine + Acetyl=CoA => N1=Acetylspermidine + CoA
U62_	N1=Acetylspermidine + Oxygen => N=Acetylputrescine + 3=Aminopropanal + H2O2
U63_	N=Acetylputrescine + Oxygen => 4=Aminobutyraldehyde + 3=Aminopropanal + H2O2
U64_	Spermine + Acetyl=CoA => N1=Acetylspermine + CoA
U65_	N1=Acetylspermine + Oxygen => N1=Acetylspermidine + 3=Aminopropanal + H2O2
U66_	L=Glutamate 5=semialdehyde <=> (S)=1=Pyrroline=5=carboxylate
U67_	L=Glutamate 5=semialdehydeM <=> (S)=1=Pyrroline=5=carboxylateM
U68_	4=AminobutyraldehydeM + NAD+M => 4=AminobutanoateM + NADHM
U70_	Aminoimidazole ribotide => 4=Amino=5=hydroxymethyl=2=methylpyrimidine
U71_	D=Glyceraldehyde 3=phosphate + Pyruvate => 1=Deoxy=d=threo=2=pentulose
U72_	1=Deoxy=d=threo=2=pentulose + L=Tyrosine + L=Cysteine => 5=(2=Hydroxyethyl)=4=methylthiazole + 4=Hydroxy=benzyl alcohol + CO2
U73_	1=Deoxy=d=threo=2=pentulose + L=Tyrosine + L=Cysteine => 5=(2=Hydroxyethyl)=4=methylthiazole + 4=Hydroxy=benzyl alcohol + CO2
U74_	1=Deoxy=d=threo=2=pentulose + L=Tyrosine + L=Cysteine => 5=(2=Hydroxyethyl)=4=methylthiazole + 4=Hydroxy=benzyl alcohol + CO2
U75_	1=Deoxy=d=threo=2=pentulose + L=Tyrosine + L=Cysteine => 5=(2=Hydroxyethyl)=4=methylthiazole + 4=Hydroxy=benzyl alcohol + CO2
U76_	Thiamin monophosphate + ATP <=> Thiamine diphosphatee + ADP
U77_	Thiamin monophosphate => Thiamin + Orthophosphate
U78_	5=Amino=6=(5'=phosphoribitylamino)uracil => 5=Amino=6=ribitylamino=2,4 (1H, 3H)=pyrimidinedione + Orthophosphate
U79_	D=Ribulose 5=phosphate => L=3,4=Dihydroxy=2=butanone 4=phosphate + Formate
U80_	FMNM + ATPM => FADM + PyrophosphateM

U81_	Pyridoxine + ATP => Pyridoxine phosphate + ADP
U82_	Pyridoxamine + ATP => Pyridoxamine phosphate + ADP
U83_	Pyridoxal + ATP => Pyridoxal phosphate + ADP
U84_	Pyridoxamine phosphate => Pyridoxamine + Orthophosphate
U85_	3.000000 Malonyl=CoA => 6=Carboxyhexanoyl=CoA + 2.000000 CoA + 2.000000 CO2
U86_	L=Alanine + 6=Carboxyhexanoyl=CoA <=> CO2 + CoA + 8=Amino=7=oxononanoate
U87_	2=Amino=4=hydroxy=6=(erythro=1,2,3=trihydroxypropyl)=dihydropteridine triphosphate => Pyrophosphate + Dihydroneopterin phosphate
U88_	4=amino=4=deoxychorismate => Pyruvate + 4=Aminobenzoate
U89_	Dihydropteroate + ATP + L=Glutamate => ADP + Orthophosphate + Dihydrofolate
U9_	PhosphatidylglycerophosphateM => OrthophosphateM + PhosphatidylglycerolM
U90_	ATPM + 10=FormyltetrahydrofolateM => ADPM + OrthophosphateM + 5=MethyltetrahydrofolateM
U91_	ATP + 10=Formyltetrahydrofolate => ADP + Orthophosphate + 5=Methyltetrahydrofolate
U92_	D=4'=Phosphopantothenate + CTP + L=Cysteine => CMP + Pyrophosphate + (R)=4'=Phosphopantothenoyl=L=cysteine
U93_	(R)=4'=Phosphopantothenoyl=L=cysteine => CO2 + Pantetheine 4'=phosphate
U94_	Pantetheine 4'=phosphate + ATP => Pyrophosphate + Dephospho=CoA
U95_	Pantetheine 4'=phosphateM + ATPM => PyrophosphateM + Dephospho=CoAM
U96_	Dephospho=CoA + ATP => ADP + CoA
U97_	Dephospho=CoAM + ATPM => ADPM + CoAM
U98_	L=Aspartate => CO2 + beta=Alanine
U99_	L=Aspartate + FADM => FADH2M + a=Iminosuccinate
UAcyl_1_1	Acetyl=[acyl=carrier protein] <=> Acetyl=[acyl=carrier protein]M
UAcyl_2_1	Butyryl=ACP <=> Butyryl=ACPM
UAcyl_3_1	Hexanoyl=ACP <=> Hexanoyl=ACPM
UAcyl_4_1	Octanoyl=ACP <=> Octanoyl=ACPM
UAcyl_5_1	Decanoyl=ACP <=> Decanoyl=ACPM
UAcyl_6_1	Dodecanoyl=ACP <=> Dodecanoyl=ACPM
UAcyl_7_1	Tetradecanoyl=ACP <=> Tetradecanoyl=ACPM
UAcyl_8_1	Hexadecanoyl=ACP <=> Hexadecanoyl=ACPM
UAcyl_8_2	Octadecanoyl=ACPM <=> Octadecanoyl=ACP
Uc10_	C100xt => Decanoic_acid
Uc12_	C120xt => Dodecanoic_acid
Uc24_	C24xt => C24_acid
Uc26_	C26xt => C26_acid
Uelo_1	3=Hydroxy=C14=CoA => Trans=2=C14=CoA

Uelo_2	3=Hydroxy=C16=CoA => Trans=2=C16=CoA
Uelo_3	3=Hydroxy=C18=CoA => Trans=2=C18=CoA
Uelo_4	3=Hydroxy=C20=CoA => Trans=2=C20=CoA
Uelo_5	3=Hydroxy=C22=CoA => Trans=2=C22=CoA
Uelo_6	3=Hydroxy=C24=CoA => Trans=2=C24=CoA
Uelo_7	3=Hydroxy=C26=CoA => Trans=2=C26=CoA
UGA1	4=Aminobutanoate + 2=Oxoglutarate => Succinate semialdehyde + L=Glutamate
UGA4_1	GABAxt + H+EXT => 4=Aminobutanoate
UGA4_2	ALAVxt + H+EXT => 5=Aminolevulinate
UGP1	D=Glucose 1=phosphate + UTP <=> UDPglucose + Pyrophosphate
URA1	(S)=Dihydroorotate + Oxygen <=> H2O2 + Orotate
URA10	Orotate + 5=Phospho=alpha=D=ribose 1=diphosphate <=> Pyrophosphate + Orotidine 5'=phosphate
URA2_1	Carbamoyl phosphate + L=Aspartate => N=Carbamoyl=L=aspartate + Orthophosphate
URA2_2	L=Glutamine + 2.000000 ATP + CO2 => L=Glutamate + Carbamoyl phosphate + 2.000000 ADP + Orthophosphate
URA3	Orotidine 5'=phosphate => CO2 + UMP
URA4	N=Carbamoyl=L=aspartate <=> (S)=Dihydroorotate
URA5	Orotate + 5=Phospho=alpha=D=ribose 1=diphosphate <=> Pyrophosphate + Orotidine 5'=phosphate
URA6_1	ATP + UMP <=> ADP + UDP
URA6_2	UMP + ATP <=> UDP + ADP
URA6_3	dUMP + ATP <=> dUDP + ADP
URA7_1	UTP + L=Glutamine + ATP => L=Glutamate + CTP + ADP + Orthophosphate
URA7_2	ATP + UTP + NH3 => ADP + Orthophosphate + CTP
URA8_1	UTP + L=Glutamine + ATP => L=Glutamate + CTP + ADP + Orthophosphate
URA8_2	ATP + UTP + NH3 => ADP + Orthophosphate + CTP
URH1_1	Guanosine => Guanine + D=Ribose
URH1_2	Adenosine => Adenine + D=Ribose
URK1_1	Uridine + GTP => UMP + GDP
URK1_2	Cytidine + GTP => GDP + CMP
URK1_3	Uridine + ATP => ADP + UMP
UTR1_1	NAD+ + ATP => NADP+ + ADP
UTR1_2	NAD+M + ATPM => NADP+M + ADPM
VAP1_1	CYSxt + H+EXT <=> L=Cysteine
VAP1_2	HISxt + H+EXT <=> L=Histidine
VAP1_3	ILExt + H+EXT <=> L=Isoleucine

VAP1_4	LEUxt + H+EXT <=> L=Leucine
VAP1_5	TRPxt + H+EXT <=> L=Tryptophan
VAP1_6	TYRxt + H+EXT <=> L=Tyrosine
VAP1_7	VALxt + H+EXT <=> L=Valine
VAS1	ATP + L=Valine + tRNA(Val) => AMP + Pyrophosphate + L=Valyl=tRNA(Val)
VHT1	BTxt + H+EXT <=> Biotin
VPS34	ATP + 1=Phosphatidyl=D=myo=inositol => ADP + 1=Phosphatidyl=D=myo=inositol=3=phosphate
WRS1	ATP + L=TryptophanM + tRNA(Trp) => AMP + Pyrophosphate + L=Tryptophanyl=tRNA(Trp)
XKS1	D=Xylulose + ATP => D=Xylulose 5=phosphate + ADP
XPT1	Xanthine + 5=Phospho=alpha=D=ribose 1=diphosphate => Xanthosine 5'=phosphate + Pyrophosphate
YAR075W	IMP + NAD+ => NADH + Xanthosine 5'=phosphate
YAT1	CoAM + O=AcetylcarnitineM => Acetyl=CoAM + CarnitineM
YAT2	Acetyl=CoA + Carnitine => CoA + O=Acetylcarnitine
YBL098W	L=Kynurenine + NADPH + Oxygen => 3=Hydroxykynurenine + NADP+
YBR006W	Succinate semialdehyde + NADP+ => Succinate + NADPH
YBR184W_1	Melibiose => alpha=D=Glucose + D=Galactose
YBR184W_2	alpha=D=Fucoside => alpha=D=Glucose + D=Galactose
YBR184W_3	Raffinose => D=Galactose + Sucrose
YBR184W_4	1=alpha=D=Galactosyl=myo=inositol <=> myo=Inositol + D=Galactose
YBR184W_5	Epimelibiose <=> alpha=D=Mannose + D=Galactose
YBR184W_6	Galactosylglycerol <=> Glycerol + D=Galactose
YBR184W_7	Melibiitol <=> D=Sorbitol + D=Galactose
YBR284W	AMP => Adenine + D=Ribose 5=phosphate
YCR024C	ATPM + L=Asparagine + tRNAM => AMPM + PyrophosphateM + L=Asparaginyl=tRNA(Asn)M
YDC1_1	D=Ceramide => Sphinganine
YDC1_2	P=Ceramide => Phytosphingosine
YDL100C	Dihydroneopterin phosphate => 2=Amino=4=hydroxy=6=(D=erythro=1,2,3=trihydroxypropyl)=7,8=dihydropteridine + Orthophosphate
YDR111C	Pyruvate + L=Glutamate <=> 2=Oxoglutarate + L=Alanine
YDR287W	1L=myo=Inositol 1=phosphate => myo=Inositol + Orthophosphate
YDR341C	ATP + L=Arginine + tRNA(Arg) => AMP + Pyrophosphate + L=Arginyl=tRNA(Arg)
YDR531W	(R)=Pantothenate + ATP => ADP + D=4'=Phosphopantothenate
YEA6	NAD+ => NAD+M
YEH1	Ergosterol=ester => Ergosterol + Acyl_acids
YEH2	Ergosterol=ester => Ergosterol + Acyl_acids

YEL041W_1	NAD+ + ATP => NADP+ + ADP
YEL041W_2	NAD+M + ATPM => NADP+M + ADPM
YEL047C	FADH2M + Fumarate => Succinate + FADM
YER053C	Orthophosphate + HO=M <=> OrthophosphateM
YER087W	ATP + L=ProlineM + tRNA(Pro)M => AMP + Pyrophosphate + L=Prolinyl=tRNA(Pro)M
YFL030W	L=Alanine + Glyoxylate <=> Pyruvate + Glycine
YFR055W	L=Cystathionine => Homocysteine + Pyruvate + NH3
YGL186C_1	CYTSxt + H+EXT => Cytosine
YGL186C_2	ADxt + H+EXT => Adenine
YGL186C_3	GNxt + H+EXT <=> Guanine
YGL245W	L=Glutamate + ATP => L=Glutamyl=tRNA(Glu) + AMP + Pyrophosphate
YGR012W	O=Acetyl=L=serine + Hydrogen sulfide => Acetate + L=Cysteine
YGR012W_1	ATPM + L=CysteineM + tRNA(Cys)M => AMP + PyrophosphateM + L=Cysteinyl=tRNA(Cys)M
YGR043C	D=Glyceraldehyde 3=phosphate + Sedoheptulose 7=phosphate <=> D=Erythrose 4=phosphate + beta=D=Fructose 6=phosphate
YGR125W	SLFxt => Sulfate
YGR287C	Maltose => 2.000000 alpha=D=Glucose
YHL012W	D=Glucose 1=phosphate + UTP <=> UDPglucose + Pyrophosphate
YHR020W	ATP + L=Proline + tRNA(Pro) => AMP + Pyrophosphate + L=Prolinyl=tRNA(Pro)
YIA6	NAD+ => NAD+M
YIL145C	(R)=Pantoate + beta=Alanine + ATP => AMP + Pyrophosphate + (R)=Pantothenate
YIL167W	L=Serine => Pyruvate + NH3
YIL172C	Maltose => 2.000000 alpha=D=Glucose
YJL068C	S=Formylglutathione <=> Glutathione + Formate
YJL070C	AMP => Adenine + D=Ribose 5=phosphate
YJL200C	CitrateM <=> IsocitrateM
YJL216C	Maltose => 2.000000 alpha=D=Glucose
YJR078W	L=Tryptophan + Oxygen => L=Formylkynurenine
YJR105W	Adenosine + ATP => AMP + ADP
YLR089C	PyruvateM + GlutamateM <=> 2=OxoglutarateM + L=AlanineM
YLR231C_1	L=Kynurenine => L=Alanine + Anthranilate
YLR231C_2	3=Hydroxykynurenine => 3=Hydroxyanthranilate + L=Alanine
YLR328W_1	Nicotinate D=ribonucleotide + ATP => Pyrophosphate + Deamino=NAD+
YLR328W_2	Nicotinate D=ribonucleotideM + ATPM => PyrophosphateM + Deamino=NAD+M
YLR328W_3	NMNm + ATPM => NAD+M + PyrophosphateM

YML082W	O=Succinyl=L=homoserine <=> Succinate + 2=Oxobutanoate + NH3
YMR1	1=Phosphatidyl=D=myo=inositol=3=phosphate => 1=Phosphatidyl=D=myo=inositol + Pyrophosphate
YMR293C	4=Guanidino=butanamide => 4=Guanidino=butanoate + NH3
YNK1_1	UDP + ATP <=> UTP + ADP
YNK1_2	CDP + ATP <=> CTP + ADP
YNK1_3	dGDP + ATP <=> dGTP + ADP
YNK1_4	dUDP + ATP <=> dUTP + ADP
YNK1_5	dCDP + ATP <=> dCTP + ADP
YNK1_6	dTDP + ATP <=> dTTP + ADP
YNK1_7	dADP + ATP <=> dATP + ADP
YNK1_8	GDP + ATP <=> GTP + ADP
YNK1_9	IDP + ATP <=> ITP + IDP
YNL247W	ATP + L=Cysteine + tRNA(Cys) => AMP + Pyrophosphate + L=Cysteinyl=tRNA(Cys)
YOR071C	THMxt + H+EXT => Thiamin
YOR192C	THMxt + H+EXT => Thiamin
YPC1_1	D=Ceramide => Sphinganine
YPC1_2	P=Ceramide => Phytosphingosine
YPL275W	Formate + NAD+ => CO2 + NADH
YPL276W	Formate + NAD+ => CO2 + NADH
YSR3_1	Sphinganine 1=phosphate => Sphinganine + Orthophosphate
YSR3_2	Phytosphingosine 1=phosphate => Phytosphingosine + Orthophosphate
YUR1	beta=D=Mannosyldiacetylchitobiosyldiphosphodolichol + 2.000000 GDPmannose => 2.000000 GDP + "(""alpha""=D=mannosyl)(,2)=""beta""=D=mannosyl=diacetylchitobiosyldiphosphod olichol"
ZWF1	alpha=D=Glucose 6=phosphate + NADP+ <=> D=Glucono=1,5=lactone 6=phosphate + NADPH
