# Supplementary material for: The genome-scale metabolic model iIN800 of Saccharomyces cerevisiae and its validation: a scaffold to query lipid metabolism
Source: BMC Syst Biol. 2008 Aug 7;2:71. doi: 10.1186/1752-0509-2-71 (PMC2542360; doi:10.1186/1752-0509-2-71)

S. cerevisiae genome-scale metabolic model iIN800

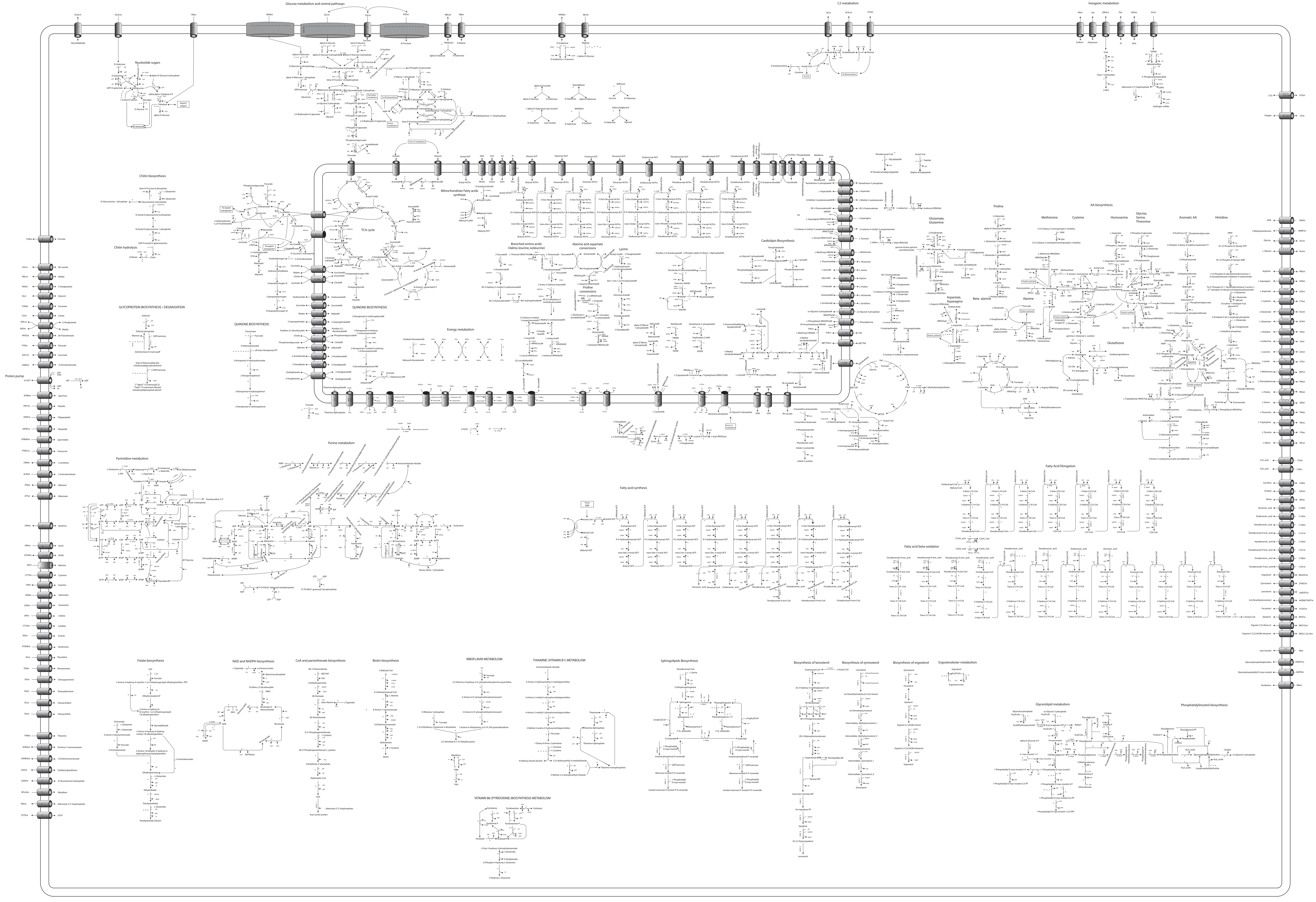

Supplement: Additional file 5 — Anaerobic Flux distribution. Visualization of flux distribution of anaerobic growth mapping on Figure 1. [file 1752-0509-2-71-S5.pdf]
